# Supplementary material for: Estimating the proportion of beneficial mutations that are not adaptive in mammals
Source: PLoS Genet. 2024 Dec 26;20(12):e1011536. doi: 10.1371/journal.pgen.1011536 (PMC11709321; doi:10.1371/journal.pgen.1011536)
Supplement: S1 File — Contains 5 pages of supplementary information including 1 figure (Fig A). (PDF) [file pgen.1011536.s001.pdf]

# Estimating the proportion of beneficial mutations that are not adaptive in mammals

**T. Latrille[1†], J. Joseph[2†], D. A. Hartasánchez[1], N. Salamin[1]**

[1]Department of Computational Biology, Université de Lausanne, Lausanne, Switzerland

[2]Laboratoire de Biométrie et Biologie Evolutive, UMR5558, Université Lyon 1, Villeurbanne, France

[†]These authors contributed equally to this work

thibault.latrille@ens-lyon.org

# S1 File

## Contents

**1 Mutation-selection codon models** **2**

1.1 Prior distributions and parameters of the model . . . 2

1.2 Nucleotide mutation rates . . . 2

1.3 Site-specific amino-acid fitness profiles . . . 3

1.4 Branch length . . . 3

1.5 Codon substitution rates . . . 4

1.6 Bayesian implementation . . . 4

1.7 BayesCode software . . . 5

## List of Figures

A Bayesian hierarchical model. . . . 2

# 1 Mutation-selection codon models

## 1.1 Prior distributions and parameters of the model

We here present the Mutation-selection codon model as a Bayesian hierarchical model, including the prior distributions and the parameters of the model. This hierarchical model is formally represented as directed acyclic graph of dependencies between variables, depicted in Figure A:

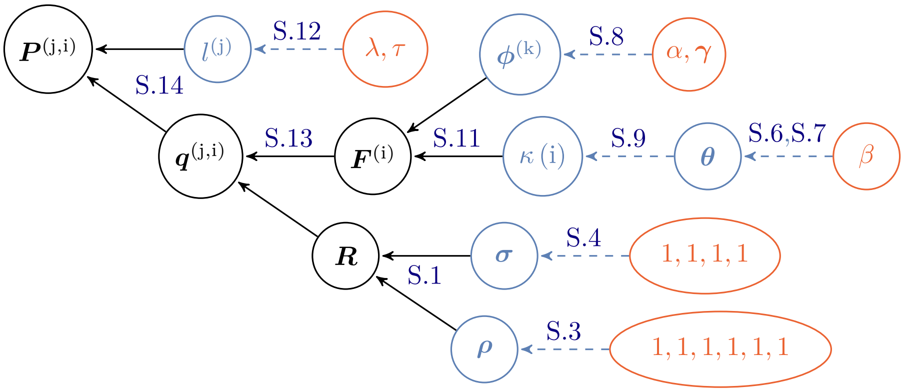


Figure A: **Bayesian hierarchical model.** Nodes of the directed acyclic graph are the variables, and edges are the functions. Hyper-parameters are depicted in red circles, random variables in blue circles, and transformed variables in black. Blue dashed line denotes a drawing from a random distribution, and black solid lines denote a function. All the nodes pointing toward a given node (upstream) are its dependencies which determine its distribution. The other way around, following the arrows in the DAG (downstream), simple prior distributions are combined together to form more complex joint prior distributions which ultimately define the prior distribution of the model. Equations S.1 to S.14 describe the relationship between variables (sections 1.2 to 1.5).

## 1.2 Nucleotide mutation rates

The generalized time-reversible (GTR) nucleotide mutation rate matrix $\boldsymbol{R}$ is a function of the nucleotide frequencies $\boldsymbol{\sigma}$ and the symmetric exchangeability rates $\boldsymbol{\rho}$ [1]. $\boldsymbol{\sigma} = (\sigma_A, \sigma_C, \sigma_G, \sigma_T)$ is the equilibrium base frequency vector, giving the frequency at which each base occurs at each site. $\boldsymbol{\rho} = (\rho_{AC}, \rho_{AG}, \rho_{AT}, \rho_{CG}, \rho_{CT}, \rho_{GT})$ is the vector of exchangeabilities between nucleotides. Altogether, the rate matrix is:

$$\boldsymbol{R} = \begin{matrix} \\ A \\ C \\ G \\ T \end{matrix} \begin{matrix} \begin{matrix} A & \quad C & \quad G & \quad T \end{matrix} \\ \begin{pmatrix} -\mu_A & \rho_{AC}\sigma_C & \rho_{AG}\sigma_G & \rho_{AT}\sigma_T \\ \rho_{AC}\sigma_A & -\mu_C & \rho_{CG}\sigma_G & \rho_{CT}\sigma_T \\ \rho_{AG}\sigma_A & \rho_{CG}\sigma_C & -\mu_G & \rho_{GT}\sigma_T \\ \rho_{AT}\sigma_A & \rho_{CT}\sigma_C & \rho_{GT}\sigma_G & -\mu_T \end{pmatrix} \end{matrix} \tag{S.1}$$

By definition, the sum of the entries in each row of the nucleotide rate matrix $\boldsymbol{R}$ is equal to 0, giving the diagonal entries:

$$\mu_a = \sum_{b \neq a, b \in \{A,C,G,T\}} R_{a,b} \tag{S.2}$$

The prior on the exchangeabilities $\boldsymbol{\rho}$ is a uniform Dirichlet distribution of dimension 6:

$$\boldsymbol{\rho} \sim \mathrm{Dir}\left(1, 1, 1, 1, 1, 1\right). \tag{S.3}$$

The prior on the equilibrium base frequencies $\boldsymbol{\sigma}$ is a uniform Dirichlet distribution of dimension 4:

$$\boldsymbol{\sigma} \sim \text{Dir}\left(1, 1, 1, 1\right) \tag{S.4}$$

The general time-reversible nucleotide matrix is normalized with a total flow of 1:

$$\sum_{a \in \{A,C,G,T\}} -\sigma_a R_{a,a} = 1, \tag{S.5}$$

such that we expect 1 substitution per unit of branch length.

## 1.3 Site-specific amino-acid fitness profiles

Site-specific amino-acid fitness profiles are assumed i.i.d. from a mixture model, itself endowed with a truncated Dirichlet process prior. Specifically, the mixture has K components (K = 30 by default). The prior on component weights ($\boldsymbol{\theta}$) is modeled using a stick-breaking process, truncated at K and of parameter $\beta = 1$:

$$\begin{aligned} &\boldsymbol{\theta} \sim \text{StickBreaking}\left(\text{K}, \beta\right) \\ \iff &\theta_{\text{k}} = \psi_{\text{k}} \cdot \prod_{l=1}^{\text{k}-1} \left(1 - \psi_l\right), \ \text{k} \in \{1, \dots, \text{K}\}, \end{aligned} \tag{S.6}$$

where $\psi_{\text{k}}$ are i.i.d. from a beta distribution

$$\psi_{\text{k}} \sim \text{Beta}\left(1, \beta\right), \ \text{k} \in \{1, \dots, \text{K}\}. \tag{S.7}$$

Of note, the weights decrease geometrically in expectation, at rate $\beta$, such that lower values of $\beta$ induce more heterogeneous distributions of weights.

Each component of the mixture defines a 20-dimensional fitness profile $\boldsymbol{\phi}^{(\text{k})}$ (summing to 1), for $\text{k} \in \{1, \dots, \text{K}\}$. These fitness profiles are i.i.d. from a Dirichlet of center $\boldsymbol{\gamma} = 1$ and concentration $\alpha = 1$:

$$\boldsymbol{\phi}^{(\text{k})} \sim \text{Dir}\left(\boldsymbol{\gamma}, \ \alpha\right), \ \text{k} \in \{1, \dots, \text{K}\}. \tag{S.8}$$

Site allocations to the mixture components $\kappa\left(\text{i}\right) \in \{1, \dots, \text{K}\}$, for $\text{i} \in \{1, \dots, \text{N}\}$ running over the N sites of the alignment, are i.i.d. multinomial of parameter $\boldsymbol{\theta}$:

$$\boldsymbol{m} \sim \text{Multinomial}\left(\boldsymbol{\theta}\right), \tag{S.9}$$

$$\text{where } m_{\text{k}} = \sum_{\text{i} \in \{1, \dots, \text{N}\}} \mathbb{1}_{\kappa(\text{i}) = \text{k}} \tag{S.10}$$

For a given parameter configuration for the mixture, the scaled fitness $\boldsymbol{F}^{(\text{i})}$ at site i, are obtained by taking the logarithm of fitness assigned to this site:

$$\boldsymbol{F}^{(\text{i})} = \ln\left(\boldsymbol{\phi}^{(\kappa(\text{i}))}\right), \ \text{i} \in \{1, \dots, \text{N}\}. \tag{S.11}$$

## 1.4 Branch length

The topology of the rooted phylogenetic tree is supposed to be known and is not estimated by the model. The branch lengths $l^{(\text{j})}$ are defined as the expected number of neutral substitutions per DNA site along a branch, each from a Gamma distribution of mean $\lambda = 0.1$ and scale $\tau = 1$:

$$l^{(\text{j})} \sim \text{Gamma}\left(\lambda, \tau\right). \tag{S.12}$$

## 1.5 Codon substitution rates

The mutation rate between codons $a$ and $b$, denoted $\mu_{a\mapsto b}$ depends on the underlying nucleotide change between the codons. First, if codons $a$ and $b$ are not nearest-neighbours, $\mu_{a\mapsto b}$ is equal to 0. Second, if codons $a$ and $b$ are only one mutation away, $\mu_{a\mapsto b}$ is given by the underlying nucleotide relative rate ($R_{a\mapsto b}$).

For a given site i, the codon substitution rate matrix $\boldsymbol{q}^{(\mathrm{i})}$ is given by:

$$\begin{cases} q^{(\mathrm{i})}_{a\mapsto b} = 0 \text{ if codons } a \text{ and } b \text{ are not nearest-neighbors,} \\ q^{(\mathrm{i})}_{a\mapsto b} = \mu_{a\mapsto b} \text{ if codons } a \text{ and } b \text{ are synonymous,} \\ q^{(\mathrm{i})}_{a\mapsto b} = \mu_{a\mapsto b} \dfrac{F^{(\mathrm{i})}_b - F^{(\mathrm{i})}_a}{1 - \mathrm{e}^{F^{(\mathrm{i})}_a - F^{(\mathrm{i})}_b}} \text{ if codons } a \text{ and } b \text{ are non-synonymous,} \\ q^{(\mathrm{i})}_{a,a} = - \sum\limits_{b\neq a, b=1}^{61} q^{(\mathrm{i})}_{a\mapsto b}. \end{cases} \tag{S.13}$$

Together, the probability of transition between codons for a given branch j and site i is:

$$\boldsymbol{P}^{(\mathrm{j,i})} = \mathrm{e}^{l^{(\mathrm{j})}\boldsymbol{q}^{(\mathrm{i})}}, \tag{S.14}$$

which are the matrices necessary to compute the likelihood of the data ($D$) given the parameters of the model using the pruning algorithm.

## 1.6 Bayesian implementation

Bayesian inference was conducted using Markov Chain Monte Carlo (MCMC). Most phylogenetic MCMC samplers target the distribution over the model parameters given the sequence alignment, which means that they have to repeatedly invoke the pruning algorithm to recalculate the likelihood which is most often the limiting step of the MCMC. An alternative, which is used here, is to do the MCMC conditionally on the detailed substitution history $\mathcal{H}$, thus doing the MCMC over the augmented configuration ($\mathcal{H}$, $D$), under the target distribution obtained by combining the mapping-based likelihood with the prior over model parameters. The key idea that makes this strategy efficient is that the mapping-based likelihood depends on compact summary statistics of $\mathcal{H}$, leading to a very fast evaluation of the likelihood. On the other hand, this requires to implement more complex MCMC procedures that have to alternate between:

1. sampling $\mathcal{H}$ conditionally on the data and the current parameter configuration.

2. re-sampling the parameters conditionally on $\mathcal{H}$.

To implement the mapping-based MCMC sampling strategy, we first sample the detailed substitution history $\mathcal{H}$ for all sites along the tree. Several methods exist for doing this [2, 3], which are used here in combination: first trying the accept-reject method of Nielsen [2], then switching to the uniformization approach of Rodrigue *et al.* [3] if the first round has failed.

Then, we write down the probability of $\mathcal{H}$ given the parameters, and finally, we collect all factors that depend on some parameter of interest and make some simplifications. This ultimately leads to relatively compact sufficient statistics allowing for fast numerical evaluation of the likelihood [4, 5].

### 1.7 BayesCode software

In *BayesCode* (github.com/ThibaultLatrille/BayesCode, v1.3.1), we ran the mutation-selection codon models *mutselomega* for 2000 points of MCMC with the options:

```
mutselomega ---omegashift 0.0 --ncat 30 -a my_alignment.phy -t my_tree.newick -u 2000 my_genename
```

The collection of site-specific fitness profiles ($\boldsymbol{F}^{(i)}, \forall i$) are then obtained by running *readmutselomega*, reading 1000 points of MCMC (first 1000 are considered as burn-in) with the options:

```
readmutselomega --every 1 --until 2000 --burnin 1000 --ss my_genename
```

The gene-specific $4 \times 4$ nucleotide mutation rate matrix ($\boldsymbol{\mu}$) is also obtained by running *readmutselomega*, reading 1000 points of MCMC (first 1000 are considered as burn-in) with the options:

```
readmutselomega --every 1 --until 2000 --burnin 1000 --nuc my_genename
```

## References


1. Tavaré, S. Some Probabilistic and Statistical Problems in the Analysis of DNA Sequences. *Lectures on mathematics in the life sciences* **17,** 57–86 (1986).
2. Nielsen, R. Mapping Mutations on Phylogenies. *Systematic Biology* **51,** 729–739 (2002).
3. Rodrigue, N., Philippe, H. & Lartillot, N. Uniformization for Sampling Realizations of Markov Processes: Applications to Bayesian Implementations of Codon Substitution Models. *Bioinformatics* **24,** 56–62 (2008).
4. Irvahn, J. & Minin, V. N. Phylogenetic Stochastic Mapping without Matrix Exponentiation. *Journal of Computational Biology* **21,** 676–690 (2014).
5. Davydov, I. I., Robinson-Rechavi, M. & Salamin, N. State Aggregation for Fast Likelihood Computations in Molecular Evolution. *Bioinformatics* **33,** 354–362 (2017).
