# Supplementary material for: Estimating the proportion of beneficial mutations that are not adaptive in mammals
Source: PLoS Genet. 2024 Dec 26;20(12):e1011536. doi: 10.1371/journal.pgen.1011536 (PMC11709321; doi:10.1371/journal.pgen.1011536)
Supplement: S2 File — Contains 12 pages of supplementary information including 3 figures (Fig A to C) and 6 tables (Table A to F). (PDF) [file pgen.1011536.s002.pdf]

Estimating the proportion of beneficial mutations that are not adaptive in mammals

T. Latrille[1†], J. Joseph[2†], D. A. Hartasánchez[1], N. Salamin[1]

[1]Department of Computational Biology, Université de Lausanne, Lausanne, Switzerland

[2]Laboratoire de Biométrie et Biologie Evolutive, UMR5558, Université Lyon 1, Villeurbanne, France

[†]These authors contributed equally to this work

thibault.latrille@ens-lyon.org

S2 File

Contents

1 Beneficial mutations in the terminal lineages and populations **3**

1.1 Example sites . . . 3

1.2 Selection along the terminal branches . . . 6

1.2.1 Probability of mutations and substitutions to be $\mathcal{D}_0$, $\mathcal{N}_0$ or $\mathcal{B}_0$ . . . 6

1.2.2 d_N/d_S for $\mathcal{D}_0$, $\mathcal{N}_0$ or $\mathcal{B}_0$ . . . 7

1.2.3 d_N/d_S over-estimation due to non-adaptive beneficial mutations . . . 8

2 Gene ontology enrichment **9**

2.1 Gene ontology enrichment in $\mathcal{B}_0$ SNPs . . . 9

2.2 Enrichment in $\mathcal{B}_0$ SNPs across all gene ontology terms . . . 11

3 Clinically related terms for mutations **12**

3.1 Terms associated with deleterious mutations $\mathcal{D}_0$ . . . 12

3.2 Terms associated with non-adaptive beneficial mutations $\mathcal{B}_0$ . . . 12

List of Figures

A Examples sites of $\mathcal{B}_0$ mutations in *Chlorocebus sabaeus* (reversions). . . . 4
B Examples sites of $\mathcal{B}_0$ mutations in *Chlorocebus sabaeus*. . . . 5
C Enrichment in $\mathcal{B}_0$ SNPs across all gene ontology terms. . . . 11

List of Tables

A Probability of mutations and substitutions to be $\mathcal{D}_0$, $\mathcal{N}_0$ or $\mathcal{B}_0$. . . . 6
B d_N/d_S for $\mathcal{D}_0$, $\mathcal{N}_0$ or $\mathcal{B}_0$. . . . 7
C d_N/d_S over-estimation due to non-adaptive beneficial mutations. . . . 8
D Gene ontology enrichment in $\mathcal{B}_0$ SNPs. . . . 10
E Terms associated with deleterious mutations $\mathcal{D}_0$. . . . 12
F Terms associated with non-adaptive beneficial mutations $\mathcal{B}_0$. . . . 12

1 Beneficial mutations in the terminal lineages and populations

1.1 Example sites

We extracted protein-coding DNA alignments across mammals centered around the codon site (two flanking codons in white background) for which the beneficial non-adaptive mutations ($\mathcal{B}_0$) have been detected in *Chlorocebus sabaeus*. We also show the translated amino acids of this region. For instance, in DNA alignment of gene SELE (A), the nucleotide at site 1722 has mutated (from T to C) at the basis of Simiiformes (monkeys and apes), modifying the corresponding amino acid from Serine to Proline, but has been subsequently reverted in the branch of *Chlorocebus sabaeus*. However, other substitutions classified as $\mathcal{B}_0$ cannot be clearly interpreted as reversions *sensu stricto* along the terminal branch of *Chlorocebus sabaeus*. Indeed, we acknowledge that on a fixed fitness landscape, a deleterious mutation can be compensated by transitions to other fitter amino-acids, and not necessarily the ancestral one.

Finally, we generated the fasta alignment for all $\mathcal{B}_0$ mutations that we detected, either in the terminal lineage or in segregating polymorphisms. These fasta files are available at Zenodo (https://doi.org/10.5281/zenodo.7878953), across all populations, under a zip file (*alignment_around_non_adaptive_mutations.zip*) alongside a python script to obtain the figures (as in Figures A and B) given these alignments (*plot_variations.py*).

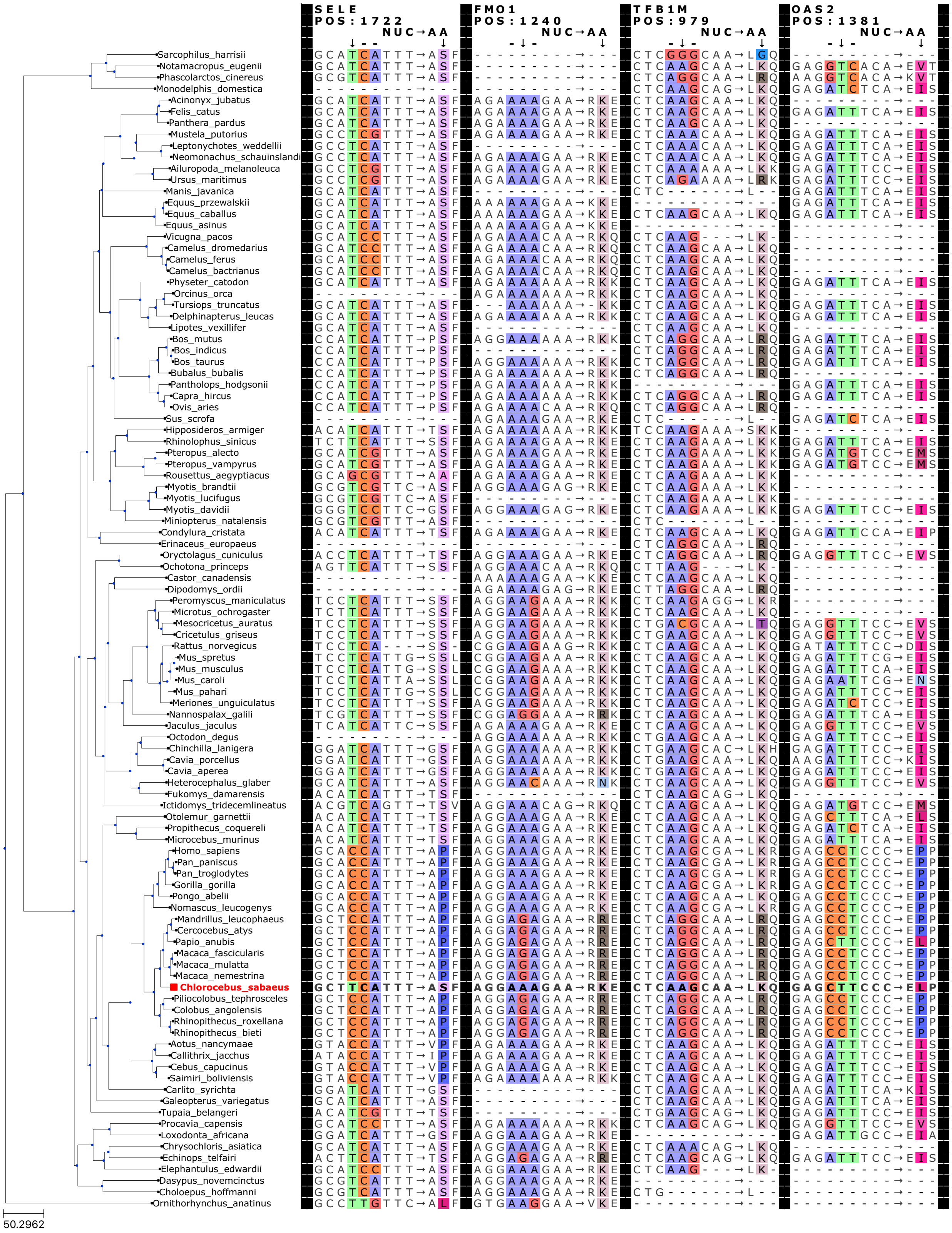

Figure A: **Examples sites of $\mathcal{B}_0$ mutations in *Chlorocebus sabaeus* (reversions).** The first line is the name of the gene, the second line the position of the mutation in the OrthoMam protein-coding DNA alignment, and the arrows in the third line correspond to the position of the mutation in the nucleotide sequence (left) and in the corresponding amino acid sequence (right)

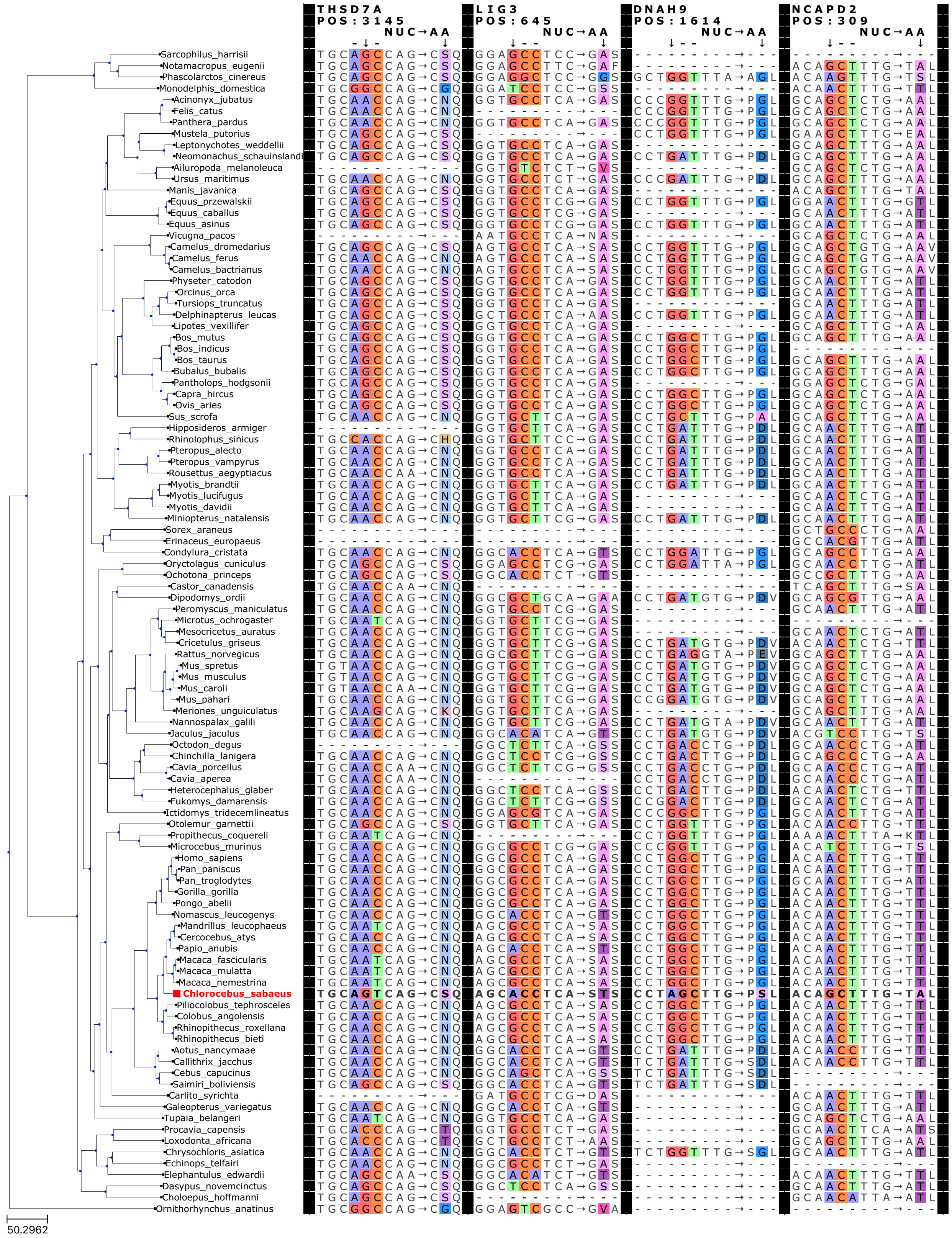

Figure B: **Examples sites of $\mathcal{B}_0$ mutations in *Chlorocebus sabaeus*.** The first line is the name of the gene, the second line the position of the mutation in the OrthoMam protein-coding DNA alignment, and the arrows in the third line correspond to the position of the mutation in the nucleotide sequence (left) and in the corresponding amino acid sequence (right).

1.2 Selection along the terminal branches

1.2.1 Probability of mutations and substitutions to be $\mathcal{D}_0$, $\mathcal{N}_0$ or $\mathcal{B}_0$

Among all the substitutions found in each terminal branch, between 10 and 13% were $\mathcal{B}_0$ ($\mathbb{P}_{div}[\mathcal{B}_0]$), while $\mathcal{B}_0$ mutations only represent between 0.9 and 1.2% of all non-synonymous mutations ($\mathbb{P}[\mathcal{B}_0]$), as shown in Table A.

Population	Species	$\mathbb{P}[\mathcal{D}_0]$	$\mathbb{P}[\mathcal{N}_0]$	$\mathbb{P}[\mathcal{B}_0]$	$\mathbb{P}_{div}[\mathcal{D}_0]$	$\mathbb{P}_{div}[\mathcal{N}_0]$	$\mathbb{P}_{div}[\mathcal{B}_0]$
Equus c.	Equus caballus	0.923	0.065	0.012	0.462	0.419	0.118
Iran	Bos taurus	0.924	0.065	0.011	0.515	0.362	0.123
Uganda	Bos taurus	0.924	0.065	0.011	0.514	0.361	0.125
Australia	Capra hircus	0.923	0.066	0.011	0.494	0.386	0.121
France	Capra hircus	0.923	0.066	0.011	0.494	0.386	0.120
Iran (C. aegagrus)	Capra hircus	0.923	0.066	0.011	0.493	0.386	0.120
Iran	Capra hircus	0.923	0.066	0.011	0.492	0.387	0.121
Italy	Capra hircus	0.923	0.066	0.011	0.494	0.386	0.120
Morocco	Capra hircus	0.923	0.066	0.011	0.491	0.387	0.122
Iran	Ovis aries	0.922	0.067	0.012	0.568	0.323	0.109
Iran (O. orientalis)	Ovis aries	0.922	0.067	0.011	0.573	0.320	0.108
Iran (O. vignei)	Ovis aries	0.922	0.067	0.012	0.567	0.325	0.109
Various	Ovis aries	0.922	0.067	0.011	0.572	0.321	0.107
Morocco	Ovis aries	0.922	0.067	0.012	0.570	0.321	0.108
Barbados	Chlorocebus sabaeus	0.926	0.065	0.009	0.485	0.393	0.122
Central Afr. Rep.	Chlorocebus sabaeus	0.926	0.065	0.009	0.485	0.391	0.124
Ethiopia	Chlorocebus sabaeus	0.926	0.065	0.009	0.484	0.393	0.124
Gambia	Chlorocebus sabaeus	0.926	0.065	0.009	0.483	0.394	0.123
Kenya	Chlorocebus sabaeus	0.926	0.065	0.009	0.485	0.392	0.123
Nevis	Chlorocebus sabaeus	0.926	0.065	0.009	0.484	0.393	0.123
South Africa	Chlorocebus sabaeus	0.926	0.065	0.009	0.480	0.394	0.125
Saint Kitts	Chlorocebus sabaeus	0.926	0.065	0.009	0.483	0.394	0.123
Zambia	Chlorocebus sabaeus	0.926	0.065	0.009	0.485	0.393	0.123
African	Homo sapiens	0.925	0.065	0.010	0.561	0.341	0.099
Admixed American	Homo sapiens	0.925	0.065	0.010	0.561	0.340	0.099
East Asian	Homo sapiens	0.925	0.065	0.010	0.560	0.341	0.098
European	Homo sapiens	0.925	0.065	0.010	0.562	0.340	0.098
South Asian	Homo sapiens	0.925	0.065	0.010	0.561	0.341	0.099

Table A: **Probability of mutations and substitutions to be $\mathcal{D}_0$, $\mathcal{N}_0$ or $\mathcal{B}_0$.** $\mathbb{P}[\mathcal{D}_0]$ (eq. 5) is the probability for a new mutation to be deleterious. These mutations have a selection coefficient predicted at the phylogenetic-scale lower than -1, thus toward a less fit amino-acid. $\mathbb{P}[\mathcal{N}_0]$ (eq. 5) is the probability for a new mutation to be nearly-neutral. These mutations have a selection coefficient predicted at the phylogenetic-scale between -1 and 1. $\mathbb{P}[\mathcal{B}_0]$ (eq. 5) is the probability for a new mutation to be non-adaptive beneficial. These mutations have a selection coefficient predicted at the phylogenetic-scale larger than 1, thus toward a more fit amino-acid. $\mathbb{P}_{div}[\mathcal{D}_0]$ is the proportion of substitutions in the terminal branch that are $\mathcal{D}_0$. $\mathbb{P}_{div}[\mathcal{N}_0]$ is the proportion of substitutions in the terminal branch that are $\mathcal{N}_0$. $\mathbb{P}_{div}[\mathcal{B}_0]$ is the proportion of substitutions in the terminal branch that are $\mathcal{B}_0$.

1.2.2 d_N/d_S for $\mathcal{D}_0$, $\mathcal{N}_0$ or $\mathcal{B}_0$

Theoretically, $\omega = d_N/d_S$ can be related to the underlying scaled selection coefficient (S) with the relation $\omega = S/(1-\exp(-S))$ as in [1, eq. 3]. In our experiments, our observed $d_N(\mathcal{B}_0)/d_S$ is within the range of 1.169 (*C. hircus*) to 1.745 (*H. sapiens*), as shown in Table B, translating into an average S of ≈ 0.32 to ≈ 1.24, so indeed only slightly advantageous.

Of note, observing $d_N(\mathcal{B}_0)/d_S > 1$ is an important check that $\mathcal{B}_0$ mutations are indeed positively selected with an increased substitution rate. Since $d_N(\mathcal{N}_0)/d_S$ is close to 1 for the predicted nearly-neutral mutations, this means that the selection coefficients predicted at the mutation-selection balance are good proxies of selection, then observing $d_N(\mathcal{B}_0)/d_S > 1$ is evidence of positive selection of predicted non-adaptive beneficial mutations.

Species	d_N/d_S	$d_N(\mathcal{D}_0)/d_S$	$d_N(\mathcal{N}_0)/d_S$	$d_N(\mathcal{B}_0)/d_S$
Equus caballus	0.129	0.065	0.832	1.267
Bos taurus	[0.114, 0.116]	[0.063, 0.064]	[0.629, 0.638]	[1.280, 1.328]
Capra hircus	[0.108, 0.109]	[0.058, 0.058]	[0.631, 0.636]	[1.169, 1.183]
Ovis aries	[0.127, 0.129]	[0.078, 0.080]	[0.619, 0.621]	[1.201, 1.217]
Chlorocebus sabaeus	[0.118, 0.119]	[0.062, 0.062]	[0.713, 0.720]	[1.521, 1.577]
Homo sapiens	[0.170, 0.170]	[0.103, 0.103]	[0.884, 0.888]	[1.733, 1.745]

Table B: **d_N/d_S for $\mathcal{D}_0$, $\mathcal{N}_0$ or $\mathcal{B}_0$.** d_N/d_S (eq. 6) is the ratio of non-synonymous over synonymous substitutions estimated for all the non-synonymous substitutions in the terminal branch. $d_N(\mathcal{D}_0)/d_S$ (eq. 6) is the ratio of non-synonymous over synonymous substitutions, when restricted to non-synonymous substitutions in the terminal branch that are $\mathcal{D}_0$. $d_N(\mathcal{N}_0)/d_S$ (eq. 6) is the ratio of non-synonymous over synonymous substitutions, when restricted to non-synonymous substitutions in the terminal branch that are $\mathcal{N}_0$. $d_N(\mathcal{B}_0)/d_S$ (eq. 6) is the ratio of non-synonymous over synonymous substitutions, when restricted to non-synonymous substitutions in the terminal branch that are $\mathcal{B}_0$. SNPs are considered fixed (as a substitution) in the populations if all sampled individuals are homozygous for the derived allele. This effect results in d_N/d_S varying across populations, denoted as a range per species. $d_N(\mathcal{B}_0)/d_S$ values observed are above 1 and are consistent with slightly advantageous mutations.

1.2.3 d_N/d_S over-estimation due to non-adaptive beneficial mutations

We estimated that between $\approx$ 9 and $\approx$ 12% of d_N/d_S is over-estimated, corresponding to non-adaptive beneficial mutations inflating the d_N/d_S statistic, as shown in Table C.

Species	d_N/d_S	$d_N(S_0 < 1)/d_S$	$\delta(d_N/d_S)$
Equus caballus	0.129	0.115	10.7
Bos taurus	[0.114, 0.116]	[0.101, 0.102]	[11.3, 11.5]
Capra hircus	[0.108, 0.109]	[0.096, 0.097]	[11.0, 11.2]
Chlorocebus sabaeus	[0.118, 0.119]	[0.104, 0.105]	[11.4, 11.7]
Homo sapiens	[0.170, 0.170]	[0.154, 0.155]	[8.926, 8.996]
Ovis aries	[0.127, 0.129]	[0.115, 0.117]	[9.663, 9.894]

Table C: **d_N/d_S over-estimation due to non-adaptive beneficial mutations.** d_N/d_S (eq. 6) is the ratio of non-synonymous over synonymous substitutions estimated for all the non-synonymous substitutions in the terminal branch. $d_N(S_0 < 1)/d_S$ (eq. 6) is the ratio of non-synonymous over synonymous substitutions, when restricted to non-synonymous substitutions in the terminal branch that are not $\mathcal{B}_0$. This is the estimated divergence when we remove non-adaptive beneficial mutations. $\delta(d_N/d_S)$ (eq. 7) is the fraction of the divergence (d_N/d_S) that is over-estimated: the difference between d_N/d_S and $d_N(S_0 < 1)/d_S$.

2 Gene ontology enrichment

2.1 Gene ontology enrichment in $\mathcal{B}_0$ SNPs

To assess whether $\mathcal{B}_0$ SNPs are enriched in specific gene functions, we used gene ontology (GO) terms at the gene level. Thus, for each $\mathcal{B}_0$ SNP detected in a focal population, we annotated this SNP with GO terms inherited from the gene in which the SNP is located. We restricted this analysis solely on genes annotated with at least one GO term. To assess the weight of each GO term in the set of $\mathcal{B}_0$ SNPs, we computed the proportion of $\mathcal{B}_0$ SNPs annotated with a given GO term. We then tested if the distribution of $\mathcal{B}_0$ SNPs is different between genes sharing a common GO term and the rest of the genes, as shown in Table D.

GO id	GO name	p	r	Mann-Whitney U	p_v	p_v^{adj}
GO:0003777	microtubule motor activity	0.027	1.802	1.1×10^5	1.8×10^{-6}	**0.0008***
GO:0005578	proteinaceous extracellular matrix	0.069	2.071	4.7×10^5	1.9×10^{-6}	**0.00084***
GO:0003774	motor activity	0.023	1.127	1.1×10^5	6.7×10^{-5}	**0.029***
GO:0030198	extracellular matrix organization	0.039	1.956	2.7×10^5	0.00055	0.239
GO:0007018	microtubule-based movement	0.023	0.924	1.3×10^5	0.00073	0.315
GO:0005576	extracellular region	0.178	2.195	1.9×10^6	0.00083	0.359
GO:0006898	receptor-mediated endocytosis	0.027	1.803	1.7×10^5	0.001	0.512
GO:0006355	regulation of transcription	0.069	0.275	1.9×10^6	0.002	0.815
GO:0005604	basement membrane	0.019	1.638	1.1×10^5	0.002	0.852
GO:0007229	integrin-mediated signaling pathway	0.019	1.930	1.1×10^5	0.003	1.000
GO:0016887	ATPase activity	0.031	1.149	1.8×10^5	0.003	1.000
GO:0007155	cell adhesion	0.062	1.438	5.8×10^5	0.005	1.000
GO:0005634	nucleus	0.266	0.557	3.9×10^6	0.005	1.000
GO:0006351	transcription	0.073	0.339	1.9×10^6	0.008	1.000
GO:0005654	nucleoplasm	0.124	0.443	2.6×10^6	0.009	1.000
GO:0006366	transcription from RNA polymerase II promoter	0.008	0.063	6.2×10^5	0.011	1.000
GO:0005829	cytosol	0.212	0.541	3.5×10^6	0.012	1.000
GO:0016853	isomerase activity	0.015	4.066	1×10^5	0.015	1.000
GO:0071356	cellular response to tumor necrosis factor	0.015	7.069	1×10^5	0.016	1.000
GO:0005518	collagen binding	0.015	2.900	1×10^5	0.017	1.000
GO:0043087	regulation of GTPase activity	0.015	2.025	1.1×10^5	0.027	1.000
GO:0000139	Golgi membrane	0.012	0.214	6.3×10^5	0.028	1.000
GO:0043235	receptor complex	0.023	0.935	1.9×10^5	0.028	1.000
GO:0000981	RNA polymerase II transcription factor activity	0.027	0.452	9.1×10^5	0.029	1.000
GO:0005739	mitochondrion	0.058	0.471	1.4×10^6	0.029	1.000
GO:0005581	collagen trimer	0.015	2.124	1.1×10^5	0.036	1.000
GO:0051015	actin filament binding	0.019	0.912	1.5×10^5	0.036	1.000
GO:0001843	neural tube closure	0.015	1.164	1.1×10^5	0.036	1.000
GO:0005737	cytoplasm	0.317	0.692	4.1×10^6	0.037	1.000
GO:0043565	sequence-specific DNA binding	0.015	0.308	6×10^5	0.039	1.000
GO:0004222	metalloendopeptidase activity	0.019	1.188	1.6×10^5	0.043	1.000
GO:0007166	cell surface receptor signaling pathway	0.031	2.871	3×10^5	0.046	1.000
GO:0007156	homophilic cell adhesion via plasma membrane...	0.015	1.013	1.2×10^5	0.050	1.000
GO:0007286	spermatid development	0.015	2.497	1.2×10^5	0.051	1.000
GO:0008202	steroid metabolic process	0.015	2.755	1.2×10^5	0.051	1.000
GO:0061024	membrane organization	0.019	1.516	1.6×10^5	0.052	1.000
GO:1901796	regulation of signal transduction by p53 class...	0.019	1.333	1.6×10^5	0.058	1.000
GO:0006397	mRNA processing	0.004	0.210	3.5×10^5	0.061	1.000
GO:0006915	apoptotic process	0.015	0.463	6.4×10^5	0.065	1.000
GO:0043547	positive regulation of GTPase activity	0.035	2.651	3.7×10^5	0.068	1.000
GO:0007010	cytoskeleton organization	0.019	1.771	1.7×10^5	0.068	1.000
GO:0030308	negative regulation of cell growth	0.015	3.055	1.3×10^5	0.070	1.000
GO:0007160	cell-matrix adhesion	0.015	1.046	1.2×10^5	0.073	1.000
GO:0006897	endocytosis	0.023	1.408	2.2×10^5	0.073	1.000
GO:0003677	DNA binding	0.077	0.448	1.7×10^6	0.075	1.000
GO:0043202	lysosomal lumen	0.015	1.522	1.3×10^5	0.075	1.000
GO:0003700	DNA binding transcription factor activity	0.027	0.343	8.7×10^5	0.076	1.000
GO:0008134	transcription factor binding	0.004	0.048	3.2×10^5	0.077	1.000
GO:0005488	binding	0.046	0.641	4.1×10^5	0.078	1.000
GO:0045944	positive regulation of transcription from RNA...	0.039	0.324	1.1×10^6	0.085	1.000
GO:0005794	Golgi apparatus	0.046	0.430	1.2×10^6	0.087	1.000
GO:0016032	viral process	0.008	0.321	4.1×10^5	0.089	1.000
GO:0055085	transmembrane transport	0.054	1.533	5.9×10^5	0.092	1.000
GO:0006260	DNA replication	0.019	1.051	1.8×10^5	0.096	1.000
GO:0030425	dendrite	0.008	0.121	3.9×10^5	0.099	1.000
GO:0005524	ATP binding	0.127	0.666	1.5×10^6	0.103	1.000
GO:0005615	extracellular space	0.100	1.699	1.3×10^6	0.107	1.000

Continued on next page

GO id	GO name	p	r	Mann-Whitney U	p_v	p_v^{adj}
GO:0007283	spermatogenesis	0.039	1.824	3.9×10^5	0.108	1.000
GO:0005179	hormone activity	0.012	7.683	9.9×10^4	0.118	1.000
GO:0098793	presynapse	0.012	2.065	9.6×10^4	0.119	1.000
GO:0005254	chloride channel activity	0.012	1.694	9.6×10^4	0.124	1.000
GO:0036064	ciliary basal body	0.015	1.378	1.4×10^5	0.125	1.000
GO:0070062	extracellular exosome	0.104	0.570	2.1×10^6	0.125	1.000
GO:0090630	activation of GTPase activity	0.012	2.519	9.9×10^4	0.127	1.000
GO:0044212	transcription regulatory region DNA binding	0.004	0.322	2.7×10^5	0.128	1.000
GO:0055114	oxidation-reduction process	0.019	0.482	6.4×10^5	0.129	1.000
GO:0019886	antigen processing and presentation of exogenous...	0.012	1.393	9.6×10^4	0.129	1.000
GO:0005743	mitochondrial inner membrane	0.008	0.463	3.7×10^5	0.133	1.000
GO:0022617	extracellular matrix disassembly	0.012	1.009	9.6×10^4	0.135	1.000
GO:0042493	response to drug	0.004	0.043	2.6×10^5	0.137	1.000
GO:0008380	RNA splicing	0.008	0.098	2.6×10^5	0.139	1.000
GO:0043687	post-translational protein modification	0.008	0.319	3.6×10^5	0.140	1.000
GO:0010951	negative regulation of endopeptidase activity	0.012	2.037	9.9×10^4	0.140	1.000
GO:0000978	RNA polymerase II proximal promoter...	0.012	0.250	4.5×10^5	0.140	1.000
GO:0003713	transcription coactivator activity	0.004	0.339	2.6×10^5	0.142	1.000
GO:0001227	transcriptional repressor activity	0.012	1.384	9.9×10^4	0.144	1.000
GO:0005623	cell	0.012	1.927	1×10^5	0.150	1.000
GO:0030574	collagen catabolic process	0.012	1.036	9.9×10^4	0.151	1.000
GO:0006457	protein folding	0.015	2.465	1.5×10^5	0.158	1.000
GO:0016477	cell migration	0.004	0.121	2.4×10^5	0.159	1.000
GO:0070374	positive regulation of ERK1 and ERK2 cascade	0.019	4.238	2×10^5	0.160	1.000
GO:0007605	sensory perception of sound	0.015	0.824	1.5×10^5	0.162	1.000
GO:0031225	anchored component of membrane	0.012	0.892	1×10^5	0.171	1.000
GO:0016757	transferase activity	0.004	0.435	2.4×10^5	0.177	1.000
GO:0002376	immune system process	0.012	0.411	4.3×10^5	0.177	1.000
GO:0005759	mitochondrial matrix	0.008	0.361	3.3×10^5	0.185	1.000
GO:0010389	regulation of G2/M transition of mitotic cell...	0.012	0.924	1×10^5	0.188	1.000
GO:0005667	transcription factor complex	0.004	0.177	2.3×10^5	0.189	1.000
GO:0030054	cell junction	0.031	0.251	8.1×10^5	0.190	1.000
GO:0001650	fibrillar center	0.019	1.540	1.1×10^5	0.191	1.000
GO:0014069	postsynaptic density	0.004	0.172	2.2×10^5	0.200	1.000
GO:0046934	phosphatidylinositol-4	0.012	1.135	1.1×10^5	0.201	1.000
GO:0007267	cell-cell signaling	0.004	0.354	2.2×10^5	0.201	1.000
GO:0001649	osteoblast differentiation	0.012	1.917	1.1×10^5	0.201	1.000
GO:0045893	positive regulation of transcription	0.023	0.596	6.6×10^5	0.205	1.000
GO:0007399	nervous system development	0.015	0.450	5×10^5	0.205	1.000
GO:0001501	skeletal system development	0.015	1.212	1.6×10^5	0.207	1.000
GO:0019903	protein phosphatase binding	0.012	0.875	1.1×10^5	0.208	1.000
GO:1902476	chloride transmembrane transport	0.012	1.458	1.1×10^5	0.210	1.000
GO:0007568	aging	0.015	2.053	1.6×10^5	0.210	1.000

Table D: **Gene ontology enrichment in $\mathcal{B}_0$ SNPs.** GO terms that are enriched in genes with $\mathcal{B}_0$ SNPs in European humans (shown here are the first 100 rows with the lowest p_v). For each GO term, p is the proportion of $\mathcal{B}_0$ SNPs that are annotated with this focal GO term among all annotated $\mathcal{B}_0$ SNPs. For each gene, $p[\mathcal{B}_0]$ is the proportion of sites that have a $\mathcal{B}_0$ SNP (can be 0 if no $\mathcal{B}_0$ SNP is detected for this gene). For each GO term, r is the ratio of the mean of $p[\mathcal{B}_0]$ in the set of genes sharing the focal ontology over the mean of $p[\mathcal{B}_0]$ in the rest of the genes. Mann-Whitney U is the statistic testing if the distribution of $p[\mathcal{B}_0]$ is different between the two sets of genes (p-value p_v for the two-sided test). p_v^{adj} are corrected for multiple comparison (Holm–Bonferroni correction across the ontology terms). * for p_v^{adj} lower than the risk $\alpha = 0.05$.

2.2 Enrichment in $\mathcal{B}_0$ SNPs across all gene ontology terms

Whether SNPs classified as $\mathcal{B}_0$ are associated to a particular ontology is tested with a Mann-Whitney U statistic as described in Table D. This test is performed across across all 347 gene ontology terms, giving one p_{v} per ontology (Table D for the 100 lowest p_{v}). The distribution of p_{v} are not strongly associated to the ontology terms of their respective genes, as shown in the histogram of Figure C.

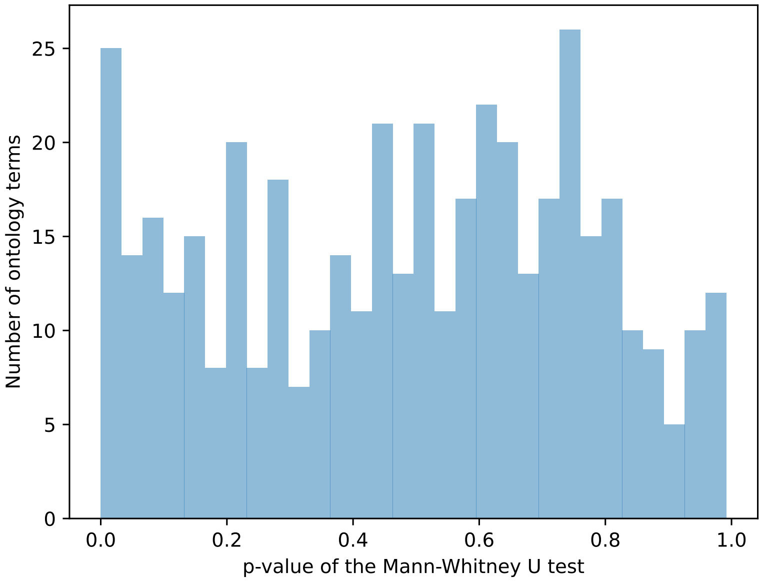

Figure C: **Enrichment in $\boldsymbol{\mathcal{B}_0}$ SNPs across all gene ontology terms**. In order to test if the distribution of $\mathcal{B}_0$ SNPs is different between genes sharing a common GO term and the rest of the genes, we used a Mann-Whitney U test. The distribution of p_{v} obtained across all 347 gene ontology terms is shown as a histogram.

3 Clinically related terms for mutations

3.1 Terms associated with deleterious mutations $\mathcal{D}_0$

SNPs predicted with $\mathcal{D}_0$ are statistically associated to clinical terms such as *Likely Pathogenic* and *Pathogenic*, as shown in Table E.

SNP clinical ontology	n_{Observed}	n_{Expected}	Odds ratio	p_{v}	$p_{\text{v-adjusted}}$
Benign	2969	4043.0	0.734	1.000	1.000
Likely benign	2994	3399.8	0.881	0.999	1.000
Risk factor	102	118.2	0.863	0.798	1.000
Likely pathogenic	221	68.5	3.226	1.7×10^{-8}	$\mathbf{6.7 \times 10^{-8}}$*
Pathogenic	560	193.6	2.893	4.2×10^{-17}	$\mathbf{2.1 \times 10^{-16}}$*

Table E: **Terms associated with deleterious mutations $\mathcal{D}_0$.** In humans (European population), non-synonymous SNPs in the test group ($\mathcal{D}_0$) are contrasted to SNPs in the control group ($\mathcal{N}_0$). For each clinical term, a 2x2 contingency table is built by counting the number of SNPs based on their selection coefficient and their clinical terms (whether they have this specific term or not). Fisher's exact tests are then performed for these 2x2 contingency tables. * for $p_{\text{v}}^{\text{adj}}$ corrected for multiple comparison (Holm–Bonferroni correction) lower than the risk $\alpha = 0.05$.

3.2 Terms associated with non-adaptive beneficial mutations $\mathcal{B}_0$

Beneficial non-adaptive mutations are associated with clinical terms such as *Benign* and *Likely Benign*, as shown in Table F.

SNP clinical ontology	n_{Observed}	n_{Expected}	Odds ratio	p_{v}	$p_{\text{v-adjusted}}$
Benign	319	261.7	1.219	0.002	**0.009***
Likely benign	263	222.7	1.181	0.012	**0.049***
Risk factor	5	7.847	0.637	0.879	0.879
Likely pathogenic	7	4.552	1.538	0.227	0.682
Pathogenic	16	12.9	1.241	0.268	0.682

Table F: **Terms associated with non-adaptive beneficial mutations $\mathcal{B}_0$.** In humans (European population), non-synonymous SNPs in the test group ($\mathcal{B}_0$) are contrasted to SNPs in the control group ($\mathcal{N}_0$). For each clinical term, a 2x2 contingency table is built by counting the number of SNPs based on their selection coefficient and their clinical terms (whether they have this specific term or not). Fisher's exact tests are then performed for these 2x2 contingency tables. * for $p_{\text{v}}^{\text{adj}}$ corrected for multiple comparison (Holm–Bonferroni correction) lower than the risk $\alpha = 0.05$.

References

1. Nielsen, R. & Yang, Z. Estimating the Distribution of Selection Coefficients from Phylogenetic Data with Applications to Mitochondrial and Viral DNA. *Molecular Biology and Evolution* **20,** 1231–1239 (2003).
