# Supplementary material for: Estimating the proportion of beneficial mutations that are not adaptive in mammals
Source: PLoS Genet. 2024 Dec 26;20(12):e1011536. doi: 10.1371/journal.pgen.1011536 (PMC11709321; doi:10.1371/journal.pgen.1011536)
Supplement: S3 File — Contains 7 pages of supplementary information including 8 figures (Fig A to H). (PDF) [file pgen.1011536.s003.pdf]

Estimating the proportion of beneficial mutations that are not adaptive in mammals

T. Latrille[1†], J. Joseph[2†], D. A. Hartasánchez[1], N. Salamin[1]

[1]Department of Computational Biology, Université de Lausanne, Lausanne, Switzerland

[2]Laboratoire de Biométrie et Biologie Evolutive, UMR5558, Université Lyon 1, Villeurbanne, France

[†]These authors contributed equally to this work

thibault.latrille@ens-lyon.org

S3 File

Contents

1 Contrasting selection at the phylogenetic and population-genetic scales **2**

1.1 Selection at the population-genetic scale . . . 2

1.2 Distribution of scaled selection coefficients (S_0) . . . 3

2 Correlation with diversity **5**

2.1 Phylogenetic Generalized Linear Model . . . 5

2.1.1 Proportion of deleterious mutations ($\mathcal{D}$) . . . 6

2.1.2 Proportion of nearly-neutral mutations ($\mathcal{N}$) . . . 6

2.1.3 Proportion of beneficial mutations ($\mathcal{B}$) . . . 7

List of Figures

A Estimation of selection at the population scale for $\mathcal{D}_0$ mutations. . . . 2

B Estimation of selection at the population scale for $\mathcal{N}_0$ mutations. . . . 2

C Estimation of selection at the population scale for $\mathcal{B}_0$ mutations. . . . 3

D Distribution of scaled selection coefficients (S_0). . . . 4

E Tree used for PGLM (Phylogenetic Generalized Linear Model). . . . 5

F Relationship between the proportion of deleterious mutations ($\mathcal{D}$) and effective population size. . . . 6

G Relationship between the proportion of nearly-neutral mutations ($\mathcal{N}$) and effective population size. . . . 7

H Relationship between the proportion of beneficial mutations ($\mathcal{B}$) and effective population size. . . . 7

1 Contrasting selection at the phylogenetic and population-genetic scales

1.1 Selection at the population-genetic scale

The proportion of deleterious ($\mathbb{P}[\mathcal{D}]$), nearly-neutral ($\mathbb{P}[\mathcal{N}]$) and of beneficial ($\mathbb{P}[\mathcal{B}]$) mutations estimated at the population-genetic scale across the different populations is shown for each class of selection: $\mathcal{D}_0$ (Figure A), $\mathcal{N}_0$ (Figure B) and $\mathcal{B}_0$ (Figure C).

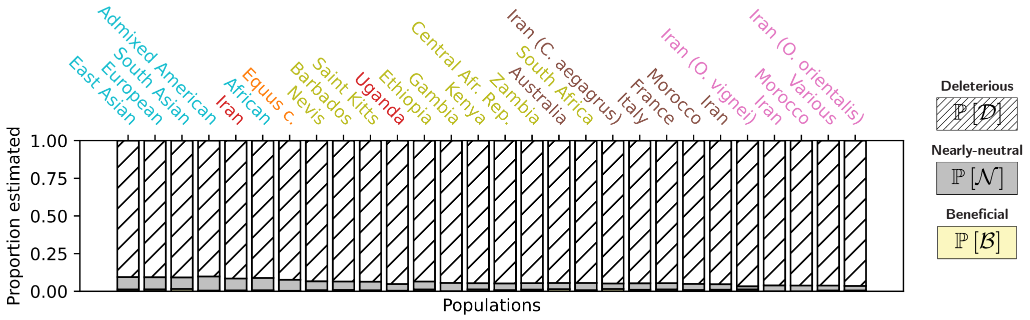

Figure A: **Estimation of selection at the population scale for $\mathcal{D}_0$ mutations.** Each stack bar corresponds to the DFE of new mutations predicted to be $\mathcal{D}_0$ by the mutation selection model, in a given population. The dashed area represents the proportion of deleterious mutations ($\mathcal{D}$), the gray area that of neutral mutations ($\mathcal{N}$), and the yellow area that of beneficial mutations ($\mathcal{B}$).

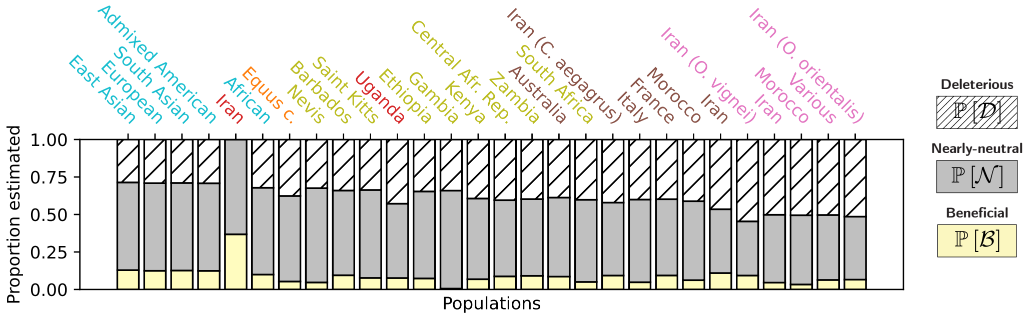

Figure B: **Estimation of selection at the population scale for $\mathcal{N}_0$ mutations.** Each stack bar corresponds to the DFE of new mutations predicted to be $\mathcal{N}_0$ by the mutation selection model, in a given population. The dashed area represents the proportion of deleterious mutations ($\mathcal{D}$), the gray area that of neutral mutations ($\mathcal{N}$), and the yellow area that of beneficial mutations ($\mathcal{B}$).

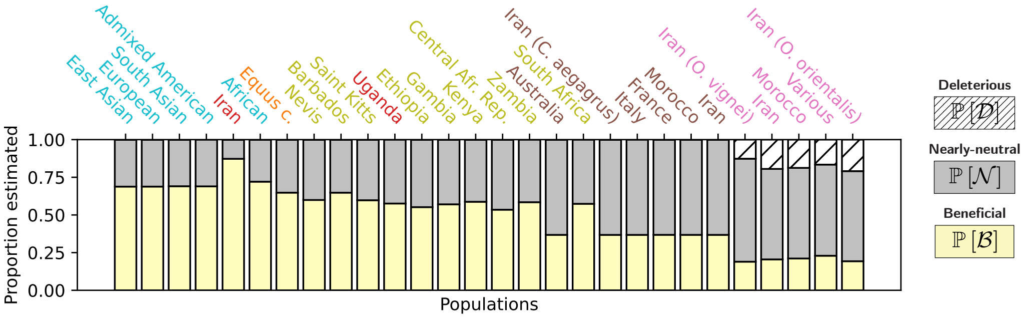

Figure C: **Estimation of selection at the population scale for $\mathcal{B}_0$ mutations.** Each stack bar corresponds to the DFE of new mutations predicted to be $\mathcal{B}_0$ by the mutation selection model, in a given population. The dashed area represents the proportion of deleterious mutations ($\mathcal{D}$), the gray area that of neutral mutations ($\mathcal{N}$), and the yellow area that of beneficial mutations ($\mathcal{B}$).

1.2 Distribution of scaled selection coefficients (S_0)

Theoretically, the selection coefficient (S_0) for any mutation is related to its probability of fixation by the equation $\mathbb{P}_{\text{fix}}(S_0) = S_0/(1-\exp(-S_0))$. It is thus possible to predict the distribution of S_0 of substitutions given the distribution of S_0 for mutations (Figure DA for one population of *Chlorocebus sabaeus*) by weighting the different mutations by their $\mathbb{P}_{\text{fix}}(S_0)$. The predicted DFE (distribution of fitness effects) of substitutions (Figure DB) matches the observed DFE of substitutions (Figure DC) in the terminal lineage, meaning that the S_0 obtained by phylogenetic mutation-selection codon models is a strong predictor of selection effectively exerted on mutations in terminal lineages. Equivalent figures to Figure D for each one of the 28 populations are available at Zenodo (`https://doi.org/10.5281/zenodo.7878953`) in file *distribution_S0_28_populations.pdf*.

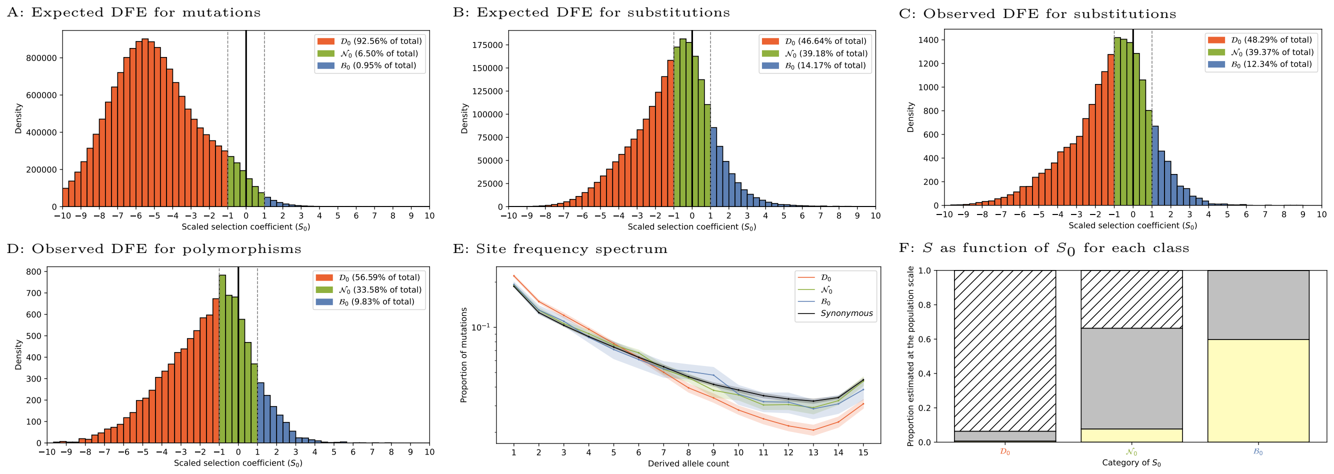

Figure D: **Distribution of scaled selection coefficients (S_0) in the Saint Kitts population of *Chlorocebus sabaeus.*** A) Distribution of scaled selection coefficients (S_0), predicted for all possible non-synonymous DNA mutations away from the ancestral exome. Mutations are divided into three classes of selection: deleterious ($\mathcal{D}_0$), nearly-neutral ($\mathcal{N}_0$) and beneficial non-adaptive ($\mathcal{B}_0$). B) Expected distribution of S_0 for substitutions obtained by transforming the distribution of S_0 for mutations (panel A) by weighting each mutation by its scaled probability of fixation given by $\mathbb{P}_{\text{fix}}(S_0) = S_0/(1 - \exp(-S_0))$. C) Distribution of scaled selection coefficients (S_0) for all observed substitutions along the terminal branch of the phylogenetic tree. D) Distribution of scaled selection coefficients (S_0) for all observed polymorphisms currently segregating in the population. E) The site-frequency spectrum (SFS) in the population for a random sample of 16 alleles (means in solid lines and standard deviations in color shades) for each class of selection and for synonymous mutations, supposedly neutral (black). The SFS represents the proportion of mutations (y-axis) with a given number of derived alleles in the population (x-axis). At high frequencies, deleterious mutations are underrepresented. F) Proportion of beneficial $\mathbb{P}[\mathcal{D}]$, nearly-neutral $\mathbb{P}[\mathcal{N}]$, and deleterious mutations $\mathbb{P}[\mathcal{B}]$ estimated at the population scale for each class of selection at the phylogenetic scale. Proportions depicted here are not weighted by their mutational opportunities.

2 Correlation with diversity

2.1 Phylogenetic Generalized Linear Model

Because a correlation must account for phylogenetic relationship and non-independence of samples, we fitted a Phylogenetic Generalized Linear Model (PGLM) in R with the package caper [1], with multi-furcation of the different populations inside each species. The mammalian tree imported from TimeTree [2] and pruned to the species used in this study (Figure E) is shown in newick format:

```
((Equus:76,((IRBT:3.55912,UGBT:3.55912)1993:18.0665,((IROA:5.33074,IROO:5.33074,ISGC
    :5.33074,IROV:5.33074,MOOA:5.33074)2219:2.32334,(IRCA:3.96087,IRCH:3.96087,AUCH
    :3.96087,FRCH:3.96087,ITCH:3.96087,MOCH:3.96087)2201:3.69321)2218:13.9716)
    2262:54.3744)2405:18,((AFR:5.59333,AMR:5.59333,EAS:5.59333,EUR:5.59333,SAS
    :5.59333)8454:22.42,(Barbados:2.69204,CAR:2.69204,Ethiopia:2.69204,Gambia:2.69204,
    Kenya:2.69204,Nevis:2.69204,SK:2.69204,SA:2.69204,Zambia:2.69204)8449:26.128)
    8800:65.18);
```

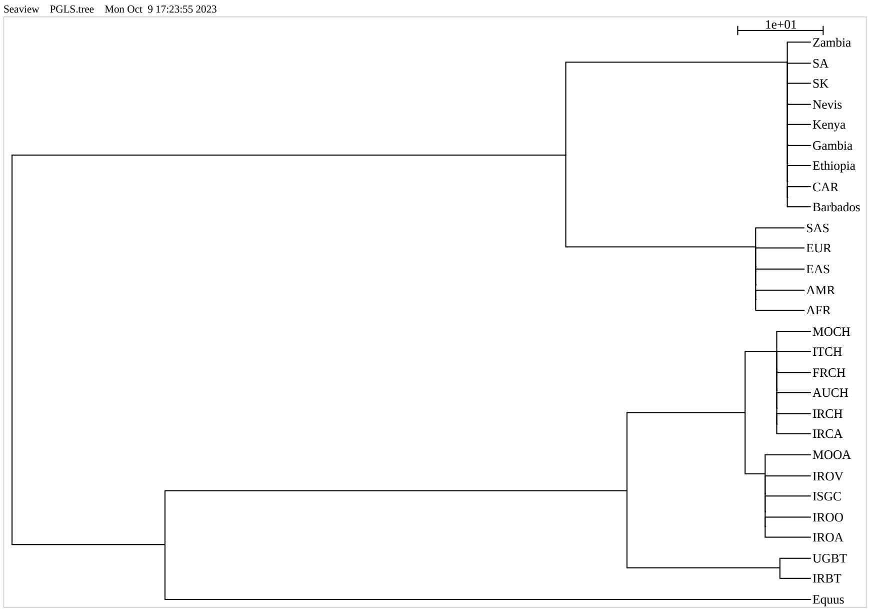

Figure E: **Tree used for PGLM (Phylogenetic Generalized Linear Model).** The mammalian dated tree was obtained from TimeTree [2] and pruned to include only the species analysed in this study, with multi-furcation of the different populations from each species placed at the same divergence time as the divergence to the sister species.

Accounting for phylogenetic relationship and non-independence of samples, through the fit of a Phylogenetic Generalized Linear Model, we can estimate regression coefficient between two variables. Thus, for each population, the proportion of deleterious ($\mathbb{P}[\mathcal{D}]$, F), nearly-neutral ($\mathbb{P}[\mathcal{N}]$, G) and beneficial ($\mathbb{P}[\mathcal{B}]$, H) mutations estimated at the population-genetic scale is shown as function of N_{e}, and we estimated regression coefficients.

2.1.1 Proportion of deleterious mutations ($\mathcal{D}$)

Higher effective population size (N_{e}) is typically accompanied by a higher proportion of effectively deleterious mutations at the population scale ($\mathbb{P}[\mathcal{D}]$), as shown in F (left panel). This trend is also confirmed when we restricted the analysis to class of mutations that are supposedly deleterious at the phylogenetic scale ($\mathcal{D}_0$), as shown in F (right panel). This result is qualitatively in accordance with the nearly-neutral theory of evolution which argues that very slightly deleterious mutations are more efficiently purified in large populations.

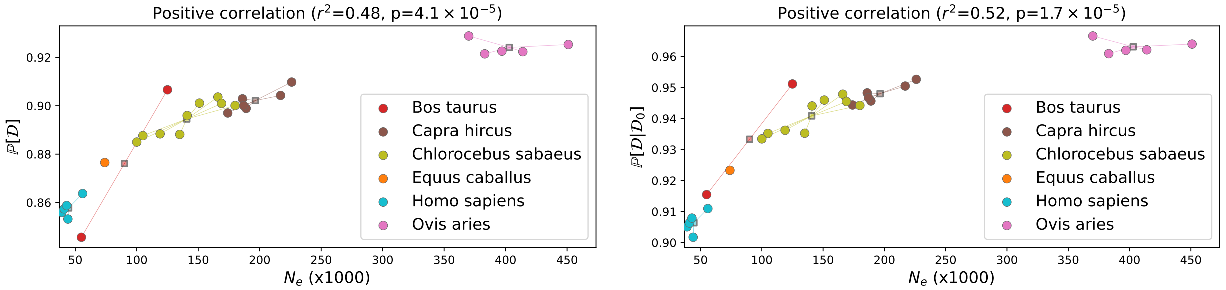

Figure F: **Relationship between the proportion of deleterious mutations ($\mathcal{D}$) and effective population size.** Left) Proportion of deleterious mutations at the population scale ($\mathbb{P}[\mathcal{N}]$), shown as a function of estimated effective population size (N_{e}). Right) Probability for a mutation to be deleterious at the population scale, given it is predicted to be deleterious at the phylogenetic scale, as a function of estimated effective population size (N_{e}). Populations are represented by circles, and squares are the mean for the species. Correlations account for phylogenetic relationship and non-independence of samples, through the fit of a Phylogenetic Generalized Linear Model (see Materials & Methods).

2.1.2 Proportion of nearly-neutral mutations ($\mathcal{N}$)

Higher effective population size (N_{e}) is typically accompanied by a smaller proportion of neutral mutations at the population scale ($\mathbb{P}[\mathcal{N}]$ in the range 0.06-0.18), as shown in G (left panel). This result is more pronounced ($\mathbb{P}[\mathcal{N} \mid \mathcal{N}_0]$ in the range 0.36-0.73) when we restricted the analysis to the class of mutations that are supposedly nearly-neutral at the phylogenetic scale ($\mathcal{N}_0$), as shown in H (right panel). This result suggests that in populations with higher diversity (e.g., *Bos* or *Ovis*), mutations are more likely to be detected as under selection (positive or negative). Alternatively stated, mutations in populations with low diversity (e.g., *Homo*) are effectively nearly-neutral and behave as neutral mutations would. This result is qualitatively in accordance with the nearly-neutral theory of evolution which argues that mutations are less efficiently selected for in small populations.

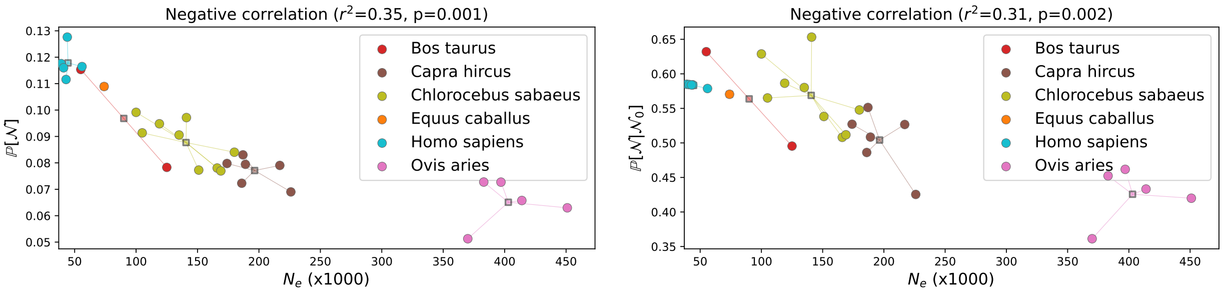

Figure G: **Relationship between the proportion of nearly-neutral mutations ($\mathcal{N}$) and effective population size.** Left) Proportion of deleterious mutations at the population scale ($\mathbb{P}[\mathcal{N}]$), shown as a function of estimated effective population size (N_e). Right) Probability for a mutation to be nearly-neutral at the population scale, given it is predicted to be nearly-neutral at the phylogenetic scale, as a function of estimated effective population size (N_e). Populations are represented by circles, and squares are the mean for the species. Correlations account for phylogenetic relationship and non-independence of samples, through the fit of a Phylogenetic Generalized Linear Model (see Materials & Methods).

2.1.3 Proportion of beneficial mutations ($\mathcal{B}$)

A higher effective population size (N_e) is accompanied by a smaller proportion of beneficial mutations at the population scale ($\mathbb{P}[\mathcal{B}]$), as shown in H (left panel). This trend is also confirmed when we restricted the analysis to the class of mutations that are supposedly non-adaptive beneficial at the phylogenetic scale ($\mathcal{B}_0$), as shown in H (right panel).

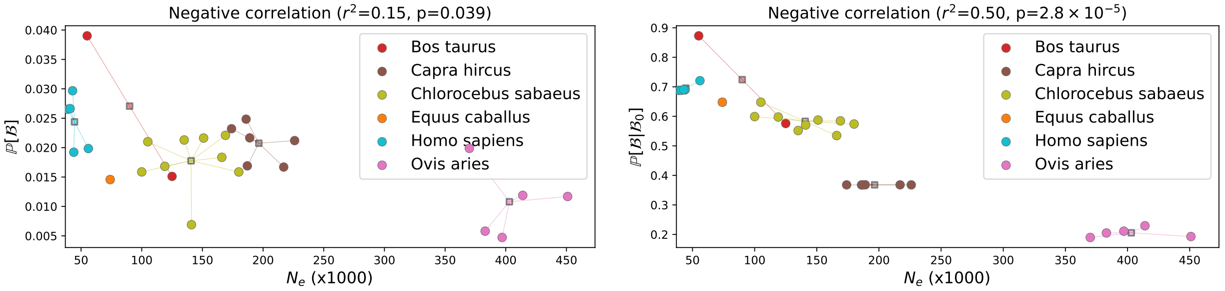

Figure H: **Relationship between the proportion of beneficial mutations ($\mathcal{B}$) and effective population size.** Left) Proportion of beneficial mutations at the population scale ($\mathbb{P}[\mathcal{N}]$), shown as a function of estimated effective population size (N_e). Right) Probability for a mutation to be beneficial at the population scale, given it is predicted to be beneficial at the phylogenetic scale, as a function of estimated effective population size (N_e). Populations are represented by circles, and squares are the mean for the species. Correlations account for phylogenetic relationship and non-independence of samples, through the fit of a Phylogenetic Generalized Linear Model (see Materials & Methods).

References

1. Orme, D. *et al.* The Caper Package: Comparative Analysis of Phylogenetics and Evolution in R. *R package version* **5,** 1–36 (2013).

2. Kumar, S., Stecher, G., Suleski, M. & Hedges, S. B. TimeTree: A Resource for Timelines, Timetrees, and Divergence Times. *Molecular Biology and Evolution* **34,** 1812–1819 (2017).
