# Supplementary material for: Estimating the proportion of beneficial mutations that are not adaptive in mammals
Source: PLoS Genet. 2024 Dec 26;20(12):e1011536. doi: 10.1371/journal.pgen.1011536 (PMC11709321; doi:10.1371/journal.pgen.1011536)
Supplement: S4 File — Contains 21 pages of supplementary information including 9 figures (Fig A to I) and 9 tables (Table A to I). (PDF) [file pgen.1011536.s004.pdf]

Estimating the proportion of beneficial mutations that are not adaptive in mammals

T. Latrille[1†], J. Joseph[2†], D. A. Hartasánchez[1], N. Salamin[1]

[1]Department of Computational Biology, Université de Lausanne, Lausanne, Switzerland

[2]Laboratoire de Biométrie et Biologie Evolutive, UMR5558, Université Lyon 1, Villeurbanne, France

[†]These authors contributed equally to this work

thibault.latrille@ens-lyon.org

S4 File

Contents

1 Probability for beneficial mutations to be non-adaptive ($\mathbb{P}[\mathcal{B}_0 \mid \mathcal{B}]$) 3

1.1 Across different populations . . . 3

1.2 Misannotations of ancestral alleles . . . 4

1.3 Simulations of site-frequency spectra . . . 6

2 Filtering of variable sites 7

2.1 Masking CpG transitions . . . 7

2.2 Probability for beneficial mutations to be non-adaptive . . . 8

2.3 Precision and recall . . . 9

2.4 Example site-frequency spectrum . . . 10

3 Excluding genes under adaptation 11

4 Including substitutions in the terminal lineage to estimate S 12

4.1 Including substitutions in polyDFE . . . 12

4.2 Probability for beneficial mutations to be non-adaptive . . . 13

4.3 Precision and recall . . . 14

4.4 Misannotations of ancestral alleles . . . 15

4.5 Excluding genes under adaptation . . . 16

4.6 Fitting one or three distributions of fitness effects . . . 17

5 Non-parametric distribution of fitness effects 19

5.1 polyDFE model D (non-parametric) . . . 19

5.2 Probability for beneficial mutations to be non-adaptive . . . 19

5.3 Precision and recall . . . 21

List of Figures

A $\mathbb{P}[\mathcal{B}_0 \mid \mathcal{B}]$ as a function of distance to the sister species. 4
B Ratio of $\mathbb{P}[S_0]$ over $\mathbb{P}[-S_0]$ for polymorphisms of the African population of *Homo sapiens*. 5
C Proportion of non-adaptive mutations among beneficial ones ($\mathbb{P}[\mathcal{B}_0 \mid \mathcal{B}]$) estimated on synthetic site-frequency spectra. 7
D Site-frequency spectrum when masking CpG transitions. 10
E Including substitutions in the terminal lineage to estimate S 12
F $\mathbb{P}[\mathcal{B}_0 \mid \mathcal{B}]$ when including divergence to estimate S, as a function of distance to the sister species. 15
G $\mathbb{P}[\mathcal{D}]$ when fitting a single or three DFEs. 17
H $\mathbb{P}[\mathcal{N}]$ when fitting a single or three DFEs. 18
I $\mathbb{P}[\mathcal{B}]$ when fitting a single or three DFEs. 18

List of Tables

A Probability for beneficial mutations to be non-adaptive ($\mathbb{P}[\mathcal{B}_0 \mid \mathcal{B}]$) 3
B $\mathbb{P}[\mathcal{B}_0 \mid \mathcal{B}]$ when masking CpG transitions. 8
C Precision and recall when masking CpG transitions. 9
D $\mathbb{P}[\mathcal{B}_0 \mid \mathcal{B}]$ excluding or not genes under adaptation. 11
E $\mathbb{P}[\mathcal{B}_0 \mid \mathcal{B}]$ when including divergence to estimate S. 13
F Precision and recall when including divergence to estimate S. 14
G $\mathbb{P}[\mathcal{B}_0 \mid \mathcal{B}]$ excluding or not genes under adaptation, when including divergence to estimate S. 16
H $\mathbb{P}[\mathcal{B}_0 \mid \mathcal{B}]$ when using a discrete DFE to estimate S. 20
I Precision and recall when using a discrete DFE to estimate S. 21

1 Probability for beneficial mutations to be non-adaptive ($\mathbb{P}[\mathcal{B}_0 \mid \mathcal{B}]$)

1.1 Across different populations

Probability for beneficial mutations to be non-adaptive, namely $\mathbb{P}[\mathcal{B}_0 \mid \mathcal{B}]$, is obtained from Bayes' formula (eq. 14) merging three terms: the probability for a new mutation to be a non-adaptive beneficial mutation ($\mathbb{P}[\mathcal{B}_0]$, eq. 5), the probability for a mutation to be beneficial ($\mathbb{P}[\mathcal{B}]$, eq. 15) and the probability for a mutation to be beneficial at the population scale given that it is predicted to be a non-adaptive beneficial mutation at the phylogenetic scale ($\mathbb{P}[\mathcal{B} \mid \mathcal{B}_0]$, eq. 12). These four estimates across populations are shown in Table A.

Population	Species	N_e	$\mathbb{P}[\mathcal{B}_0]$	$\mathbb{P}[\mathcal{B}]$	$\frac{\mathbb{P}[\mathcal{B}_0]}{\mathbb{P}[\mathcal{B}]}$	$\mathbb{P}[\mathcal{B} \mid \mathcal{B}_0]$	$\mathbb{P}[\mathcal{B}_0 \mid \mathcal{B}]$
Equus c.	Equus caballus	7.5×10^4	0.012	0.015	0.827	0.648	0.536
Iran	Bos taurus	5.6×10^4	0.011	0.039	0.279	0.873	0.243
Uganda	Bos taurus	1.3×10^5	0.011	0.015	0.720	0.576	0.415
Australia	Capra hircus	1.7×10^5	0.011	0.023	0.480	0.368	0.177
France	Capra hircus	1.9×10^5	0.011	0.022	0.515	0.368	0.190
Iran (C. aegagrus)	Capra hircus	1.9×10^5	0.011	0.025	0.448	0.368	0.165
Iran	Capra hircus	2.3×10^5	0.011	0.021	0.525	0.368	0.193
Italy	Capra hircus	1.9×10^5	0.011	0.017	0.660	0.368	0.243
Morocco	Capra hircus	2.2×10^5	0.011	0.017	0.667	0.368	0.245
Iran	Ovis aries	3.8×10^5	0.012	0.006	1.984	0.205	0.407
Iran (O. orientalis)	Ovis aries	4.5×10^5	0.011	0.012	0.983	0.193	0.190
Iran (O. vignei)	Ovis aries	3.7×10^5	0.012	0.020	0.579	0.190	0.110
Various	Ovis aries	4.1×10^5	0.011	0.012	0.967	0.229	0.222
Morocco	Ovis aries	4×10^5	0.012	0.005	2.435	0.211	0.514
Barbados	Chlorocebus sabaeus	1.1×10^5	0.009	0.021	0.452	0.648	0.293
Central Afr. Rep.	Chlorocebus sabaeus	1.7×10^5	0.009	0.018	0.515	0.535	0.275
Ethiopia	Chlorocebus sabaeus	1.4×10^5	0.009	0.021	0.444	0.552	0.245
Gambia	Chlorocebus sabaeus	1.4×10^5	0.009	0.007	1.423	0.577	0.821
Kenya	Chlorocebus sabaeus	1.5×10^5	0.009	0.022	0.437	0.588	0.257
Nevis	Chlorocebus sabaeus	1×10^5	0.009	0.016	0.597	0.599	0.358
South Africa	Chlorocebus sabaeus	1.8×10^5	0.009	0.016	0.594	0.574	0.341
Saint Kitts	Chlorocebus sabaeus	1.2×10^5	0.009	0.017	0.563	0.598	0.336
Zambia	Chlorocebus sabaeus	1.7×10^5	0.009	0.022	0.427	0.585	0.250
African	Homo sapiens	5.6×10^4	0.010	0.020	0.484	0.721	0.349
Admixed American	Homo sapiens	4.5×10^4	0.010	0.019	0.500	0.690	0.345
East Asian	Homo sapiens	4×10^4	0.010	0.027	0.362	0.688	0.249
European	Homo sapiens	4.2×10^4	0.010	0.027	0.361	0.688	0.248
South Asian	Homo sapiens	4.4×10^4	0.010	0.030	0.324	0.691	0.224

Table A: **Probability for beneficial mutations to be non-adaptive ($\mathbb{P}[\mathcal{B}_0 \mid \mathcal{B}]$).** N_{e} is the estimated effective population size. $\mathbb{P}[\mathcal{B}_0]$ (eq. 5) is the probability for a new mutation to be a non-adaptive beneficial mutation. These mutations have a selection coefficient predicted at the phylogenetic-scale larger than 1, thus toward a more fit amino-acid. $\mathbb{P}[\mathcal{B}]$ (eq. 15) is the probability for a mutation to be beneficial. These mutations have a selection coefficient at the population-scale larger than 1. $\mathbb{P}[\mathcal{B} \mid \mathcal{B}_0]$ (eq. 12) is the probability for a mutation to be beneficial at the population scale, given that it is predicted to be a non-adaptive beneficial mutation at the phylogenetic scale (the *precision*). $\mathbb{P}[\mathcal{B}_0 \mid \mathcal{B}]$ (eq. 14) is the probability for a mutation to be non-adaptive given that it is beneficial at the population scale (the *recall*). This probability is obtained using Bayes' formula.

1.2 Misannotations of ancestral alleles

A potential caveat of our method is that if a deleterious derived allele segregates at low frequency but is wrongly annotated as ancestral, it will result in a SNP that will be both predicted as advantageous and segregating at high frequency, wrongly suggesting that this variant is correctly inferred as beneficial. In this context, misannotations of ancestral alleles could inflate our estimation of the proportion of beneficial non-adaptive mutations. However, we show that this problem impacts our estimation in two ways, albeit, only slightly.

First, if misannotation is influencing the recall for $\mathcal{B}_0$ ($\mathbb{P}[\mathcal{B}_0 \mid \mathcal{B}]$), then the recall should increase as a function of divergence to the sister species (d_S: number of synonymous substitutions per site) since divergence would increase the rate of misannotations. Shown in Figure A is recall $\mathbb{P}[\mathcal{B}_0 \mid \mathcal{B}]$ as a function of divergence (d_S) in which the latter is not an explanatory variable.

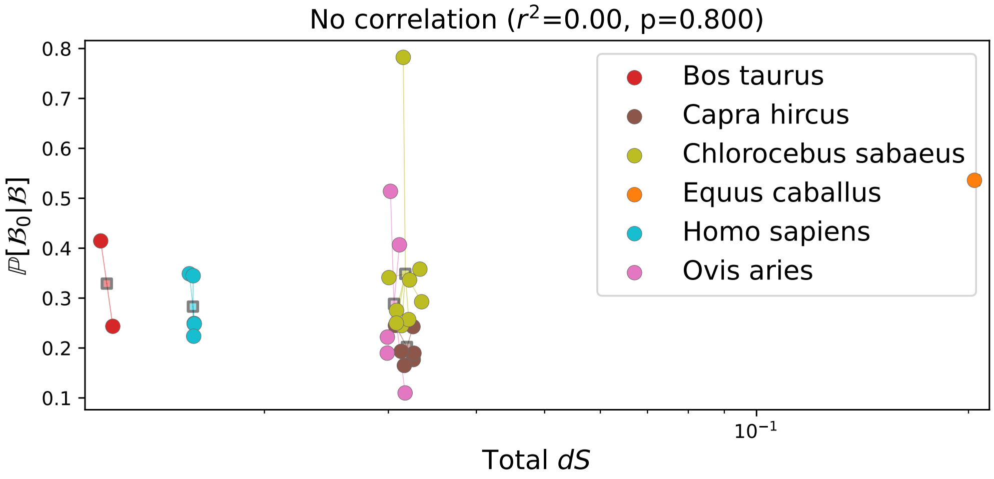

Figure A: **Proportion of non-adaptive mutations among beneficial ones ($\mathbb{P}[\mathcal{B}_0 \mid \mathcal{B}]$) as a function of distance to the sister species.** $\mathbb{P}[\mathcal{B}_0 \mid \mathcal{B}]$ (eq. 14) is the probability for a beneficial mutation to be non-adaptive. d_S is number of synonymous substitutions per site that occurred since divergence with the sister species (closest species in the mammalian tree). Populations are represented by circles, and squares are the mean for the species. Correlations account for phylogenetic relationship and non-independence of samples, through the fit of a Phylogenetic Generalized Linear Model (see Materials & Methods).

Second, the distribution of selection coefficients (S_0) that we observed for segregating mutations and for divergence also suggests that we effectively controlled for misannotations. Under a scenario where beneficial non-adaptive mutations would only be the result of misannotations, one would expect that the positive part of the fitness effects ($\mathbb{P}[S_0]$) of observed polymorphisms currently segregating in the population should reflect the negative one ($\mathbb{P}[-S_0]$). Indeed, the number of misannotated beneficial mutations should be proportional to the number of deleterious mutations with the same selection coefficient. The ratio between advantageous and deleterious polymorphisms of the same selection coefficient should therefore be a constant that represents the rate of misannotation (typically influenced by the distance to the sister species). However, this is not what we observe in the DFE of polymorphisms (shown in Figure B for the African population of *Homo sapiens*) thus giving us the confidence that misannotations are not common. Overall, the observed DFE of substitutions is much better explained by the expected DFE of new mutations (section S**??** than by misannotations).

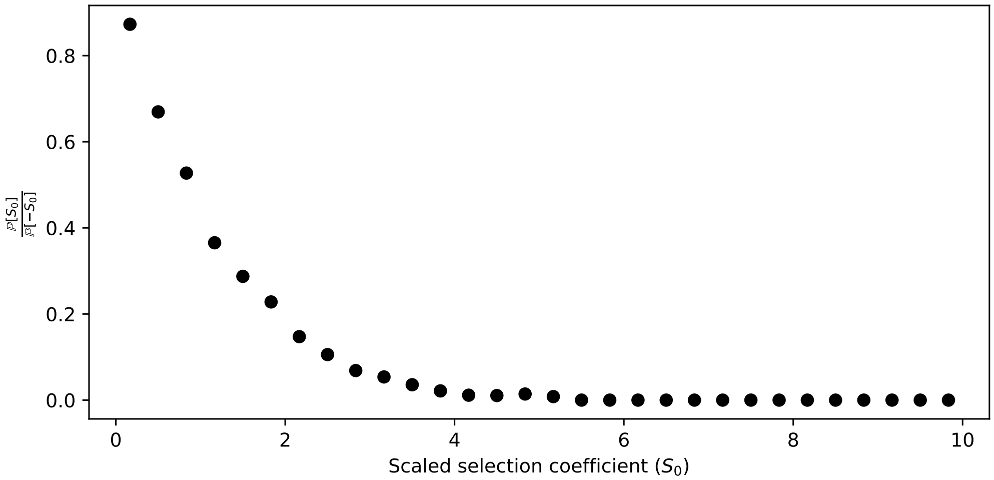

Figure B: **Ratio of $\mathbb{P}[S_0]$ over $\mathbb{P}[-S_0]$ for polymorphisms of the African population of *Homo sapiens*.** From the histogram of distribution of scaled selection coefficients (S_0) for all observed polymorphism, we obtain 30 bins for positive selection coefficient (S_0) and 30 bins for negative selection coefficient (S_0). For a given bin of S_0, $\mathbb{P}[S_0]$ the number of observed polymorphisms in the positive part, while $\mathbb{P}[-S_0]$ the number of observed polymorphisms in the negative part. $\mathbb{P}[S_0]/\mathbb{P}[-S_0]$ is the ratio of the positive part over the negative part. Alternatively stated, it is folding the DFE of observed polymorphism (Panel D of Fig D in S3 File) around 0, by taking the right part (positive S_0) of the histogram over the left part (negative S_0).

1.3 Simulations of site-frequency spectra

We performed population-genetic simulations to test whether our approach is able to recover the proportion of beneficial mutations that are non-adaptive in synthetic polymorphism datasets. More specifically, we performed simulations of site-frequency spectra, thus at the population-genetic scale, by using a mixture of the DFEs that is predicted from the phylogenetic mutation-selection model, and adding adaptive mutations to this DFE. In practice, simulations are performed under the Poisson Random Field (PRF) framework of population genetics[1]. Each simulation generates a synthetic synonymous SFS, and a synthetic non-synonymous SFS for the mutations in the category $x \in \{\mathcal{D}_0, \mathcal{N}_0, \mathcal{B}_0\}$.

Technically, for non-synonymous mutations, we first draw a random sample of the selection coefficients from the mixture of DFEs using $\mathbb{P}[y \mid x]$ for $y \in \{\mathcal{D}, \mathcal{N}, \mathcal{B}\}$ as parameters. Importantly, $\mathbb{P}[\mathcal{B} \mid \mathcal{D}_0]$ and $\mathbb{P}[\mathcal{B} \mid \mathcal{N}_0]$ are considered adaptive mutations, and thus their selection coefficients have a selection coefficient of $S_{\text{adaptive}} = 10$. All the others are drawn for the DFEs that are predicted from the phylogenetic mutation-selection model. Synonymous mutations are considered neutral and have $S = 0$.

Given these selection coefficients, to obtain the expected SFS we integrate over the limiting density of frequency that mutations are segregating in the population, as described from eqs. 1–2 in Tataru *et al.* [2]. And finally, given this expected SFS, we perform a Poisson random sampling to obtain a sampled SFS of size n matching the total number of polymorphism to simulate.

In summary, the simulations generates a synonymous SFS, and a non-synonymous SFS for the mutations in the category $x \in \{\mathcal{D}_0, \mathcal{N}_0, \mathcal{B}_0\}$, and require as input the following:

- Total number of polymorphisms to simulate (synonymous and non-synonymous).
- The DFE of S_0 predicted from the phylogenetic mutation-selection.
- Estimates of $\mathbb{P}[y \mid x]$ for $y \in \{\mathcal{D}, \mathcal{N}, \mathcal{B}\}$.
- Selection coefficient for adaptive mutations, here $S_{\text{adaptive}} = 20$.
- The sampled SFS size n, here $n = 16$ (i.e. twice the number of sampled individuals).

From these two SFS (synonymous and non-synonymous), we run polyDFE to obtain $\mathbb{P}[\mathcal{D} \mid x]$, $\mathbb{P}[\mathcal{N} \mid x]$ and $\mathbb{P}[\mathcal{B} \mid x]$ (eqs. 9–11). By doing so for the 3 categories $x \in \{\mathcal{D}_0, \mathcal{N}_0, \mathcal{B}_0\}$, we can ultimately compute $\mathbb{P}[\mathcal{B}_0 \mid \mathcal{B}]$ (eqs. 14–15), which can be then be compared to the ground truth.

We used 8 different sets of parameters ($\mathbb{P}[y \mid x]$ and number of polymorphisms) and simulated 10 replicates for each set of parameters, shown in Figure C. These set of input parameters are provided to match $\mathbb{P}[\mathcal{B}_0 \mid \mathcal{B}]$ of the following populations: Set 1 for Admixed American (*Homo sapiens*), Set 2 for African (*Homo sapiens*), Set 3 for Nevis (*Chlorocebus sabaeus*), Set 4 for Iran (*Ovis orientalis*), Set 5 for Uganda (*Bos taurus*), Set 6 for Morocco (*Ovis aries*), Set 7 for *Equus caballus*, Set 8 for Gambia (*Chlorocebus sabaeus*). For each synthetic data we ran polyDFE and derived $\mathbb{P}[\mathcal{B}_0 \mid \mathcal{B}]$ which was then compared to the ground truth (calculated analytically from the set parameters $\mathbb{P}[y \mid x], x \in \{\mathcal{D}_0, \mathcal{N}_0, \mathcal{B}_0\}, y \in \{\mathcal{D}, \mathcal{N}, \mathcal{B}\}$). These simulations show that we are able to accurately retrieve the proportion of non-adaptive mutations in those synthetic datasets.

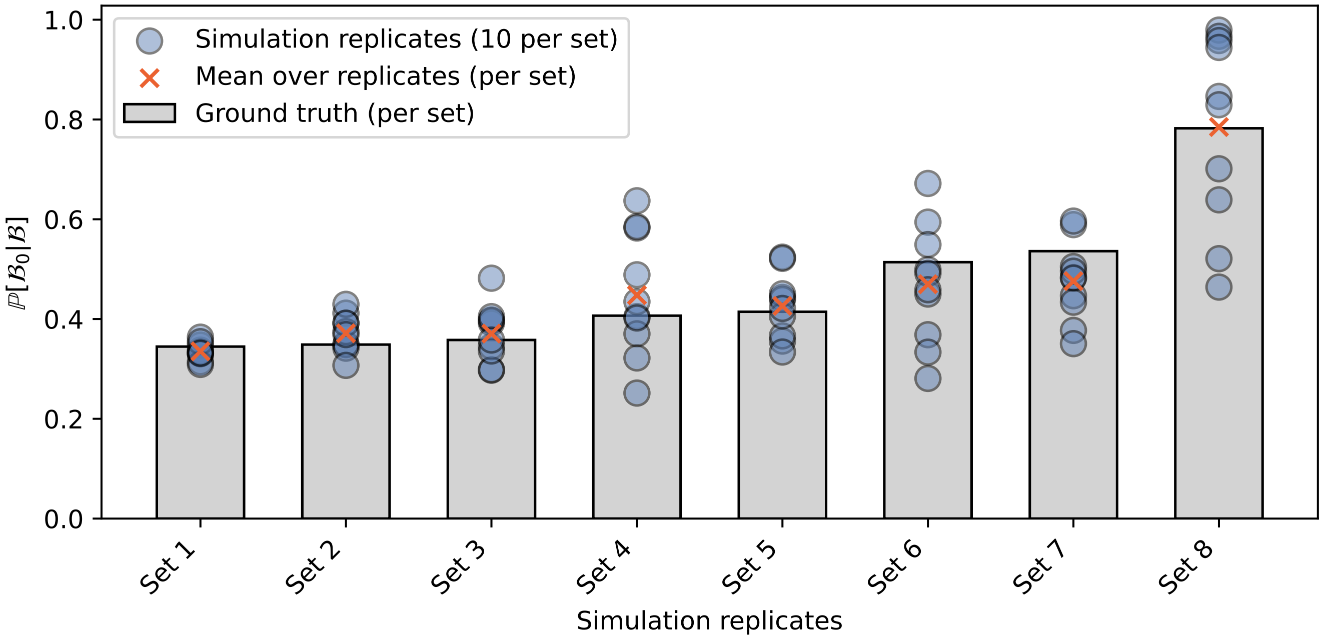

Figure C: **Proportion of non-adaptive mutations among beneficial ones ($\mathbb{P}[\mathcal{B}_0 \mid \mathcal{B}]$) estimated on synthetic site-frequency spectra.** Each set has ben simulated using a different value for $\mathbb{P}[\mathcal{B}_0 \mid \mathcal{B}]$. Bars correspond to the $\mathbb{P}[\mathcal{B}_0 \mid \mathcal{B}]$ used to simulate the site-frequency spectra, each blue circle corresponds to $\mathbb{P}[\mathcal{B}_0 \mid \mathcal{B}]$ estimated by one polyDFE simulation, and red crosses correspond to the mean of estimations across 10 replicates.

2 Filtering of variable sites

2.1 Masking CpG transitions

We also tested our prediction when masking CpG transitions since the mutation rate of CpG dinucleotides is higher than the mutation rate of other dinucleotides, which can affect the estimation of the DFE. Any nucleotide transition including a CpG dinucleotide either in the ancestral or derived state is considered as a CpG transition. We masked all CpG transitions in the mutational opportunities, the substitutions in the terminal branches, and the polymorphisms segregating in the population. We then re-estimated the DFE and the proportion of non-adaptive beneficial mutations.

2.2 Probability for beneficial mutations to be non-adaptive

When filtering out CpG sites, the estimation of $\mathbb{P}[\mathcal{B}]$ and $\mathbb{P}[\mathcal{B}_0 \mid \mathcal{B}]$ across populations is shown in Table B. Comparing these results with Table A, we acknowledge that the exact proportion of non-adaptive among beneficial mutations is dependent on the whether we masked CpG sites. However, we can still see that non-adaptive beneficial mutations are positively selected compared to neutral and deleterious ones, and this result is not dependent on whether we masked CpG sites or not.

Population	Species	N_e	$\mathbb{P}[\mathcal{B}_0]$	$\mathbb{P}[\mathcal{B}]$	$\frac{\mathbb{P}[\mathcal{B}_0]}{\mathbb{P}[\mathcal{B}]}$	$\mathbb{P}[\mathcal{B} \mid \mathcal{B}_0]$	$\mathbb{P}[\mathcal{B}_0 \mid \mathcal{B}]$
Equus c.	Equus caballus	7.5×10^4	0.012	0.028	0.421	0.543	0.229
Iran	Bos taurus	5.6×10^4	0.011	0.038	0.283	0.797	0.226
Uganda	Bos taurus	1.3×10^5	0.011	0.022	0.479	0.406	0.195
Australia	Capra hircus	1.7×10^5	0.011	0.024	0.454	0.158	0.072
France	Capra hircus	1.9×10^5	0.011	0.034	0.320	0.258	0.082
Iran (C. aegagrus)	Capra hircus	1.9×10^5	0.011	0.032	0.341	0.268	0.091
Iran	Capra hircus	2.3×10^5	0.011	0.026	0.417	0.294	0.123
Italy	Capra hircus	1.9×10^5	0.011	0.023	0.472	0.100	0.047
Morocco	Capra hircus	2.2×10^5	0.011	0.018	0.593	0.141	0.083
Iran	Ovis aries	3.8×10^5	0.011	0.012	0.981	0.243	0.239
Iran (O. orientalis)	Ovis aries	4.5×10^5	0.011	0.019	0.603	0.155	0.093
Iran (O. vignei)	Ovis aries	3.7×10^5	0.011	0.025	0.445	0.252	0.112
Various	Ovis aries	4.1×10^5	0.011	0.016	0.704	0.198	0.139
Morocco	Ovis aries	4×10^5	0.011	0.008	1.389	0.165	0.229
Barbados	Chlorocebus sabaeus	1.1×10^5	0.009	0.013	0.752	0.231	0.174
Central Afr. Rep.	Chlorocebus sabaeus	1.7×10^5	0.009	0.018	0.519	0.209	0.108
Ethiopia	Chlorocebus sabaeus	1.4×10^5	0.009	0.025	0.385	0.241	0.093
Gambia	Chlorocebus sabaeus	1.4×10^5	0.009	0.007	1.346	0.204	0.275
Kenya	Chlorocebus sabaeus	1.5×10^5	0.009	0.021	0.443	0.239	0.106
Nevis	Chlorocebus sabaeus	1×10^5	0.009	0.014	0.690	0.146	0.101
South Africa	Chlorocebus sabaeus	1.8×10^5	0.009	0.019	0.498	0.272	0.135
Saint Kitts	Chlorocebus sabaeus	1.2×10^5	0.009	0.017	0.567	0.217	0.123
Zambia	Chlorocebus sabaeus	1.7×10^5	0.009	0.024	0.388	0.308	0.119
African	Homo sapiens	5.6×10^4	0.010	0.026	0.367	0.411	0.151
Admixed American	Homo sapiens	4.5×10^4	0.010	0.017	0.551	0.374	0.206
East Asian	Homo sapiens	4×10^4	0.010	0.029	0.332	0.370	0.123
European	Homo sapiens	4.2×10^4	0.010	0.031	0.304	0.371	0.113
South Asian	Homo sapiens	4.4×10^4	0.010	0.032	0.302	0.369	0.111

Table B: **$\mathbb{P}[\mathcal{B}_0 \mid \mathcal{B}]$ when masking CpG transitions.** N_e is the estimated effective population size. $\mathbb{P}[\mathcal{B}_0]$ (eq. 5) is the probability for a new mutation to be a non-adaptive beneficial mutation. These mutations have a selection coefficient predicted at the phylogenetic-scale larger than 1, thus toward a more fit amino-acid. $\mathbb{P}[\mathcal{B}]$ (eq. 15) is the probability for a mutation to be beneficial. These mutations have a selection coefficient at the population-scale larger than 1. $\mathbb{P}[\mathcal{B} \mid \mathcal{B}_0]$ (eq. 12) is the probability for a mutation to be beneficial at the population scale, given it is predicted to be a non-adaptive beneficial mutation at the phylogenetic scale (the *precision*). $\mathbb{P}[\mathcal{B}_0 \mid \mathcal{B}]$ (eq. 14) is the probability for a mutation to be non-adaptive given it is beneficial at the population scale (the *recall*). This probability is obtained using Bayes' formula.

2.3 Precision and recall

The estimation of precision and recall across populations while filtering out CpG sites for the estimation of S is shown in Table C. Comparing these results with tables 1, we acknowledge that the exact quantification of *precision* and *recall* is dependent on the whether we masked CpG sites. However, we can still see that non-adaptive beneficial mutations are positively selected compared to neutral and deleterious ones, and this result is not dependent on whether we masked CpG sites or not.

			**Deleterious mutations** $\mathcal{D} := S < -1$ $\mathcal{D}_0 := S_0 < -1$		**Nearly-neutral mutations** $\mathcal{N} := -1 < S < 1$ $\mathcal{N}_0 := -1 < S_0 < 1$		**Beneficial mutations** $\mathcal{B} := S > 1$ $\mathcal{B}_0 := S_0 > 1$	
**Population**	**Species**	N_e	**Precision** $\mathbb{P}[\mathcal{D} \mid \mathcal{D}_0]$	**Recall** $\mathbb{P}[\mathcal{D}_0 \mid \mathcal{D}]$	**Precision** $\mathbb{P}[\mathcal{N} \mid \mathcal{N}_0]$	**Recall** $\mathbb{P}[\mathcal{N}_0 \mid \mathcal{N}]$	**Precision** $\mathbb{P}[\mathcal{B} \mid \mathcal{B}_0]$	**Recall** $\mathbb{P}[\mathcal{B}_0 \mid \mathcal{B}]$
Equus c.	Equus caballus	7.5×10^4	0.887	0.984	0.740	0.356	0.543	0.229
Iran	Bos taurus	5.6×10^4	0.888	1.000	0.632	0.302	0.797	0.226
Uganda	Bos taurus	1.3×10^5	0.929	0.973	0.501	0.349	0.406	0.195
Australia	Capra hircus	1.7×10^5	0.926	0.972	0.609	0.421	0.158	0.072
France	Capra hircus	1.9×10^5	0.927	0.959	0.354	0.321	0.258	0.082
Iran (C. aegagrus)	Capra hircus	1.9×10^5	0.931	0.967	0.502	0.423	0.268	0.091
Iran	Capra hircus	2.3×10^5	0.937	0.966	0.529	0.451	0.294	0.123
Italy	Capra hircus	1.9×10^5	0.927	0.970	0.550	0.390	0.100	0.047
Morocco	Capra hircus	2.2×10^5	0.933	0.965	0.534	0.401	0.141	0.083
Iran	Ovis aries	3.8×10^5	0.947	0.957	0.446	0.393	0.243	0.239
Iran (O. orientalis)	Ovis aries	4.5×10^5	0.951	0.961	0.396	0.386	0.155	0.093
Iran (O. vignei)	Ovis aries	3.7×10^5	0.954	0.960	0.379	0.432	0.252	0.112
Various	Ovis aries	4.1×10^5	0.949	0.956	0.434	0.418	0.198	0.139
Morocco	Ovis aries	4×10^5	0.946	0.959	0.453	0.374	0.165	0.229
Barbados	Chlorocebus sabaeus	1.1×10^5	0.916	0.971	0.523	0.306	0.231	0.174
Central Afr. Rep.	Chlorocebus sabaeus	1.7×10^5	0.933	0.969	0.486	0.353	0.209	0.108
Ethiopia	Chlorocebus sabaeus	1.4×10^5	0.918	0.973	0.533	0.348	0.241	0.093
Gambia	Chlorocebus sabaeus	1.4×10^5	0.925	0.971	0.572	0.343	0.204	0.275
Kenya	Chlorocebus sabaeus	1.5×10^5	0.931	0.971	0.521	0.378	0.239	0.106
Nevis	Chlorocebus sabaeus	1×10^5	0.916	0.972	0.535	0.313	0.146	0.101
South Africa	Chlorocebus sabaeus	1.8×10^5	0.928	0.970	0.546	0.378	0.272	0.135
Saint Kitts	Chlorocebus sabaeus	1.2×10^5	0.917	0.971	0.507	0.309	0.217	0.123
Zambia	Chlorocebus sabaeus	1.7×10^5	0.930	0.969	0.495	0.372	0.308	0.119
African	Homo sapiens	5.6×10^4	0.888	0.978	0.587	0.296	0.411	0.151
Admixed American	Homo sapiens	4.5×10^4	0.874	0.979	0.591	0.256	0.374	0.206
East Asian	Homo sapiens	4×10^4	0.877	0.980	0.593	0.280	0.370	0.123
European	Homo sapiens	4.2×10^4	0.885	0.980	0.591	0.301	0.371	0.113
South Asian	Homo sapiens	4.4×10^4	0.886	0.979	0.591	0.303	0.369	0.111

Table C: **Precision and recall when masking CpG transitions.** N_e is the estimated effective population size. *Precision* is the estimation of the selection coefficient at population scale (S) given that S_0 is known. *Recall* is the estimation of S_0 given selection coefficient at the population scale (S) is known. *Recall* for beneficial mutations ($\mathbb{P}[\mathcal{B}_0 \mid \mathcal{B}]$) is the probability for a mutation to be non-adaptive given it is beneficial at the population scale.

2.4 Example site-frequency spectrum

The site-frequency spectrum (SFS, panel A) and the congruency between fitness effects estimates (S versus S_0, panel B) are shown in Figure D for the African population of *Homo sapiens* and the South African population of *Chlorocebus sabaeus*.

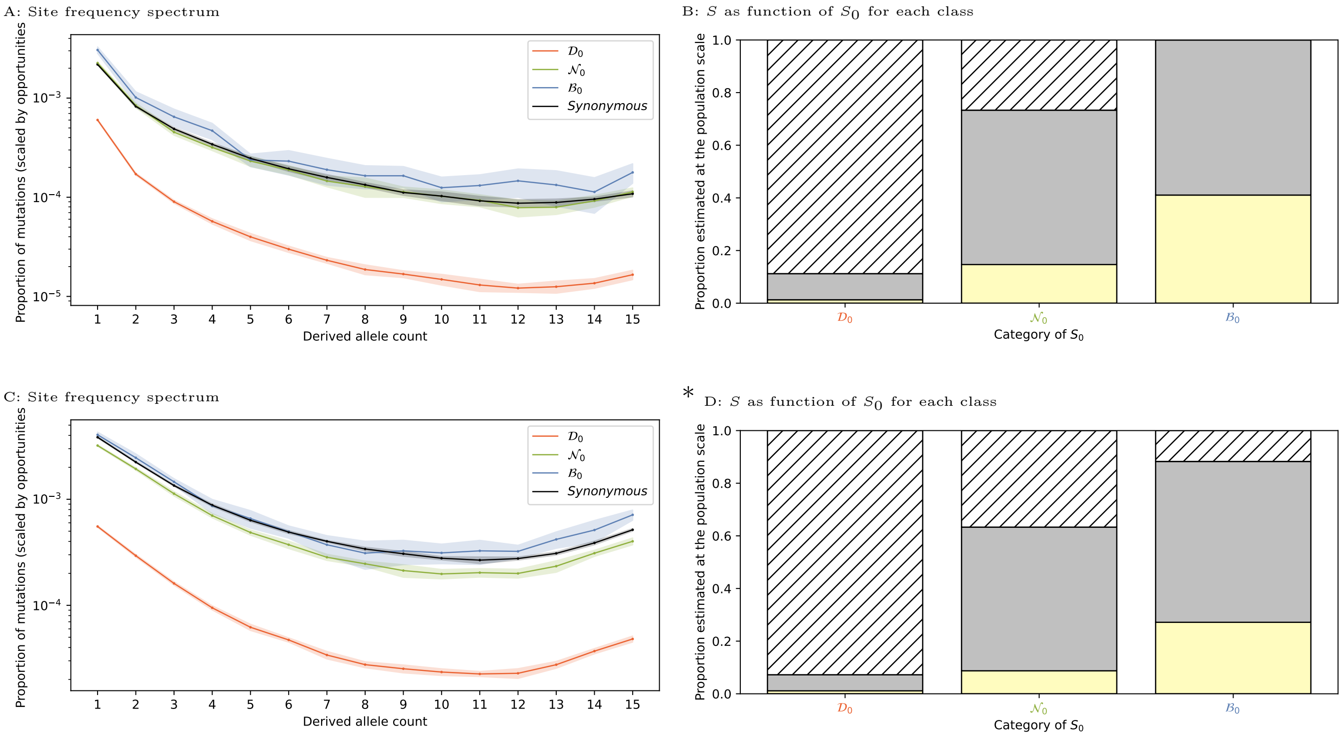

Figure D: **Site-frequency spectrum when masking CpG transitions.** Left (A&C): the site-frequency spectrum (SFS) in the population for a random sample of 16 alleles (means in solid lines and standard deviations in color shades) for each class of selection ($\mathcal{D}_0$, $\mathcal{N}_0$ and $\mathcal{B}_0$) and for synonymous mutations, supposedly neutral (black). The SFS represents the proportion of mutations (y-axis) with a given number of derived alleles in the population (x-axis). Here SFS are weighted by the mutational opportunities in each class of selection (synonymous, $\mathcal{D}_0$, $\mathcal{N}_0$ and $\mathcal{B}_0$). Right (B&D): proportion of beneficial $\mathbb{P}[\mathcal{D}]$, nearly-neutral $\mathbb{P}[\mathcal{N}]$, and deleterious mutations $\mathbb{P}[\mathcal{B}]$ estimated at the population scale for each class of selection at the phylogenetic scale. Proportions depicted here are not weighted by their mutational opportunities. Top (A&C): *Homo sapiens*, African population. Bottom (C&D): *Chlorocebus sabaeus*, South Africa population.

3 Excluding genes under adaptation

The proportion of beneficial non-adaptive mutations ($\mathbb{P}[\mathcal{B}_0 \mid \mathcal{B}]$) is higher when excluding genes under adaptation, as shown in Table G, consistent with our expectation that genes with uniformly conserved functions should fit better the nearly-neutral equilibrium model.

Population	Species	Control	Case
Equus c.	Equus caballus	0.536	0.880
Iran	Bos taurus	0.243	0.249
Uganda	Bos taurus	0.415	0.429
Australia	Capra hircus	0.177	0.190
France	Capra hircus	0.190	0.201
Iran (C. aegagrus)	Capra hircus	0.165	0.169
Iran	Capra hircus	0.193	0.176
Italy	Capra hircus	0.243	0.261
Morocco	Capra hircus	0.245	0.283
Iran	Ovis aries	0.407	0.454
Iran (O. orientalis)	Ovis aries	0.190	0.207
Iran (O. vignei)	Ovis aries	0.110	0.135
Various	Ovis aries	0.222	0.246
Morocco	Ovis aries	0.514	0.627
Barbados	Chlorocebus sabaeus	0.293	0.325
Central Afr. Rep.	Chlorocebus sabaeus	0.275	0.273
Ethiopia	Chlorocebus sabaeus	0.245	0.242
Gambia	Chlorocebus sabaeus	0.821	0.873
Kenya	Chlorocebus sabaeus	0.257	0.254
Nevis	Chlorocebus sabaeus	0.358	0.395
South Africa	Chlorocebus sabaeus	0.341	0.375
Saint Kitts	Chlorocebus sabaeus	0.336	0.355
Zambia	Chlorocebus sabaeus	0.250	0.263
African	Homo sapiens	0.349	0.363
Admixed American	Homo sapiens	0.345	0.312
East Asian	Homo sapiens	0.249	0.233
European	Homo sapiens	0.248	0.211
South Asian	Homo sapiens	0.224	0.218

Table D: $\mathbb{P}[\boldsymbol{\mathcal{B}_0} \mid \boldsymbol{\mathcal{B}}]$ **excluding or not genes under adaptation.** Genes under adaptation retrieved from Latrille *et al.* [3]. Comparison of $\mathbb{P}[\mathcal{B}_0 \mid \mathcal{B}]$ for the whole genome (control) and when excluding genes under adaptation (case). The non-parametric Wilcoxon signed-rank test evaluates the null hypothesis that the distribution of the differences (case versus control) is symmetric around zero. Applied to the paired samples (case and control), Wilcoxon signed-rank test results in $s = 80$ with p-value $= 0.002$, for the one-sided test (case higher than control).

4 Including substitutions in the terminal lineage to estimate S

4.1 Including substitutions in polyDFE

Divergence data (number of substitutions per site) can also be included into polyDFE to estimate the DFE, as shown in figure E. For the class of a given class of selection coefficient ($x \in \{\mathcal{D}_0, \mathcal{N}_0, \mathcal{B}_0\}$), the number of substitutions has already been computed and is given by $D(x)$ (eq. 6), and the number of sites is given by $L(x)$ (eq. 4). Otherwise, the procedure is the same as described section *Scaled selection coefficients (S) in a population-based method*.

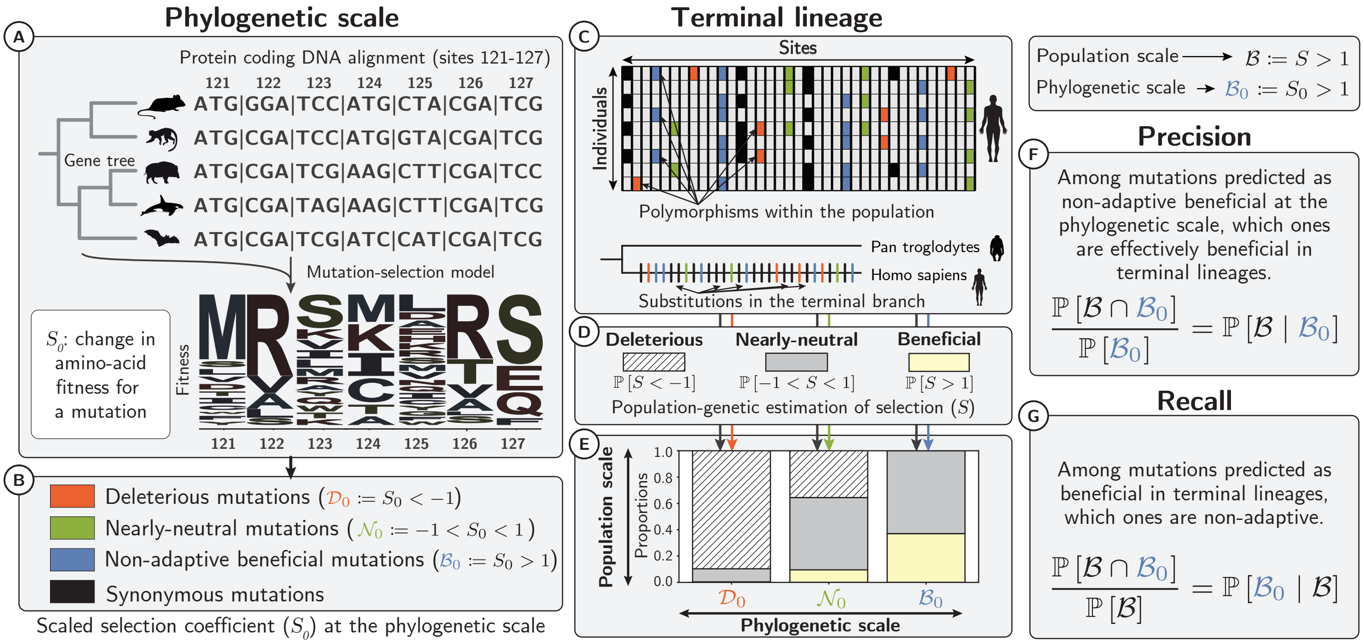

Figure E: **Including substitutions in the terminal lineage to estimate S.** Same as Figure 2 in the main text, but including substitutions in the terminal lineage to estimate S. At the phylogenetic scale (A), we estimated the amino-acid fitness for each site from protein-coding DNA alignments using mutation-selection codon models. For every possible mutation, the difference in amino-acid fitness before and after the mutation allows us to compute the selection coefficient at the phylogenetic scale (S_0). Depending on S_0 (B) mutations can be predicted as deleterious ($\mathcal{D}_0$), nearly-neutral ($\mathcal{N}_0$) or beneficial non-adaptive mutations ($\mathcal{B}_0$) toward a fitter amino acid and repairing existing functions. For each population, each observed single nucleotide polymorphism (SNP) segregating can also be classified according to its S_0 value (C). Within the terminal lineage leading to this population, each substitution can be classified according to its S value (C). Non-synonymous polymorphisms and divergence, contrasted to synonymous polymorphisms and divergence (deemed neutral) is used to estimate selection coefficients (D-E) at the population scale (S), for each class of selection ($\mathcal{D}_0$, $\mathcal{N}_0$, $\mathcal{B}_0$). We can thus assess whether S_0 predicts S and compute *precision* (F) and *recall* (G) for each class. The *recall* value for class $\mathcal{B}_0$ is the probability for beneficial mutations to be non-adaptive (G). Icons are adapted from https://phylopic.org under a Creative Commons license.

4.2 Probability for beneficial mutations to be non-adaptive

The estimation of $\mathbb{P}[\mathcal{B}]$ and $\mathbb{P}[\mathcal{B}_0 \mid \mathcal{B}]$ across populations when including substitutions in the terminal lineage and fitting a discrete DFE to S is shown in Table E. Altogether, comparing these results with table A, we acknowledge that the exact proportion of non-adaptive among all beneficial mutations is dependent whether we included or not substitutions in the terminal lineage for the estimation of S. However, we can still see that non-adaptive beneficial mutations are positively selected compared to neutral and deleterious ones, and this result is valid whether we included or not substitutions in the terminal lineage for the estimation of S.

Population	Species	N_e	$\mathbb{P}[\mathcal{B}_0]$	$\mathbb{P}[\mathcal{B}]$	$\frac{\mathbb{P}[\mathcal{B}_0]}{\mathbb{P}[\mathcal{B}]}$	$\mathbb{P}[\mathcal{B} \mid \mathcal{B}_0]$	$\mathbb{P}[\mathcal{B}_0 \mid \mathcal{B}]$
Equus c.	Equus caballus	7.5×10^4	0.012	0.057	0.213	0.498	0.106
Iran	Bos taurus	5.6×10^4	0.011	0.006	1.860	0.368	0.684
Uganda	Bos taurus	1.3×10^5	0.011	0.012	0.900	0.307	0.276
Australia	Capra hircus	1.7×10^5	0.011	0.007	1.651	0.178	0.294
France	Capra hircus	1.9×10^5	0.011	0.007	1.614	0.183	0.295
Iran (C. aegagrus)	Capra hircus	1.9×10^5	0.011	0.012	0.923	0.177	0.163
Iran	Capra hircus	2.3×10^5	0.011	0.008	1.483	0.184	0.273
Italy	Capra hircus	1.9×10^5	0.011	0.007	1.686	0.178	0.299
Morocco	Capra hircus	2.2×10^5	0.011	0.006	1.892	0.184	0.349
Iran	Ovis aries	3.8×10^5	0.012	0.010	1.132	0.189	0.214
Iran (O. orientalis)	Ovis aries	4.5×10^5	0.011	0.011	1.012	0.213	0.216
Iran (O. vignei)	Ovis aries	3.7×10^5	0.012	0.021	0.561	0.186	0.105
Various	Ovis aries	4.1×10^5	0.011	0.011	1.069	0.215	0.229
Morocco	Ovis aries	4×10^5	0.012	0.012	0.973	0.217	0.211
Barbados	Chlorocebus sabaeus	1.1×10^5	0.009	0.004	2.120	0.341	0.723
Central Afr. Rep.	Chlorocebus sabaeus	1.7×10^5	0.009	0.013	0.720	0.368	0.265
Ethiopia	Chlorocebus sabaeus	1.4×10^5	0.009	0.007	1.352	0.368	0.498
Gambia	Chlorocebus sabaeus	1.4×10^5	0.009	0.010	0.956	0.368	0.352
Kenya	Chlorocebus sabaeus	1.5×10^5	0.009	0.013	0.727	0.368	0.268
Nevis	Chlorocebus sabaeus	1×10^5	0.009	0.004	2.133	0.368	0.784
South Africa	Chlorocebus sabaeus	1.8×10^5	0.009	0.011	0.874	0.368	0.322
Saint Kitts	Chlorocebus sabaeus	1.2×10^5	0.009	0.005	1.905	0.368	0.701
Zambia	Chlorocebus sabaeus	1.7×10^5	0.009	0.014	0.698	0.368	0.257
African	Homo sapiens	5.6×10^4	0.010	0.013	0.726	0.437	0.317
Admixed American	Homo sapiens	4.5×10^4	0.010	0.011	0.886	0.429	0.380
East Asian	Homo sapiens	4×10^4	0.010	0.008	1.213	0.426	0.517
European	Homo sapiens	4.1×10^4	0.010	0.009	1.104	0.422	0.466
South Asian	Homo sapiens	4.4×10^4	0.010	0.009	1.016	0.426	0.433

Table E: $\mathbb{P}[\mathcal{B}_0 \mid \mathcal{B}]$ **when including divergence to estimate** $\boldsymbol{S}$**.** N_e is the estimated effective population size. $\mathbb{P}[\mathcal{B}_0]$ (eq. 5) is the probability for a new mutation to be a non-adaptive mutation. These mutations have a selection coefficient predicted at the phylogenetic-scale larger than 1, thus toward a more fit amino-acid. $\mathbb{P}[\mathcal{B}]$ (eq. 15) is the probability for a mutation to be beneficial. These mutations have a selection coefficient at the population-scale larger than 1. $\mathbb{P}[\mathcal{B} \mid \mathcal{B}_0]$ (eq. 12) is the probability for a mutation to be beneficial at the population scale, given it is predicted to be a non-adaptive mutation at the phylogenetic scale (the *precision*). $\mathbb{P}[\mathcal{B}_0 \mid \mathcal{B}]$ (eq. 14) is the probability for a mutation to be non-adaptive given it is beneficial at the population scale (the *recall*). This probability is obtained using Bayes' formula.

4.3 Precision and recall

The estimation of *precision* and *recall* across populations when including substitutions in the terminal lineage for the estimation of S is shown in Table F. Comparing these results with tables 1, we acknowledge that the exact quantification of *precision* and *recall* is dependent on whether we included or not substitutions in the terminal lineage for the estimation of S. However, we can still see that non-adaptive beneficial mutations are positively selected compared to neutral and deleterious ones, and this result is valid whether we included or not substitutions in the terminal lineage for the estimation of S.

			**Deleterious mutations** $\mathcal{D} := S < -1$ $\mathcal{D}_0 := S_0 < -1$		**Nearly-neutral mutations** $\mathcal{N} := -1 < S < 1$ $\mathcal{N}_0 := -1 < S_0 < 1$		**Beneficial mutations** $\mathcal{B} := S > 1$ $\mathcal{B}_0 := S_0 > 1$	
**Population**	**Species**	N_e	**Precision** $\mathbb{P}[\mathcal{D} \mid \mathcal{D}_0]$	**Recall** $\mathbb{P}[\mathcal{D}_0 \mid \mathcal{D}]$	**Precision** $\mathbb{P}[\mathcal{N} \mid \mathcal{N}_0]$	**Recall** $\mathbb{P}[\mathcal{N}_0 \mid \mathcal{N}]$	**Precision** $\mathbb{P}[\mathcal{B} \mid \mathcal{B}_0]$	**Recall** $\mathbb{P}[\mathcal{B}_0 \mid \mathcal{B}]$
Equus c.	Equus caballus	7.5×10^4	0.937	0.955	0.111	0.192	0.498	0.106
Iran	Bos taurus	5.6×10^4	0.935	0.970	0.565	0.356	0.368	0.684
Uganda	Bos taurus	1.3×10^5	0.951	0.967	0.516	0.422	0.307	0.276
Australia	Capra hircus	1.7×10^5	0.952	0.970	0.598	0.452	0.178	0.294
France	Capra hircus	1.9×10^5	0.951	0.969	0.574	0.433	0.183	0.295
Iran (C. aegagrus)	Capra hircus	1.9×10^5	0.957	0.966	0.515	0.460	0.177	0.163
Iran	Capra hircus	2.3×10^5	0.951	0.967	0.542	0.422	0.184	0.273
Italy	Capra hircus	1.9×10^5	0.951	0.969	0.582	0.436	0.178	0.299
Morocco	Capra hircus	2.2×10^5	0.949	0.968	0.556	0.412	0.184	0.349
Iran	Ovis aries	3.8×10^5	0.963	0.962	0.421	0.424	0.189	0.214
Iran (O. orientalis)	Ovis aries	4.5×10^5	0.968	0.961	0.413	0.460	0.213	0.216
Iran (O. vignei)	Ovis aries	3.7×10^5	0.972	0.959	0.360	0.528	0.186	0.105
Various	Ovis aries	4.1×10^5	0.966	0.961	0.430	0.454	0.215	0.229
Morocco	Ovis aries	4×10^5	0.966	0.958	0.372	0.416	0.217	0.211
Barbados	Chlorocebus sabaeus	1.1×10^5	0.940	0.976	0.654	0.409	0.341	0.723
Central Afr. Rep.	Chlorocebus sabaeus	1.7×10^5	0.950	0.971	0.526	0.421	0.368	0.265
Ethiopia	Chlorocebus sabaeus	1.4×10^5	0.942	0.974	0.609	0.402	0.368	0.498
Gambia	Chlorocebus sabaeus	1.4×10^5	0.950	0.974	0.611	0.452	0.368	0.352
Kenya	Chlorocebus sabaeus	1.5×10^5	0.950	0.972	0.545	0.434	0.368	0.268
Nevis	Chlorocebus sabaeus	1×10^5	0.940	0.977	0.668	0.412	0.368	0.784
South Africa	Chlorocebus sabaeus	1.8×10^5	0.947	0.972	0.550	0.411	0.368	0.322
Saint Kitts	Chlorocebus sabaeus	1.2×10^5	0.940	0.976	0.646	0.407	0.368	0.701
Zambia	Chlorocebus sabaeus	1.7×10^5	0.950	0.971	0.527	0.426	0.368	0.257
African	Homo sapiens	5.6×10^4	0.911	0.980	0.666	0.341	0.437	0.317
Admixed American	Homo sapiens	4.5×10^4	0.902	0.976	0.580	0.284	0.429	0.380
East Asian	Homo sapiens	4×10^4	0.904	0.984	0.744	0.341	0.426	0.517
European	Homo sapiens	4.1×10^4	0.906	0.984	0.737	0.344	0.422	0.466
South Asian	Homo sapiens	4.4×10^4	0.907	0.984	0.737	0.349	0.426	0.433

Table F: **Precision and recall when including divergence to estimate S.** N_e is the estimated effective population size. *Precision* is the estimation of the selection coefficient at population scale (S) given that S_0 is known. *Recall* is the estimation of S_0 given selection coefficient at the population scale (S) is known. *Recall* for beneficial mutations ($\mathbb{P}[\mathcal{B}_0 \mid \mathcal{B}]$) is the probability for a mutation to be non-adaptive given it is beneficial at the population scale.

4.4 Misannotations of ancestral alleles

If misannotation is influencing the recall for $\mathcal{B}_0$ ($\mathbb{P}[\mathcal{B}_0 \mid \mathcal{B}]$), then the recall should increase as a function of divergence to the sister species (d_S: number of synonymous substitutions per sites) since divergence would increase the rate of misannotations. Shown in Figure F is recall $\mathbb{P}[\mathcal{B}_0 \mid \mathcal{B}]$ as a function of divergence (d_S) in which the latter is not an explanatory variable.

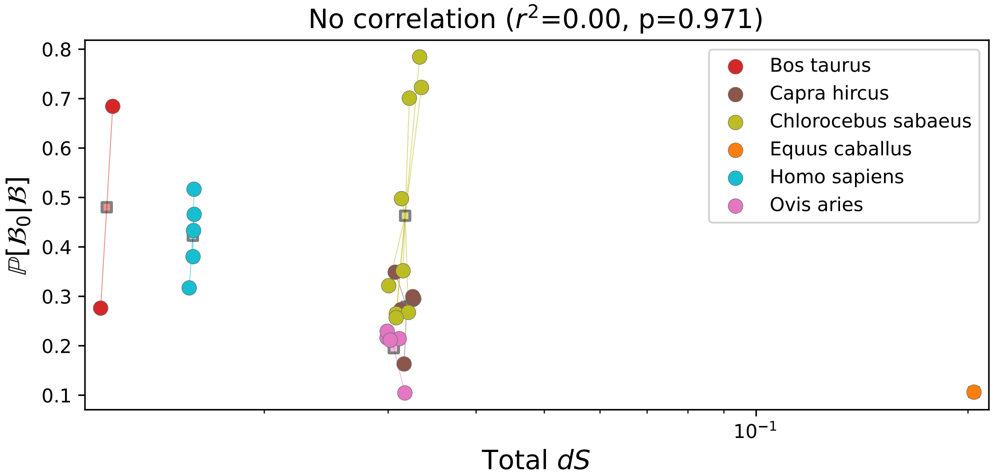

Figure F: **$\mathbb{P}[\mathcal{B}_0 \mid \mathcal{B}]$ when including divergence to estimate S, as a function of distance to the sister species**. $\mathbb{P}[\mathcal{B}_0 \mid \mathcal{B}]$ (eq. 14) is the probability for a beneficial mutation to be non-adaptive. d_S is number of synonymous substitutions per site that occurred since divergence with the sister species (closest species in the mammalian tree). Same as Figure A but including divergence to estimate S. Populations are represented by circles, and squares are the mean for the species. Correlations account for phylogenetic relationship and non-independence of samples, through the fit of a Phylogenetic Generalized Linear Model (see Materials & Methods).

4.5 Excluding genes under adaptation

The proportion of beneficial non-adaptive mutations ($\mathbb{P}[\mathcal{B}_0 \mid \mathcal{B}]$) is higher when excluding genes under adaptation, as shown in Table G, consistent with our expectation that genes with uniformly conserved functions should fit better the nearly-neutral equilibrium model.

Population	Species	Control	Case
Equus c.	Equus caballus	0.106	0.106
Iran	Bos taurus	0.684	0.755
Uganda	Bos taurus	0.276	0.310
Australia	Capra hircus	0.294	0.319
France	Capra hircus	0.295	0.325
Iran (C. aegagrus)	Capra hircus	0.163	0.174
Iran	Capra hircus	0.273	0.299
Italy	Capra hircus	0.299	0.321
Morocco	Capra hircus	0.349	0.419
Iran	Ovis aries	0.214	0.241
Iran (O. orientalis)	Ovis aries	0.216	0.213
Iran (O. vignei)	Ovis aries	0.105	0.089
Various	Ovis aries	0.229	0.196
Morocco	Ovis aries	0.211	0.206
Barbados	Chlorocebus sabaeus	0.723	0.833
Central Afr. Rep.	Chlorocebus sabaeus	0.265	0.285
Ethiopia	Chlorocebus sabaeus	0.498	0.547
Gambia	Chlorocebus sabaeus	0.352	0.571
Kenya	Chlorocebus sabaeus	0.268	0.265
Nevis	Chlorocebus sabaeus	0.784	0.892
South Africa	Chlorocebus sabaeus	0.322	0.307
Saint Kitts	Chlorocebus sabaeus	0.701	0.825
Zambia	Chlorocebus sabaeus	0.257	0.271
African	Homo sapiens	0.317	0.278
Admixed American	Homo sapiens	0.380	0.363
East Asian	Homo sapiens	0.517	0.499
European	Homo sapiens	0.466	0.456
South Asian	Homo sapiens	0.433	0.304

Table G: **$\mathbb{P}[\boldsymbol{\mathcal{B}_0} \mid \boldsymbol{\mathcal{B}}]$ excluding or not genes under adaptation, when including divergence to estimate $\boldsymbol{S}$.** Genes under adaptation retrieved from [3]. Comparison of $\mathbb{P}[\mathcal{B}_0 \mid \mathcal{B}]$ when including divergence for the whole genome (control) and when excluding genes under adaptation (case). The non-parametric Wilcoxon signed-rank test tests the null hypothesis that the distribution of the differences (case versus control) is symmetric about zero. Applied to the paired samples (case and control), Wilcoxon signed-rank test results in $s = 120$ with p-value $= 0.027$ for one-sided test (case higher than control).

4.6 Fitting one or three distributions of fitness effects

We also performed another test to assess that fitting the same functional form of DFE to three categories of selection (i.e., $\mathcal{D}_0, \mathcal{N}_0, \mathcal{B}_0$) is a sound methodology. Typically, $\mathbb{P}[\mathcal{B}]$ can be computed from two different estimates, by either splitting data (polymorphism within populations and substitutions in the terminal lineages) in three category (i.e., $\mathcal{D}_0, \mathcal{N}_0, \mathcal{B}_0$) or not at all. In the case of splitting data in three categories and fitting three DFEs, $\mathbb{P}[\mathcal{B}]$ is obtained by summing over the three categories using the law of total probability (eq. 15):

$$\mathbb{P}[\mathcal{B}] \text{ (three DFEs) } = \mathbb{P}[\mathcal{B} \mid \mathcal{D}_0] \times \mathbb{P}[\mathcal{D}_0] + \mathbb{P}[\mathcal{B} \mid \mathcal{N}_0] \times \mathbb{P}[\mathcal{N}_0] + \mathbb{P}[\mathcal{B} \mid \mathcal{B}_0] \times \mathbb{P}[\mathcal{B}_0]. \tag{S.1}$$

But it is also possible to compute $\mathbb{P}[\mathcal{B}]$ directly from fitting one DFE to the full data (polymorphism within populations and substitutions in the terminal lineages), not splitting at all, and then $\mathbb{P}[\mathcal{B}]$ is obtained from the parameters of the DFE (eq. 11):

$$\mathbb{P}[\mathcal{B}] \text{ (one DFE) } = \mathbb{P}[S > 1] = p_b \int_1^{+\infty} f_e(S; \beta_b) \mathrm{d}S. \tag{S.2}$$

We reproduced this experiment for $\mathbb{P}[\mathcal{D}]$ (Figure G), $\mathbb{P}[\mathcal{N}]$ (Figure H) and $\mathbb{P}[\mathcal{B}]$ (Figure I).

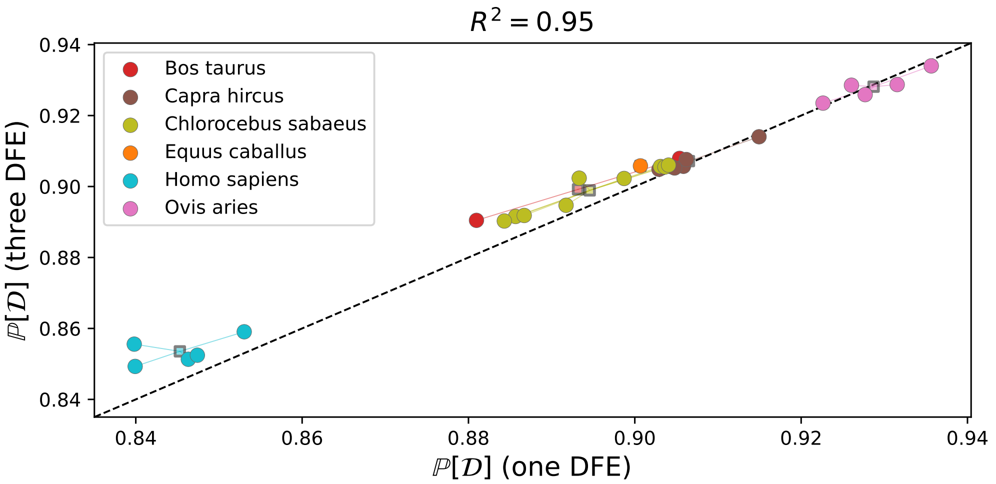

Figure G: $\mathbb{P}[\mathcal{D}]$ **when fitting a single or three DFEs.** Populations are represented by circles, and squares are the mean for the species. Goodness of fit is calculated as the coefficient of determination (R^2) for the prediction $\mathbb{P}[\mathcal{D}]$ (three DFEs) $= \mathbb{P}[\mathcal{D}]$ (one DFE) , meaning calculating the residuals of the fit to the 1:1 line.

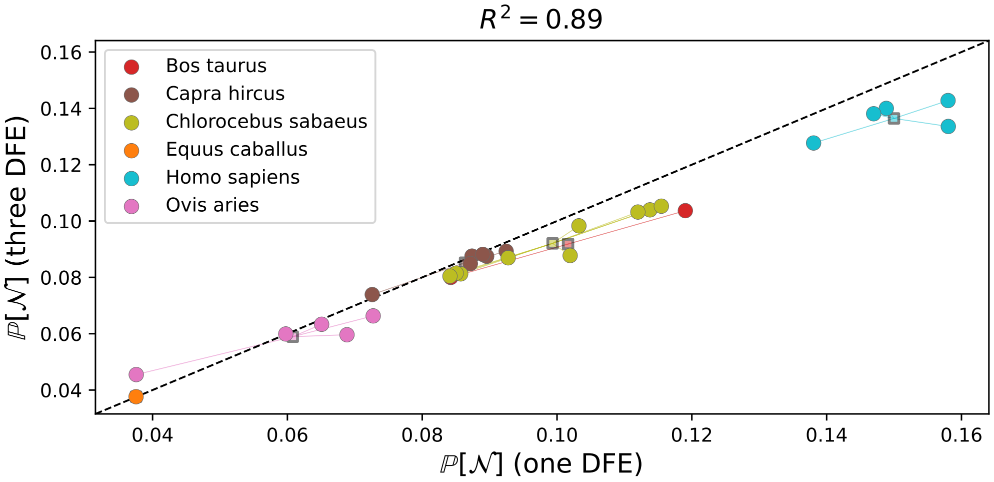

Figure H: $\mathbb{P}[\mathcal{N}]$ **when fitting a single or three DFEs.** Populations are represented by circles, and squares are the mean for the species. Goodness of fit is calculated as the coefficient of determination (R^2) for the prediction $\mathbb{P}[\mathcal{N}]$ (three DFEs) $= \mathbb{P}[\mathcal{N}]$ (one DFE) , meaning calculating the residuals of the fit to the 1:1 line.

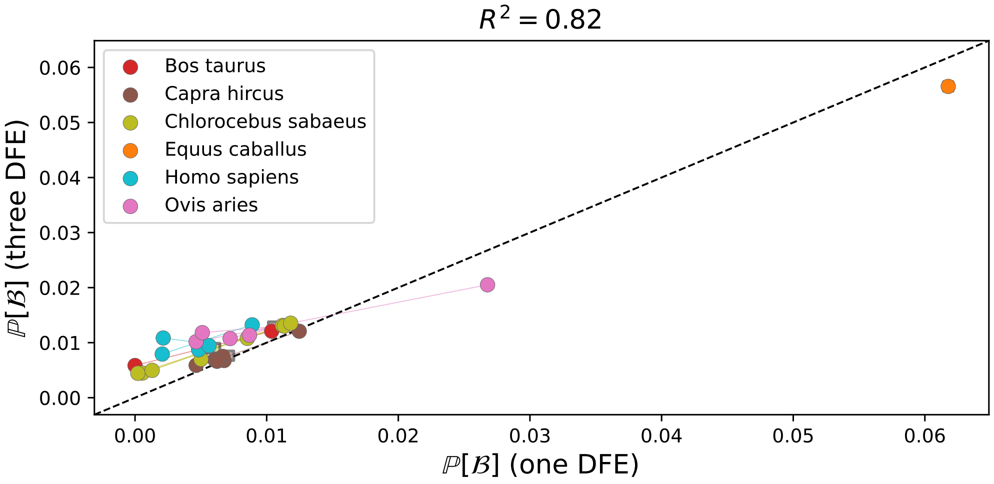

Figure I: $\mathbb{P}[\mathcal{B}]$ **when fitting a single or three DFEs.** Populations are represented by circles, and squares are the mean for the species. Goodness of fit is calculated as the coefficient of determination (R^2) for the prediction $\mathbb{P}[\mathcal{B}]$ (three DFEs) $= \mathbb{P}[\mathcal{B}]$ (one DFE) , meaning calculating the residuals of the fit to the 1:1 line.

5 Non-parametric distribution of fitness effects

5.1 polyDFE model D (non-parametric)

Additionally to including divergence, we also tested our prediction with polyDFE model D instead of model C. In polyDFE model D, the DFE of non-synonymous mutations is given as a discrete DFE of K categories (instead of a continuous distribution in model C), where the selection coefficients of each category i $(1 \leq i \leq K)$ are fixed parameters S_i, and each value S_i has a probability p_i (estimated), with $\sum_{i=1}^{K} p_i = 1$. We used $K = 6$ with $S_1 = -500$, $S_2 = -4$, $S_3 = -1$, $S_4 = 0$, $S_5 = 1$, $S_6 = 4$.

For each class of selection x, the parameters p_i $(i \in \{1 \leq i \leq 6\})$ were used to compute $\mathbb{P}[\mathcal{D} \mid x]$, $\mathbb{P}[\mathcal{N} \mid x]$, and $\mathbb{P}[\mathcal{B} \mid x]$ as:

$$\mathbb{P}[\mathcal{D} \mid x] = \mathbb{P}[S < -1 \mid x] = p_1 + p_2 \tag{S.3}$$

$$\mathbb{P}[\mathcal{N} \mid x] = \mathbb{P}[-1 < S < 1 \mid x] = p_3 + p_4 + p_5, \tag{S.4}$$

$$\mathbb{P}[\mathcal{B} \mid x] = \mathbb{P}[S > 1 \mid x] = p_6. \tag{S.5}$$

5.2 Probability for beneficial mutations to be non-adaptive

The estimation of $\mathbb{P}[\mathcal{B}]$ and $\mathbb{P}[\mathcal{B}_0 \mid \mathcal{B}]$ across populations when including substitutions in the terminal lineage and fitting a discrete DFE to S is shown in Table H. Altogether, comparing these results with table E, we acknowledge that the exact proportion of non-adaptive among all beneficial mutations is dependent on the model used to estimate the DFE. However, we can still see that non-adaptive beneficial mutations are positively selected compared to neutral and deleterious ones, and this result is valid for different underlying DFE.

Population	Species	N_e	$\mathbb{P}[\mathcal{B}_0]$	$\mathbb{P}[\mathcal{B}]$	$\frac{\mathbb{P}[\mathcal{B}_0]}{\mathbb{P}[\mathcal{B}]}$	$\mathbb{P}[\mathcal{B} \mid \mathcal{B}_0]$	$\mathbb{P}[\mathcal{B}_0 \mid \mathcal{B}]$
Equus c.	Equus caballus	7.5×10^4	0.012	0.006	2.050	0.163	0.334
Iran	Bos taurus	5.6×10^4	0.011	0.005	2.125	0.133	0.283
Uganda	Bos taurus	1.3×10^5	0.011	0.010	1.139	0.140	0.159
Australia	Capra hircus	1.7×10^5	0.011	0.002	5.637	0.151	0.850
France	Capra hircus	1.9×10^5	0.011	0.002	6.117	0.154	0.940
Iran (C. aegagrus)	Capra hircus	1.9×10^5	0.011	0.002	5.842	0.156	0.911
Iran	Capra hircus	2.3×10^5	0.011	0.002	6.400	0.147	0.943
Italy	Capra hircus	1.9×10^5	0.011	0.002	5.793	0.155	0.895
Morocco	Capra hircus	2.2×10^5	0.011	0.003	4.192	0.150	0.627
Iran	Ovis aries	3.8×10^5	0.012	0.020	0.584	0.154	0.090
Iran (O. orientalis)	Ovis aries	4.5×10^5	0.011	0.021	0.535	0.148	0.079
Iran (O. vignei)	Ovis aries	3.7×10^5	0.012	0.020	0.568	0.151	0.086
Various	Ovis aries	4.1×10^5	0.011	0.021	0.553	0.152	0.084
Morocco	Ovis aries	4×10^5	0.012	0.022	0.529	0.153	0.081
Barbados	Chlorocebus sabaeus	1.1×10^5	0.009	0.002	5.152	0.170	0.877
Central Afr. Rep.	Chlorocebus sabaeus	1.7×10^5	0.009	0.007	1.266	0.157	0.199
Ethiopia	Chlorocebus sabaeus	1.4×10^5	0.009	0.002	3.979	0.161	0.640
Gambia	Chlorocebus sabaeus	1.4×10^5	0.009	0.008	1.201	0.159	0.191
Kenya	Chlorocebus sabaeus	1.5×10^5	0.009	0.003	2.801	0.165	0.461
Nevis	Chlorocebus sabaeus	1×10^5	0.009	0.002	5.361	0.169	0.908
South Africa	Chlorocebus sabaeus	1.8×10^5	0.009	0.005	1.912	0.154	0.294
Saint Kitts	Chlorocebus sabaeus	1.2×10^5	0.009	0.002	4.561	0.165	0.753
Zambia	Chlorocebus sabaeus	1.7×10^5	0.009	0.004	2.661	0.153	0.406
African	Homo sapiens	5.6×10^4	0.010	0.010	0.997	0.154	0.154
Admixed American	Homo sapiens	4.5×10^4	0.010	0.008	1.206	0.158	0.190
East Asian	Homo sapiens	4×10^4	0.010	0.007	1.455	0.163	0.237
European	Homo sapiens	4.2×10^4	0.010	0.007	1.316	0.167	0.219
South Asian	Homo sapiens	4.4×10^4	0.010	0.007	1.340	0.164	0.219

Table H: $\mathbb{P}[\mathcal{B}_0 \mid \mathcal{B}]$ **when using a discrete DFE to estimate** S**.** N_e is the estimated effective population size. $\mathbb{P}[\mathcal{B}_0]$ (eq. 5) is the probability for a new mutation to be a non-adaptive beneficial mutation. These mutations have a selection coefficient predicted at the phylogenetic-scale larger than 1, thus toward a more fit amino-acid. $\mathbb{P}[\mathcal{B}]$ (eq. 15) is the probability for a mutation to be beneficial. These mutations have a selection coefficient at the population-scale larger than 1. $\mathbb{P}[\mathcal{B} \mid \mathcal{B}_0]$ (eq. 12) is the probability for a mutation to be beneficial at the population scale, given it is predicted to be a beneficial non-adaptive mutation at the phylogenetic scale (the *precision*). $\mathbb{P}[\mathcal{B}_0 \mid \mathcal{B}]$ (eq. 14) is the probability for a mutation to be non-adaptive given it is beneficial at the population scale (the *recall*). This probability is obtained using Bayes' formula.

5.3 Precision and recall

The estimation of *precision* and *recall* across populations when including substitutions in the terminal lineage and fitting a discrete DFE to S is shown in Table I. Altogether, comparing these results with table F, we acknowledge that the exact quantification of *precision* and *recall* is dependent on the model used to estimate the DFE. However, we can still see that non-adaptive beneficial mutations are positively selected compared to neutral and deleterious ones, and this result is valid for different underlying DFE.

			**Deleterious mutations** $\mathcal{D} := S < -1$ $\mathcal{D}_0 := S_0 < -1$		**Nearly-neutral mutations** $\mathcal{N} := -1 < S < 1$ $\mathcal{N}_0 := -1 < S_0 < 1$		**Beneficial mutations** $\mathcal{B} := S > 1$ $\mathcal{B}_0 := S_0 > 1$	
**Population**	**Species**	N_e	**Precision** $\mathbb{P}[\mathcal{D} \mid \mathcal{D}_0]$	**Recall** $\mathbb{P}[\mathcal{D}_0 \mid \mathcal{D}]$	**Precision** $\mathbb{P}[\mathcal{N} \mid \mathcal{N}_0]$	**Recall** $\mathbb{P}[\mathcal{N}_0 \mid \mathcal{N}]$	**Precision** $\mathbb{P}[\mathcal{B} \mid \mathcal{B}_0]$	**Recall** $\mathbb{P}[\mathcal{B}_0 \mid \mathcal{B}]$
Equus c.	Equus caballus	7.5×10^4	0.911	0.974	0.645	0.319	0.163	0.334
Iran	Bos taurus	5.6×10^4	0.895	0.973	0.639	0.288	0.133	0.283
Uganda	Bos taurus	1.3×10^5	0.951	0.974	0.664	0.492	0.140	0.159
Australia	Capra hircus	1.7×10^5	0.912	0.984	0.834	0.386	0.151	0.850
France	Capra hircus	1.9×10^5	0.912	0.983	0.819	0.381	0.154	0.940
Iran (C. aegagrus)	Capra hircus	1.9×10^5	0.928	0.980	0.767	0.408	0.156	0.911
Iran	Capra hircus	2.3×10^5	0.955	0.969	0.616	0.459	0.147	0.943
Italy	Capra hircus	1.9×10^5	0.911	0.983	0.822	0.379	0.155	0.895
Morocco	Capra hircus	2.2×10^5	0.950	0.976	0.706	0.468	0.150	0.627
Iran	Ovis aries	3.8×10^5	0.983	0.958	0.421	0.796	0.154	0.090
Iran (O. orientalis)	Ovis aries	4.5×10^5	0.982	0.961	0.448	0.806	0.148	0.079
Iran (O. vignei)	Ovis aries	3.7×10^5	0.974	0.963	0.480	0.685	0.151	0.086
Various	Ovis aries	4.1×10^5	0.982	0.964	0.500	0.822	0.152	0.084
Morocco	Ovis aries	4×10^5	0.983	0.958	0.387	0.782	0.153	0.081
Barbados	Chlorocebus sabaeus	1.1×10^5	0.895	0.987	0.860	0.351	0.170	0.877
Central Afr. Rep.	Chlorocebus sabaeus	1.7×10^5	0.925	0.974	0.653	0.373	0.157	0.199
Ethiopia	Chlorocebus sabaeus	1.4×10^5	0.892	0.980	0.763	0.320	0.161	0.640
Gambia	Chlorocebus sabaeus	1.4×10^5	0.939	0.982	0.770	0.469	0.159	0.191
Kenya	Chlorocebus sabaeus	1.5×10^5	0.916	0.976	0.680	0.345	0.165	0.461
Nevis	Chlorocebus sabaeus	1×10^5	0.895	0.989	0.884	0.358	0.169	0.908
South Africa	Chlorocebus sabaeus	1.8×10^5	0.930	0.971	0.604	0.361	0.154	0.294
Saint Kitts	Chlorocebus sabaeus	1.2×10^5	0.898	0.986	0.839	0.353	0.165	0.753
Zambia	Chlorocebus sabaeus	1.7×10^5	0.912	0.975	0.676	0.335	0.153	0.406
African	Homo sapiens	5.6×10^4	0.945	0.976	0.627	0.431	0.154	0.154
Admixed American	Homo sapiens	4.5×10^4	0.936	0.977	0.641	0.397	0.158	0.190
East Asian	Homo sapiens	4×10^4	0.943	0.977	0.648	0.422	0.163	0.237
European	Homo sapiens	4.2×10^4	0.945	0.977	0.644	0.429	0.167	0.219
South Asian	Homo sapiens	4.4×10^4	0.945	0.977	0.644	0.430	0.164	0.219

Table I: **Precision and recall when using a discrete DFE to estimate S.** N_e is the estimated effective population size. *Precision* is the estimation of the selection coefficient at population scale (S) given that S_0 is known. *Recall* is the estimation of S_0 given selection coefficient at the population scale (S) is known. *Recall* for beneficial mutations ($\mathbb{P}[\mathcal{B}_0 \mid \mathcal{B}]$) is the probability for a mutation to be non-adaptive given it is beneficial at the population scale.

References

1. Sawyer, S. A. & Hartl, D. L. Population Genetics of Polymorphism and Divergence. *Genetics* **132** (1992).
2. Tataru, P., Mollion, M., Glémin, S. & Bataillon, T. Inference of Distribution of Fitness Effects and Proportion of Adaptive Substitutions from Polymorphism Data. *Genetics* **207,** 1103–1119 (2017).
3. Latrille, T., Rodrigue, N. & Lartillot, N. Genes and Sites under Adaptation at the Phylogenetic Scale Also Exhibit Adaptation at the Population-Genetic Scale. *Proceedings of the National Academy of Sciences of the United States of America* **120,** e2214977120 (2023).
