# Supplementary material for: Estimating the proportion of beneficial mutations that are not adaptive in mammals
Source: PLoS Genet. 2024 Dec 26;20(12):e1011536. doi: 10.1371/journal.pgen.1011536 (PMC11709321; doi:10.1371/journal.pgen.1011536)
Supplement: Attachment — Submitted filename: PGENETICS-D-24-00471R-Response-to-reviewers.pdf [file pgen.1011536.s005.pdf]

Response to editor and point-by-point response to reviewers' comments on Latrille *et al.*

Editor:

If you choose to submit a revised manuscript, please address all of the comments from the reviewers. When revising your manuscript, please pay special attention to the following points:

We are grateful for the comments raised by the three reviewers and the editor, all of which greatly enriched our perspective on the questions addressed in our manuscript. In the revised manuscript, we have tested the performance of our approach more thoroughly and have acknowledged and discussed the limitations of our approach and the consequences of our results more extensively. We have restructured the manuscript for better readability, as suggested by reviewer II. Also, following a recurrently raised comment regarding the inclusion of substitutions in the terminal lineages rather than relying solely on currently segregating mutations to quantify selection, we have also leveraged both polymorphism and divergence to estimate the distribution of fitness effects (DFE). We have added a new section, *Selection in the terminal lineage and in populations*, that describes these results.

1) Please add some simulations to address the performance of your approach under the assumptions of the model.

Although the core of the study is the test of our approach on empirical data (mammalian population-genetics and phylogenetic datasets), we completely agree that simulations are also essential to test the performance of our approach. As such, we performed simulations at the population-genetic level to test whether our method is able to recover the proportion of beneficial mutations that are non-adaptive in synthetic polymorphism datasets. We now show that our method is able to retrieve such proportions in a simulated setting, thus making the empirical results more robust. This argument is more detailed in the response to reviewer III.

2) Reviewer 3 raises concerns about the functional form for the DFE that you're fitting. Please carefully address these points.

We agree that the functional form of the fitted DFE can be misspecified. However, from the simulations that we have now performed, and with two other controls that we also have performed, we argue that we now assess the impact of the functional form of the DFE on our method. This rationale is explained in more detail in the response to reviewer III.

3) Reviewer 2 asks questions pertaining to the role that changing fitness landscapes could play in the inference. Reviewer 2 also raises concerns about the correlation of the patterns with effective population size. Please resolve this point.

We now more thoroughly describe the role that changing fitness landscapes could play in the inference (either through changes in N_e or changing amino-acid preferences *sensu stricto*). We describe this argument in more detail in the response to reviewer II.

Reviewer I:

The authors provide a very careful evaluation of the fitness effect of non-synonymous mutations using both population genetic and phylogenetic (multi-species alignment) data and methods. They suggest that a substantial proportion (between 15-45%) of all new beneficial mutations are “non-adaptive”, i.e. they restore fitness that was lost due to a previous fixation of a slightly deleterious mutation and thus are not responding to a changed environment. This is as far as I know the first quantitative assessment of this idea. A central assumption is that the fitness landscape of amino acid replacements can be estimated from phylogenetic data and that this fitness landscape stays constant over time. There are some potential model violations (changing population size, epistasis, CpG mutation bias) and the authors provide a fair assessment of each of these potential issues and how these could affect the precision of the estimated effect.

I don’t have any major concerns. This is a careful and insightful study and I fully endorse publication in PLOS Genetics. Below, I’ve listed a few suggestions for the discussion.

Thank you very much for these comments.

I think it would be useful to have a more explicit discussion of strongly beneficial mutations. Rare but strongly beneficial mutations would not be inferred by either of the methods, but can contribute substantially to divergence. I think it would help to mention that the inferred beneficial (non-adaptive) mutations are relatively weak and fall within a narrow range of selection strength ($1<S<10$). I also wonder if it is possible to calculate how much non-adaptive beneficial mutations contribute to nonsynonymous divergence, relative to all beneficial mutations. E.g., how much would they inflate estimates of the rate of adaptive evolution (alpha)?

This is a very interesting point and we completely agree that we did not discuss how non-adaptive beneficial mutations contribute to non-synonymous divergence enough.

First, we updated the text in several places to mention that inferred non-adaptive beneficial mutations are indeed relatively weak. We also took this opportunity to clarify the relationship between selection coefficient and d_N/d_S. Indeed, $\omega=d_N/d_S$ can be related to the underlying scaled selection coefficient (S) with the relation $\omega=S/(1-\exp(-S))$ as in *Nielsen & Yang* (2003, eq. 3). In our experiments, our observed $d_N(\mathcal{B}_0)/d_S$ thus translates into S values between 0.32 and 1.24, so indeed only slightly advantageous. We argue that the reason we can observe such a slight effect is because of our exome-wide study, aggregating information across many sites. Similar to GWAS that have limited power for a single locus but still are able to uncover large parts of heritable phenotypic variations, we argue that even if single sites are almost non-informative, we can detect the aggregate effect.

Accordingly, we updated the section *Selection along the terminal branches* in the *Results* from:

"We obtained values between 1.17 and 1.75 in the different lineages (table S1), meaning that $\mathcal{B}_0$ mutations reach fixation slightly more frequently than synonymous mutations that are supposed to be neutral, consistent with these $\mathcal{B}_0$ mutations being weakly beneficial."

to:

"We obtained values between 1.17 and 1.75 in the different lineages (table S2), meaning that $\mathcal{B}_0$ mutations reach fixation slightly more frequently than synonymous mutations that are supposed to be neutral. Such observation is consistent with the premise that $\mathcal{B}_0$ mutations are weakly beneficial, translating to a scaled selection coefficient between 0.32 and 1.24 (table S2). Finding $d_N(\mathcal{B}_0)/d_S>1$ for these sites confirms that these sites are closer to optimality at the end of the branch than at the beginning. The fact that the cause of the maladaptation is the previous fixation of deleterious mutations rather than environmental change does not change the fact that this subset of sites has contributed positively to the population. It is just that under the stationary process the increase in fitness at these sites is offset by deleterious substitutions elsewhere in the genome so that there is no net adaptation."

- Rasmus Nielsen, Ziheng Yang, *Estimating the Distribution of Selection Coefficients from Phylogenetic Data with Applications to Mitochondrial and Viral DNA*, Molecular Biology and Evolution, Volume 20, Issue 8, August 2003, Pages 1231–1239, https://doi.org/10.1093/molbev/msg147

Unfortunately, with our current approach, we do not have an estimate of the selection coefficient of adaptive mutations. Therefore, while we can estimate the proportion of beneficial mutations that are non-adaptive, we cannot yet extend this to substitutions in a general framework. We also agree that strongly beneficial mutations can contribute substantially to divergence, thus rather than relying solely on currently segregating mutations to quantify selection, we also leveraged both polymorphism within a population together with substitutions in the terminal lineage to estimate the distribution of fitness effects (DFE), and we have added in the *Results* a new section, *Selection in the terminal lineage and in populations,* that describes these results. Furthermore, estimating how much non-adaptive beneficial mutations contribute to nonsynonymous divergence is also discussed in comment #5 by reviewer III.

Related to this, could the authors comment on the study by Enard et al. (eLife, 2016, https://doi.org/10.7554/eLife.12469), which states that viruses are dominantly driving protein adaptation in mammals? They estimate that close to 30% of adaptive amino acid changes are driven by adaptation to viruses. Is this consistent with or contradicting the results of the present study?

This is a very interesting view. In our study, we described adaptation as a change in the environment. We need to emphasize that these changes are either abiotic (temperature, humidity, ...) or biotic (prey, predators, viruses, ...). Thus we believe that while it is orthogonal to our finding (nor consistent not contradicting), we agree that it should be mentioned to give more context into how to split reasons behind a mutation being advantageous and how we define a change in the environment.

We included the study by Enard *et al.* (2016) in the *Introduction*:

"Evolutionary adaptation is tightly linked to environmental change and species responding to this change [2-3]."

to:

"Evolutionary adaptation is tightly linked to environmental change and species responding to this change [2-3]. Such environmental changes are either abiotic (e.g. temperature, humidity) or biotic such as pressure from predators or viruses (Enard *et al.*, 2016)"

We also included the study by Enard *et al.* (2016) in the Discussion to illustrate changes in amino-acid fitnesses (see comment #1 by reviewer II):

"To visualize an example of a wrongly predicted nearly-neutral mutation, we can first imagine a site where only hydrophilic amino acids are accepted because of the protein properties (e.g. a surface site of a globular protein). Let us then assume that such a site is also a target for viruses, hence promoting amino-acid changes which modify the site's viral affinity (Enard *et al.*, 2016). Given the selective pressure favoring amino-acid change, but restricting the possibilities to hydrophilic amino acids, most hydrophilic amino acids will likely be visited along the phylogenetic tree and the mutation-selection model will give high and similar fitnesses to all of them."

Reviewer II:

In this paper, Latrille, Joseph et al. combine phylogenetic and population genetic methods to infer the contribution of beneficial non-adaptive mutations to molecular evolution. They define beneficial non-adaptive mutations as mutations that revert deleterious amino acid changes that have been fixed due to drift to their previous, beneficial state. This is contrasted with adaptive mutations, which increase in frequency due to positive selection after a change in the fitness landscape, compensatory mutations, which restore ancestral fitness through a change at a different amino acid than the initial deleterious change, and nearly neutral mutations, which have little fitness effect. They present evidence that ongoing positive selection is driven by a combination of beneficial non-adaptive mutations as well as adaptive mutations, and the proportion of beneficial non-adaptive mutations may be substantial. In addition, they make the point that a model that includes positively-selected beneficial non-adaptive mutations is a more appropriate null model for the evolution of protein-coding genes than is a model of no positive selection. Overall, this work is an interesting and important contribution to our understanding of adaptation and positive selection.

Major comments

My major comments are centered on two main things: The assumption of a stable underlying fitness landscape, and the consequences of the dependence on effective population size.

Stable underlying fitness landscape:

Overall, I would appreciate more clarification and reasoning for why the assumption of a stable underlying fitness landscape is reasonable in this case. It is necessary for the model employed, which makes it extremely important to both justify its use and consider the consequences if the assumption is violated. In the scenario as formulated, the presence of adaptive mutations means the fitness landscape is not entirely stable (acknowledged by the authors eg. P4L113-114 “we could tease apart beneficial non-adaptive from adaptive mutations resulting from a change in the fitness landscape”). Is the implication then that the fitness landscape is stable for some proteins, but not for others? If so, this could have interesting implications for understanding the relative proportions of beneficial non-adaptive mutations vs. adaptive mutations.

We completely agree that theoretically the fitness landscape should be stable for some proteins, but not for others, and this could be tested empirically. Previous studies actually sought deviation from the mutation-selection equilibrium (under a stable underlying fitness landscape) to detect genes under adaptation (Rodrigue & Lartillot, 2017; Rodrigue *et al.,* 2021; Latrille *et al.,* 2023). Empirically, for roughly 5% of the mammalian orthologs, the assumption of a stable fitness landscape could not explain the observed rate of protein evolution across the phylogeny. However, and overall, across the exome the rate of evolution is as predicted under a stable fitness landscape (fig 1A in Latrille *et al.,* 2023). So, we agree that, empirically, the fitness landscape is stable for some proteins but not for others. We would be thrilled to be able to compute $P[\mathcal{B}_0|\mathcal{B}]$ on a per gene basis and study its relationship to other metrics of evolution, however and unfortunately, there is not enough polymorphism available per gene to compute $P[\mathcal{B}_0|\mathcal{B}]$, which requires pooling data across many genes.

Furthermore, what would be the theoretical consequences if this assumption were relaxed? The authors start to approach this in one way by re-conducting the analysis using highly conserved genes (P7L208-211), which might be expected to have a more stable fitness landscape than other genes that are likely to adapt to changing environmental conditions. I would appreciate more discussion of the implications of the findings associated with that version of the analysis in general, and specifically as they might relate to the assumption of a stable fitness landscape.

We have added more discussion on this point. In particular, we have changed the sentence from:

"Moreover, because the phylogenetic mutation-selection codon model should fit better for genes with uniformly conserved functions, as a control, we filtered out genes under pervasive adaptation [44], and indeed we found an increase in the proportion of beneficial mutations that are non-adaptive (P[$\mathcal{B}_0$|$\mathcal{B}$]), consistent with our expectation."

to:

"Finally, because the phylogenetic mutation-selection codon model should fit better for genes with uniformly conserved functions, we filtered out genes under pervasive adaptation [44] as a control. In this subset of the exome, containing genes with a more stable fitness landscape, we found an increase in the proportion of beneficial mutations that are non-adaptive (Wilcoxon signed-rank, s=80, p=0.002, table S10), consistent with our expectation that beneficial mutations occur more frequently in genes under changing fitness landscapes."

In addition, could the presence of (an unknown number of) adaptive mutations impact the fitting of the mutation-selection model to the multi-species alignments, and impact the estimation of the amino-acid fitness landscape itself?

This is a very interesting point which we have now addressed in line with the following idea. If the fitness landscape is changing with time, so called a fitness seascape (Mustonen & Lässig, 2008), what the phylogenetic mutation-selection model is estimating as a fitness landscape is an average over the phylogeny of the seascapes. In this case, mutations will be predicted as nearly-neutral ($\mathcal{N}_0$), which will ultimately inflate P[$\mathcal{B}$|$\mathcal{N}_0$], making our estimate conservative.

This was the idea behind our sentence *"Instead, in our analysis, to be conservative, we considered that any mutation under positive selection at the population scale ($\mathcal{B}$) but not predicted as such at the phylogenetic scale (not $\mathcal{B}_0$) is potentially an adaptation."* but we completely agree that there is a lot to unpack here and we shall be more descriptive.

We have modified the *Discussion* to convey this point in more detail, adding a specific example. Specifically, we have changed from:

"To be conservative, we considered that any mutation under positive selection at the population scale ($\mathcal{B}$) but not predicted as such at the phylogenetic scale (not $\mathcal{B}_0$) is potentially an adaptation."

to:

"To be conservative, we considered mutations as adaptive if they were detected as being under positive selection at the population scale despite them being either incorrectly predicted as deleterious ($\mathcal{D}_0$) or nearly-neutral ($\mathcal{N}_0$:=-1<S_0<1) from the amino-acid mammalian fitnesses. An example of an incorrectly

predicted deleterious mutation ($\mathcal{D}_0$) from its fitness landscape could be an amino acid having always been deleterious across mammals, but being advantageous ($\mathcal{B}$) in the current species due to environmental changes or a major shift in their fitness landscape (e.g. domestication). To visualize an example of a wrongly predicted nearly-neutral mutation, we can first imagine a site where only hydrophilic amino acids are accepted because of the protein properties (e.g. a surface site of a globular protein). Let us then assume that such a site is also a target for viruses, hence promoting amino-acid changes which modify the site's viral affinity (Enard *et al.*, 2016). Given the selective pressure favoring amino-acid change, but restricting the possibilities to hydrophilic amino acids, most hydrophilic amino acids will likely be visited along the phylogenetic tree and the mutation-selection model will give high and similar fitnesses to all of them. In such a case, any mutation between hydrophilic amino acids will be wrongly predicted as nearly-neutral ($\mathcal{N}_0$), while it is in fact adaptive. In summary, under a changing fitness landscapes (Mustonen & Lässig, 2008), our phylogenetic mutation-selection model takes an average over fitness changes observed along the phylogeny, causing beneficial mutations ($\mathcal{B}$) to be predicted as either deleterious ($\mathcal{D}_0$) or nearly-neutral ($\mathcal{N}_0$), therefore mechanically reducing $P[\mathcal{B}_0|\mathcal{B}]$ and making our estimate conservative."

This potential concern is especially pronounced for species that have been undergoing domestication, and as a result have had major changes in their fitness landscapes in the relatively recent past. Four of the six species tested (Ovis, Capra, Bos, Equus) are domestic species, and as a result are expected to have had strong adaptive responses to a major shift in their fitness landscape, potential changes in the DFE as a result (Rice et al. 2015 Genetics), and additionally recent large reductions in effective population size.

We have now described this specific case within the paragraph transcribed in the previous answer. The sentence reads: "An example of an incorrectly predicted deleterious mutation ($\mathcal{D}_0$) from its fitness landscape could be an amino acid having always been deleterious across mammals, but being advantageous ($\mathcal{B}$) in the current species due to environmental changes or a major shift in their fitness landscape (e.g. domestication)."

Effective population size:
Because the definition of mutation categories deleterious, nearly-neutral, and (non-adaptive) beneficial is relative to S_0, and $S_0 = 4N_e \times s$, the categorization of mutations necessarily depends on species/population N_e. This is appropriate, since efficiency of selection depends on N_e, but it means that the N_e estimates are very important to the results. I appreciate that the authors took a consistent approach based on θ, but their reported N_e values are 1-2 orders of magnitude higher for many of these species than often reported in the literature (for example, see Warren et al. 2015 for Chlorocebus sabaeus; Eyidivandi et al. 2020 Animal Genetics, Taheri et al. 2022 Small Ruminant Research for Ovis aries spp. Taheri et al. also used the NextGen data.). If the N_es reported here are biased upward, that will also bias the proportion of mutations determined to be in each category.

The effective population size estimated in Eyidivandi et al. (2020) and Taheri et al. (2022) are not based on θ, but on LD, which gives an estimate of population size which is much more recent (only a hundreds generations in Eyidivandi et al. (2020)), and impacted by domestication. The estimate of effective population size given in Warren (2015) uses an approach comparable to ours, but uses a human mutation rate of 1.5×10^{-8} which is twice as high as the one estimated on new world monkeys in Bergeron *et al.* (2023). When correcting for it, the effective population size reported by Eyidivandi et al. (2020) is quite coherent with the one in this study. Overall, the unbiased estimation of effective population size is quite difficult and is the object of a whole field of research. Here, instead we applied the same well established estimator (θ) to all populations, such as to be able to compare them.

In addition, phylogenetic regressions are conducted to understand the relationship between the proportion of mutations in each category at the population scale and at the population scale given its category at the phylogenetic scale. However the expectations of these regressions are not straightforward, since $P[\mathcal{B}]$, $P[\mathcal{B}_0|\mathcal{B}]$, and the proportions of the other categories are, by definition, dependent on N_e. As a result, the expected relationships are dependent on $N_e \times s$. That is, a relationship between the dependent variable and N_e is baked in.

Indeed, the impact of N_e on the proportion of beneficial mutations that are non-adaptive is not trivial and would require a formal theoretical investigation on its own. In the *Discussion* we sought to describe this relationship empirically with the few populations we have, because we have the opportunity to do so, and discuss why we do not observe any relationship.

The research on beneficial non-adaptive mutations is quite scarce, and we hope that these empirical observations, along with hypotheses given in the *Discussion*, will motivate further theoretical investigations.

The section *Assumptions and methodological limitations* has been largely updated (see previous comment) and moved earlier in the *Discussion* since it serves as foundation to discuss the influence of effective population size.

Minor comments

- The structure of the manuscript is at times hard to follow, with material in the introduction that seems to fit better in the methods, etc. I ask the authors to revisit the overall structure during their revisions and make sure that the organization is straightforward. Some examples:
- Some methodological descriptions in introduction rather than methods, these make it hard to follow/find these details (eg. P3L90-98)
- P8L250-251 should be in results
- P16L486-487 “we fitted a Phylogenetic Generalized Linear Model in R with the package caper” it is unclear without more context (found only in the results and discussion) what the nature of this model is.

We appreciate the comment and consider that the manuscript has benefitted from the restructuring proposed. We have also changed the following:

- Some description in the *Introduction* have been moved to *Materials & Methods*
- The *Introduction* does not use any mathematical notation anymore to be more straightforward.
- Any results that were in the *Discussion* were moved to the *Results* section.
- A new section, *Selection in the terminal lineage and in populations*, was added to the *Results*.
- The section *Assumptions and methodological limitations* has been moved earlier in the *Discussion* since it serves as foundation to discuss the influence of effective population size and the role of epistasis and compensatory mutations.

We hope the manuscript is easier to follow now and thank you for these suggestions.

- Eqs. 9 and 10 are inconsistent with eq. 8, showing $f_\Gamma(S; -\beta_d, b)$ vs. $f_\Gamma(-S; -\beta_d, b)$. Is this intentional?

This change of sign is indeed intentional in the integrand of eqs. 9-10. In eq. 8, since $S \leq 0$ and because the gamma distribution (f_Γ) is only defined for positive real numbers, we have $f_\Gamma(-S; -\beta_d, b)$. In eqs. 9-10, for deleterious mutations ($S \leq 0$), we had implicitly performed the change of variable S=-S, thus the boundary of the integration had also changed (e.g. $[1; +\infty]$ instead of $]-\infty; -1]$ for eq. 9). Although this change of variable (S=-S) in the integration is not incorrect, we agree that it is unnecessary and is confusing, thus we now show eqs. 9 and 10 without such change in variable.

- The authors bring up compensatory mutations a couple of times throughout the manuscript, including in a section of the discussion in which they discuss the potential conflation of putatively adaptive mutations and compensatory mutations in their study. They argue that the proportion of beneficial non-adaptive mutations observed in the present study supports that a model without epistasis as an adequate fit to the protein data (P8 L277-278). I would appreciate additional clarification of this argument, as on the surface it seems like a high proportion of observed beneficial non-adaptive mutations wouldn’t preclude an additional contribution of compensatory mutations. In addition, this makes me curious about whether the method presented, with the addition of a more complex protein fitness landscape, could be used to infer the relative contribution of compensatory mutations (with perhaps further implications for inferring the contribution of compensatory mutations to adaptive evolution, see eg. Storz 2018 “Compensatory mutations and epistasis for protein function”)?

Thank you for bringing up this interesting perspective. We updated the *Discussion* to include such an idea, which has now changed from:

"Our results imply that the fitness effects of new mutations are mostly conserved across mammalian orthologs, in agreement with other studies showing that for conserved orthologs with similar structures and functions, models without epistasis provide a reasonable estimate of fitness effects in protein coding genes [62, 63]."

to:

"Our results imply that the fitness effects of new mutations are mostly conserved across mammalian orthologs, in agreement with other studies showing that for conserved orthologs with similar structures and functions, models without epistasis provide a reasonable estimate of fitness effects in protein coding genes [62, 63]. Conceptually, the framework presented here, with the addition of a more complex protein fitness landscape at the phylogenetic scale, could be used to infer the relative contribution of compensatory mutations to non-adaptive and adaptive evolution."

- The authors point out the difficulty in distinguishing between beneficial non-adaptive mutations from convergent adaptation, which is an interesting point. I wonder if incorporating effective population size of the lineages that include such convergent/beneficial non-adaptive mutations could help to distinguish them, given the expected relationship between the proportion of mutations of different classes and effective population size?

We think that it is a promising idea that could indeed be realized. However, a clear theoretical expectation on how effective population size modulates the proportion of beneficial non-adaptive mutations is still lacking. We hope that by bringing attention to this question, this study will motivate researchers to work on this and other related questions and hopefully devise a way to distinguish beneficial non-adaptive mutations from convergent adaptation.

Reviewer III:

The paper concerns the prevalence of advantageous back mutations in molecular evolution. The authors present a method for estimating this prevalence. The method is based on fitting a model of molecular evolution where each site in a protein has a conserved set of amino acid preferences given as scaled fitness values. Then, currently segregating SNPs can be assigned scaled selection coefficients under this model. The site frequency spectrum of these same SNPs can be used to estimate the distribution of current selective pressures as a function of the predicted selection coefficients from the model of long-term amino acid preferences. This can then be used to estimate the fraction of beneficial new mutations that are restoring a historically preferred amino acid state versus the fraction of beneficial new mutations that are responding to new selective pressures. The authors apply the method exome-wide to a set of 80+ mammalian species for the model of molecular evolution and polymorphisms from over 20 populations. While the estimates for the prevalence of advantageous back mutations vary widely between populations, they generally constitute a substantial minority of all beneficial mutations.

The manuscript represents an innovative way of combining approaches from molecular evolution and population genetics to address an interesting but understudied problem (the prevalence of beneficial back mutations). The broad strokes approach is similar to a recent PNAS paper with the same lead author (Latrille, T., Rodrigue, N. & Lartillot, N. 2023 Genes and Sites under Adaptation at the Phylogenetic Scale Also Exhibit Adaptation at the Population-Genetic Scale. PNAS), but the question and methods here are distinct. The manuscript is a good fit for the readership of PLOS Genetics and the conclusions are of broad interest. That being said, I have some technical concerns about whether the proposed method is sound as currently implemented.

Major issues:

1. While the authors do a good job anticipating the effects of violations of model assumptions (which would tend to result in an underestimate of the frequency of beneficial back mutations), they do not show that their method is capable of producing accurate estimates when the model assumptions are satisfied. They should show this via simulations where the ground truth is known.

Although the core of the study is to test our approach on empirical data (mammalian population-genetics and phylogenetic datasets), we acknowledge and agree that simulations are important and necessary to test its performance. However, it is unfortunately out of reach to simulate the whole exome through the mammalian evolution using a Wright-Fisher simulator. Typically, a simulator accounting for every generation managed to simulate 10000 sites for the whole mammalian phylogeny, but took a month to run (C++ in Latrille *et al.*, 2021). Unfortunately, running such a simulation across the whole exome would require scaling x100 the computing resources.

Instead of such a brute-force approach, we performed simulations based on a population-genetic formalism to test whether our approach is able to recover the proportion of beneficial mutations that are non-adaptive in synthetic polymorphism datasets. More specifically, we performed simulations of site-frequency spectra, thus at the population-genetic scale, by using a mixture of the DFEs that is predicted from the phylogenetic mutation-selection model, and adding adaptive mutations to this DFE.

In fact, this strategy is taking inspiration from your idea in the next comment to fit a mixture of DFEs. In practice, simulations are performed under the Poisson Random Field (PRF) framework of population genetics (Sawyer & Hartl, 1992). Each simulation generates a synthetic synonymous SFS, and a synthetic non-synonymous SFS for the mutations in the category $x \in \{\mathcal{D}_0, \mathcal{N}_0, \mathcal{B}_0\}$.

Technically, for non-synonymous mutations, we first draw a random sample of the selection coefficients from the mixture of DFE using as parameter P[y|x] for $y \in \{\mathcal{D}, \mathcal{N}, \mathcal{B}\}$. Importantly $P[\mathcal{B}|\mathcal{D}_0]$ and $P[\mathcal{B}|\mathcal{N}_0]$ are considered adaptive mutations, and thus their selection coefficients have a selection coefficient of $S_{adaptive}$ (here =20). All the others are drawn for the DFEs that are predicted from the phylogenetic mutation-selection model. Synonymous mutations are considered neutral and have S=0. Given these selection coefficients, to obtain the expected SFS we integrate over the limiting density of frequency that mutations are segregating in the population, as described from eqs. 1-2 in Tataru *et al.* (2017). And finally, given this expected SFS, we perform a Poisson random sampling to obtain a sampled SFS of size n matching the total number of polymorphism to simulate.

In summary, the simulations generates a synonymous SFS, and a non-synonymous SFS for the mutations in the category $x \in \{\mathcal{D}_0, \mathcal{N}_0, \mathcal{B}_0\}$, and require as input the following:

- Total number of polymorphisms to simulate (synonymous and non-synonymous).
- The DFE of S_0 predicted from the phylogenetic mutation-selection.
- Estimates of P[y|x] for $y \in \{\mathcal{D}, \mathcal{N}, \mathcal{B}\}$.
- Selection coefficient for adaptive mutations, here $S_{adaptive}$=20.
- The sampled SFS size n, here n=16 (i.e. twice the number of sampled individuals).

From these two SFS (synonymous and non-synonymous), we run polyDFE to obtain $P[\mathcal{D}|x]$, $P[\mathcal{N}|x]$ and $P[\mathcal{B}|x]$. By doing so for the 3 categories $x \in \{\mathcal{D}_0, \mathcal{N}_0, \mathcal{B}_0\}$, we can ultimately compute $P[\mathcal{B}_0|\mathcal{B}]$ using the same equations as in the main manuscript (eqs. 14-15). We used 8 different set of parameters (P[y|x] and numbers of polymorphism) and simulated 10 replicates for each set of parameters. For each synthetic data we computed $P[\mathcal{B}_0|\mathcal{B}]$ which is then compared to the ground truth (calculated analytically from the set parameters P[y|x]). These simulations show that we are able to retrieve the proportion of non-adaptive mutations in those synthetic datasets.

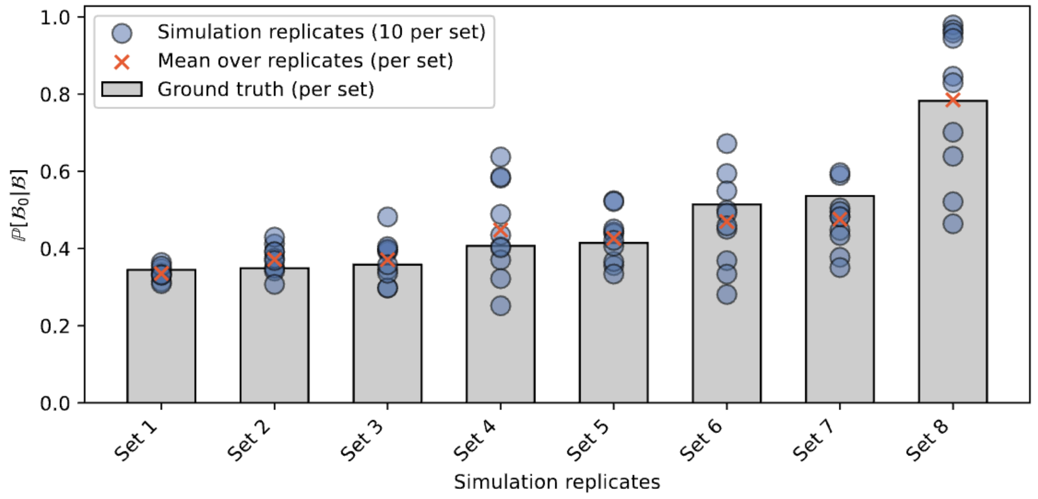

The python script to generate a PRF simulation is available in the github repository at:
https://github.com/ThibaultLatrille/SelCoeff/blob/main/scripts/simul_DFE.py
The pipeline to run and analyze the simulations is available in the github repository at:
https://github.com/ThibaultLatrille/SelCoeff/blob/main/workflow/Simulations.smk

Altogether, the simulation and their results are detailed in Supplementary Materials, section S7.3. We also updated the *Results* to mention these simulations:
"Second, we performed simulations at the population-genetic level and confirmed that our method was able to recover the proportion of beneficial mutations that are non-adaptive in synthetic polymorphism datasets (Supplementary Materials section S7.3, fig. S15)."

2. I am particularly concerned about the previous point because I suspect that the method has a problem as currently implemented. The problem is that the modeling of the DFE of the segregating variants is insufficiently expressive for the role that it plays in the inference. The DFE fit by the authors is a reasonable choice for the overall DFE but they are asking it to fit distributions that may look very different than the overall DFE (e.g. distributions truncated at -1 or +1). Fitting this grossly misspecified model will lead to highly inaccurate parameter estimates in some regimes.

Specifically, the DFE they use is a mixture between a reflected gamma distribution and an exponential distribution, which is a standard choice in the literature for estimating the whole DFE, and is roughly appropriate in that it can e.g. produce the peak at neutrality observed in nature and control the fractions of slightly deleterious mutations, etc. However, under the authors' procedure they split the SNPs into subsets based on their estimated selection coefficients from the phylogenetic model and then estimate the DFEs for these subsets individually. These DFEs will have a completely different shape from the overall DFE. For example, in the case that selective pressures are truly constant over time, the ground truth of the DFEs to be estimated would be a deleterious distribution right-truncated at -1, a neutral distribution truncated between -1 and +1, and a beneficial distribution truncated on the left at +1. Even to the extent that the authors' model of the DFE can put the right total probability in any one of these regions, the shape of the distribution in those regions will be completely wrong. For example, attempting to put most of the probability greater than +1 will also necessarily produce a DFE with enormously large selection coefficients because the positive portion of the DFE is constrained to be exponential.

Off the top of my head I do not know how to solve this problem. I believe the new fastDFE software from Thomas Bataillon's group allows you to define custom parametric models for the DFE. Another possibility might be to fit a mixture of the parametric DFE the authors are currently using and the truncated DFEs that would be predicted from the phylogenetic mutation-selection model (but I haven't thought it through).
We completely agree that modeling of the DFE could be misspecified (especially for $\mathscr{D}_0$ and $\mathscr{B}_0$ mutations), leading to inaccurate parameter estimates. Thus, we tested the performance of our method with three orthogonal approaches as described below.

First, as described in the previous comment, we have performed simulations at the population-genetic level to test whether our approach is able to recover the proportion of beneficial mutations that are non-adaptive in synthetic polymorphism datasets, as detailed in Supplementary Materials, section S7.3.

Second, as a control, we have also tested the method with a non-parametric DFE, which allows discarding the effect of gamma and exponentially constrained functional form while including substitutions in terminal lineages (see comment #5). We also included these experiments in the *Results* section, detailed in Supplementary Materials, section S11:
"Finally, we evaluated the effect of fitting a parametric functional form for the DFE As implemented in Tataru et al. (2017), the DFE is a mixture between a reflected gamma distribution and an exponential distribution (eq. 8, section 1.7 in Materials & Methods). Instead of using such a continuous DFE, we also tested our prediction with a non-parametric functional form for the DFE, obtaining estimates of $P[\mathcal{B}_0|\mathcal{B}]$ in the range of 8-94%, with a mean of 43% (tables S14-15)."

Third, we also performed another test to assess that fitting the same functional form of DFE to three categories of selection (i.e. $\mathcal{D}_0$, $\mathcal{N}_0$, $\mathcal{B}_0$) is a sound methodology. Typically, $P[\mathcal{B}]$ can be computed from two different estimates, by either splitting data by their category (i.e. $\mathcal{D}_0$, $\mathcal{N}_0$, $\mathcal{B}_0$) and fitting three DFE, or not splitting at all and fitting a single DFE.

In the case of splitting data by their category (i.e. $\mathcal{D}_0$, $\mathcal{N}_0$, $\mathcal{B}_0$), the one presented in the manuscript, $P[\mathcal{B}]$ is obtained by summing over the three category using the law of total probability (eq. 15):

$$P[\mathcal{B}] = P[\mathcal{B}|\mathcal{D}_0]\times P[\mathcal{D}_0] + P[\mathcal{B}|\mathcal{N}_0]\times P[\mathcal{N}_0] + P[\mathcal{B}|\mathcal{B}_0]\times P[\mathcal{B}_0]$$

But it is also possible to compute $P[\mathcal{B}]$ directly from fitting polyDFE to the full data, not splitting at all, and then $P[\mathcal{B}]$ is obtained as in eq. 11:

$$P[\mathcal{B}]=p_b \times \int_1^{+\infty} f_e(S;\beta_b)dS.$$

These two different estimates of $P[\mathcal{B}]$ allow us to test whether fitting the same functional form of DFE to three categories of selection is a sound methodology. We reproduced this experiment for $P[\mathcal{D}]$, $P[\mathcal{N}]$ and $P[\mathcal{B}]$ as shown below, and detailed in Supplementary Materials, section S10.6. Altogether, we also included these experiments in the *Results* section:
"We assessed the impact of fitting the same functional form of DFE to the three different categories of changes $\mathcal{D}_0$, $\mathcal{N}_0$ and $\mathcal{B}_0$. To this aim we computed the total amount of current selection by fitting either a single DFE on the whole dataset or by summing the other three independent DFEs. These disjoint estimates are well correlated, with a goodness of fit R^2={0.95, 0.89, 0.82} for respectively $P[\mathcal{D}]$, $P[\mathcal{N}]$ and $P[\mathcal{B}]$ (figs. S20-22)."

Figure S20: $\mathbb{P}[\mathcal{D}]$ when fitting a single or three DFEs.

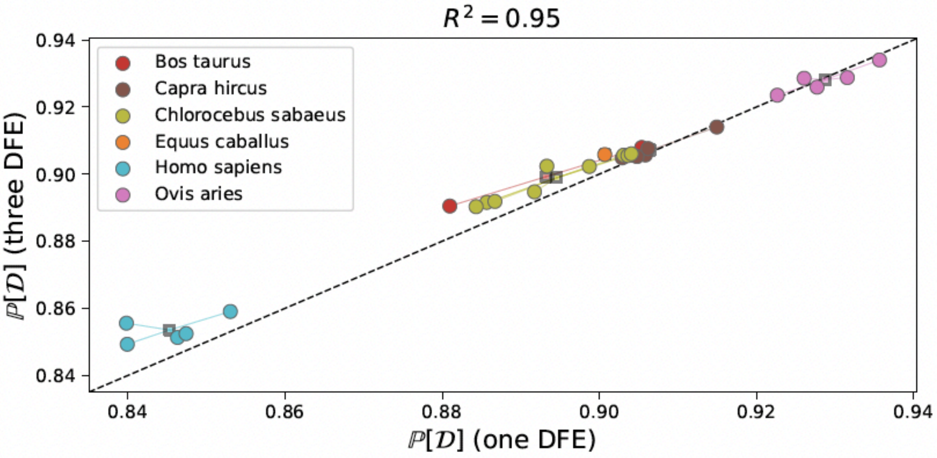

Figure S21: $\mathbb{P}[\mathcal{N}]$ when fitting a single or three DFEs.

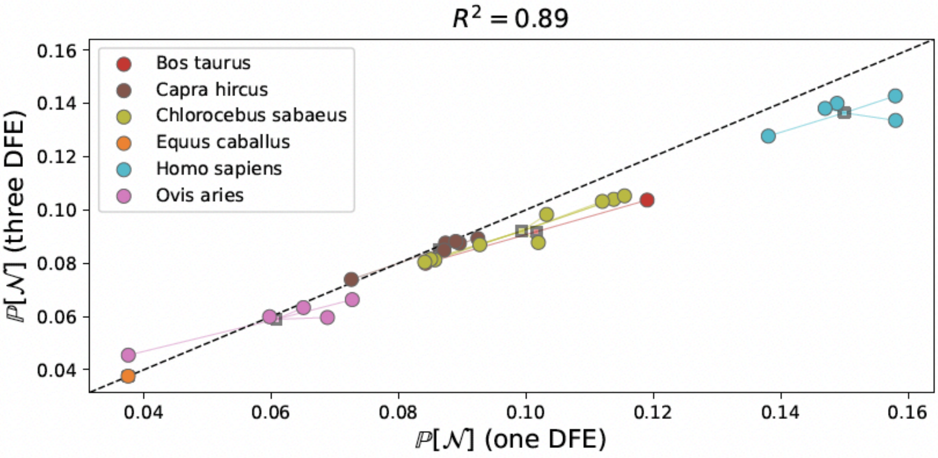

Figure S22: $\mathbb{P}[\mathcal{B}]$ when fitting a single or three DFEs.

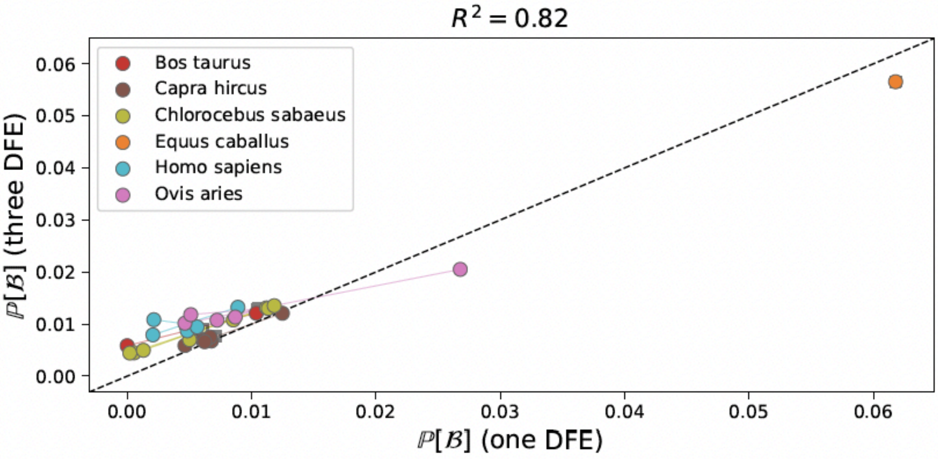

We also clarified the effect of model mis-specification by updating the *Discussion* from:
"To be conservative, we considered that any mutation under positive selection at the population scale ($\mathcal{B}$) but not predicted as such at the phylogenetic scale (not $\mathcal{B}_0$) is potentially an adaptation. In practice, we obtained different values of the proportion of non-adaptive beneficial mutations depending on the model used to infer the fitness effects at the population scale, and the filtering or not of CpG mutations (tables S8-14). It appears that our estimation can be sensitive to model misspecification and overall, while we provide an order of magnitude for the contribution of beneficial non-adaptive mutations to positive selection, methodological improvement on the estimation of the distribution of fitness effects is needed to increase the precision of this value."
to:
"In practice, we obtained different values of the proportion of non-adaptive beneficial mutations depending on i) the filtering or not of CpG mutations, ii) whether we included substitutions in the terminal lineage along with within-population polymorphisms to estimate fitness effects and iii) the model used to infer the fitness effects. It appears that our estimation can be sensitive to model misspecification and overall, while we provide an order of magnitude for the contribution of beneficial non-adaptive mutations to positive selection, methodological improvement on the estimation of the DFE is needed to increase the precision of this value."

3. There are several passages such as line 146-149 stating that dN/dS is "biased" as a measure of adaptation due to the presence of beneficial back mutations. As currently written this discussion appears misguided.

First, it is well-understood that as a practical matter dN/dS is massively biased AGAINST detecting adaptation because the signal from sites under purifying selection typically overwhelms the signal from sites experiencing positive selection. Obviously any discussion of subtle details of the behavior of d_N/d_S as a method of detecting adaptation has to be viewed against this backdrop.

We totally agree, we are sorry for the mistake. What we meant is that d_N/d_S as an estimate of **purifying selection** is always overestimated because of beneficial non-adaptive mutations. This can be a problem when different genes have different proportions of beneficial non-adaptive mutations depending on non-selective factors (e.g. Joseph 2024). We now corrected the *Results*:
"This result further indicates that using d_N/d_S as an estimate of adaptation is biased due to the presence of beneficial non-adaptive mutations among the non-synonymous substitutions. By discarding all beneficial non-adaptive mutations we can obtain an estimate of d_N/d_S which is not inflated."
to:
"This result further indicates that using d_N/d_S as an estimate of purifying selection is biased (overestimated) due to the presence of beneficial non-adaptive mutations among the non-synonymous substitutions. By discarding all beneficial non-adaptive mutations we can obtain an estimate of d_N/d_S which is not inflated."

- J. Joseph. Increased positive selection in highly recombining genes does not necessarily reflect an evolutionary advantage of recombination." *Molecular Biology and Evolution* (2024): msae107. https://doi.org/10.1093/molbev/msae107

Second, the authors come to their conclusion by calculating dN/dS for the subset of sites that they predict are maladapted at the start of the terminal branches under their phylogenetic model. Finding $d_N/d_S>1$ for these sites is 100% appropriate, since this subset of sites did truly adapt and the new amino acids at these sites really are closer to optimality at the end of the branch than at the beginning! The fact that the cause of the maladaptation is the previous fixation of deleterious mutations rather than environmental change does not change the fact that this subset of sites has contributed positively to the adaptedness of the population. It is just that under the stationary process the adaptation at these sites is offset by maladaptive substitutions elsewhere in the genome so that there is no net adaptation.
Third, there is a broader issue throughout the manuscript of verbally tying adaptation to environmental change. However, rapid evolution in genes with elevated d_N/d_S is often not driven by environmental change but rather by frequency-dependent selection, e.g. rapid evolution of reproductive genes and genes involved in sexual selection. In the mathematical framework used by the current authors, Mustonen and Lassig 2010 PNAS's treatment of these issues via the calculation of "fitness flux" provides a rigorous method for addressing these complications precisely in the setting of allelic preferences relevant to the methods described here (which for instance structurally cannot address issues of absolute fitness, reproductive excess, etc.). I think the more intuitive language used in the manuscript is OK as motivation, but the reader should be more explicitly directed to the mathematical theory for a rigorous treatment (and the authors may also want to update some of their views in light of this clearer understanding).
Regarding the comment computing d_N/d_S for $\mathcal{B}_0$ substitutions and finding $d_N(\mathcal{B}_0)/d_S>1$, we also took this opportunity to clarify the relationship between selection coefficient and d_N/d_S. Indeed, $\omega=d_N/d_S$ can be related to the underlying scaled selection coefficient (S) with the relation $\omega=S/(1-\exp(-S))$ as in *Nielsen & Yang* (2003, eq. 3). In our experiments, our observed $d_N(\mathcal{B}_0)/d_S$ thus translates into S values between 0.32 and 1.24, so indeed only slightly advantageous. Accordingly, we updated the section *Selection along the terminal branches* in the *Results* from:

"We obtained values between 1.17 and 1.75 in the different lineages (table S1), meaning that $\mathcal{B}_0$ mutations reach fixation slightly more frequently than synonymous mutations that are supposed to be neutral, consistent with these $\mathcal{B}_0$ mutations being weakly beneficial"

to:

"We obtained values between 1.17 and 1.75 in the different lineages (table S2), meaning that $\mathcal{B}_0$ mutations reach fixation slightly more frequently than synonymous mutations that are supposed to be neutral. Such observation is consistent with the premise that $\mathcal{B}_0$ mutations are weakly beneficial, translating to a scaled selection coefficient between 0.32 and 1.24 (table S2). Finding $d_N(\mathcal{B}_0)/d_S>1$ for these sites confirms that these sites are closer to optimality at the end of the branch than at the beginning. Even though the beneficial effect of these mutations does not come from an environmental change, it does not change the fact that they have contributed positively to the population's fitness. It is just that at mutation-selection-drift equilibrium, the increase in fitness at these sites is offset by deleterious substitutions elsewhere in the genome so that there is no net adaptation."

In general, we agree with the three points, and we are now explicitly referring to the mathematical theory of Mustonen & Lässig (2009, 2010) in several places of the *Discussion*. We also describe the definition of *fitness flux* in the section *Detecting adaptation above the nearly-neutral background*:

"Detecting adaptation above this background of nearly-neutral substitutions remains a challenge. First, one strategy is precisely to use a nearly-neutral substitution model as a null model of evolution."

to:

"We provide evidence that in mammalian orthologs, many substitutions occur through fixation of both deleterious mutations and beneficial non-adaptive mutations. Detecting adaptation above this background of substitutions remains a challenge. Mathematically, the surplus of positive selection due to an externally driven changing fitness landscape is called *fitness flux*, and requires experimentally measuring the selection coefficient of each mutation in each genetic background. Then, the *fitness flux* can be estimated if either the substitutions history is known (Mustonen & Lässig, 2009) or changes of frequency in currently segregating variants (Mustonen & Lässig, 2010). Without measured selection coefficients, another strategy is precisely to use a nearly-neutral substitution model as a null model of evolution."

4. The authors do a good job in addressing how epistasis due to interactions between specific sites such as structural contacts would play into their modeling framework but should also address epistasis that arises because mutations act additively but selection is nonlinear (e.g. stabilizing selection on an additive trait such as protein activity). The molecular evolution in the latter case is dealt with by Charlesworth 2013 "Stabilizing Selection, Purifying Selection, and Mutational Bias in Finite Populations" in Genetics.

We agree that an exhaustive discussion on the impact of epistasis could make the distinction between specific epistasis (mutation influences the phenotypic effect of few other mutations) and non-specific epistasis (nonlinear relationship between the phenotype and fitness) as described in Tyler & Thornton (2016). However, we also argue that the current paragraph on epistasis encompasses both specific and non-specific epistasis, albeit implicitly. Altogether, we updated the paragraph in the *Discussion* to make this distinction explicit, from:

"Our model assumes that amino-acid fitness landscapes are site-specific and also independent of one another, whereas under pervasive epistasis, the fitness effect of any mutation at a particular site would depend on the amino acids present at other sites. Epistasis has been shown to play a role in the evolution of protein-coding genes, with amino-acid residues in contact within a protein or between proteins tending to co-evolve [53–55]."

to:

"Our model assumes that amino-acid fitness landscapes are site-specific and also independent of one another, whereas under pervasive epistasis, the fitness effect of any mutation at a particular site would depend on the amino acids present at other sites. Epistasis is common for mutations that influence the protein's physical properties (e.g. conformation, stability, or affinity for ligands) or might arise due to nonlinear relationship between the protein's physical properties and fitness (Tyler & Thornton, 2016). Regardless of its origin, epistasis has been shown to play a role in the evolution of protein-coding genes, with amino-acid residues in contact within a protein or between proteins tending to co-evolve [53–55]."

Tyler N. Starr & Joseph W. Thornton.
Epistasis in protein evolution.
Protein science 25.7 (2016): 1204-1218.
https://doi.org/10.1002/pro.2897

5. I think there is a missed opportunity in the analysis of substitutions along terminal branches to provide an estimate of the total frequency of amino acid substitutions due to mutation-selection-drift balance. Because the model of molecular evolution used is time reversible, the distribution of mutations fixed at stationarity is symmetric around zero. The lack of symmetry along the terminal branches then provides a means to estimate the frequency of beneficial back substitutions (in contrast to the rest of the study which estimates the frequency of beneficial back mutations). For example, one could use twice the fraction of substitutions with selection coefficients greater than 1 as an estimate of this rate; more refined estimates are also likely possible. Likewise, the fraction of deleterious substitutions minus the fraction of advantageous substitutions provides a crude estimate of the fraction of substitutions due to adaptation to new environmental conditions.
We completely agree that substitutions along terminal branches provide a great source of data to strengthen our results. Thus rather than relying solely on currently segregating mutations to quantify selection, we also leveraged both polymorphism within a population together with substitutions in the terminal lineage to estimate the distribution of fitness effects (DFE), and we have added a new *Results* section, *Selection in the terminal lineage and in populations*, that describes these results.

More generally, the highly consistent estimates of the frequency of beneficial back substitutions in the last column of table S1 (very tight between .09-.12) suggests an overall frequency of nearly neutral substitutions due to consistent long-term selection pressures, mutation and drift of approximately 20% of all substitutions (doubling the last column of table S1 to count the corresponding deleterious fixations). This provides additional evidence for the importance of beneficial but not adaptive mutations.
Thank you very much for this point that we did miss in the manuscript, which provides additional evidence for the importance of beneficial but not adaptive mutations. We have added the following paragraph in *Results*:
"Of note, if we were to assume a stationary mutation-selection-drift equilibrium in the terminal lineage, we would expect a symmetric proportion of positively ($\mathcal{B}_0$) and negatively ($\mathcal{D}_0$) selected substitutions if there were no adaptation. The lack of symmetry along the terminal branches then provides a means to estimate the frequency of non-adaptive beneficial substitutions. Mathematically, twice the fraction of $\mathcal{B}_0$ substitutions is an estimate of this rate. This rate is highly consistent across lineages (table S1) and suggests an overall frequency of nearly neutral substitutions due to consistent long-term selection pressures, mutation and drift of approximately 20% of all substitutions (18%-24% across species)."

Minor issues:

- Line 167, perhaps also helpful to cite Grimm et al. 2015 here

We completely agree and have included this reference, changing the sentence from:

"Although this classification also relies on deep protein alignments and therefore cannot be considered an independent result from our own, it does provide a consistency check if the effect of a mutation on human health is in line with its fitness effect predicted by our method." to:

"Although this classification also relies on deep protein alignments and therefore cannot be considered an independent result from our own, it does provide a consistency check if the effect of a mutation on human health is in line with its fitness effect predicted by our method (Grimm *et al.,* 2015)."

- I think readers would appreciate a little more discussion on the relationship between these results and the McDonald-Kreitman test literature, since both are integrating fixed and segregating variation, and relate to a fraction of adaptive mutations/substitutions.

We updated the discussion to discuss the McDonald-Kreitman literature, changed from:

"A long-standing debate in molecular evolution is whether the variation we observe between species in protein-coding genes is primarily due to nearly-neutral mutations reaching fixation by drift or primarily due to adaptation. Here we provide evidence that in mammalian orthologs, many substitutions occur through fixation of both deleterious mutations and beneficial non-adaptive mutations. " to:

"A long-standing debate in molecular evolution is whether the variation we observe between species in protein-coding genes is primarily due to nearly-neutral mutations reaching fixation by drift or primarily due to adaptation. Measuring the "rate of adaptation" in proteins, as pioneered by McDonald & Kreitman [5], has been central to inform this debate [72]. However, the McDonald & Kreitman test detects signatures of accelerated evolution in a given terminal branch compared to an expectation based on polymorphism present in the population. It considers the fraction of substitutions that fix too quickly as "adaptive" [5–7] despite there being other processes that can lead to their fixation [73–75] and some of these substitutions being beneficial but non-adaptive [12–14, 17]. Here, the expectation is built on the pattern of substitutions across a phylogeny compared to the fitness effects that can be estimated from both substitutions in a terminal lineage and polymorphism in populations. Moreover, the goal is not to detect a fraction of beneficial substitutions (i.e. "adaptive" substitutions for McDonald & Kreitman [5]) but to estimate the proportion of non-adaptive mutations among beneficial ones."

74. Rousselle, M., Laverré, A., Figuet, E., Nabholz, B. & Galtier, N. Influence of Recombination and GC-biased Gene Conversion on the Adaptive and Nonadaptive Substitution Rate in Mammals versus Birds. *Molecular Biology and Evolution* 36, 458–471 (2019).

75. Joseph, J. Increased Positive Selection in Highly Recombining Genes Does Not Necessarily Reflect an Evolutionary Advantage of Recombination. *Molecular Biology and Evolution*, msae107 (2024).

76. Ohta, T. & Gillespie, J. H. Development of Neutral and Nearly Neutral Theories. *Theoretical Population Biology* 49, 128–142 (1996).

- I suggest also discussing the alternative approach of considering adaptation to new selective environments by allowing selective preferences to change on subsets of branches (e.g. Kazmi and Rodrigue 2019, or Ritchie, Stark and Liberles 2021) and the strengths / weaknesses of this versus the current approach.

Thank you, this is a great suggestion. We have now discussed and mentioned the different approaches to model adaptation in *Discussion,* where we have changed from:

"Instead of relying solely on summary statistics (such as d_N/d_S or ω_0), another strategy to detect adaptation is to include changes in the fitness landscapes inherently within the mutation selection framework [72]. Such mechanistic models could be more general than site-specific fitness landscapes, including epistasis and changing fitness landscapes [57, 73]."

to:

"Instead of relying solely on summary statistics (such as d_N/d_S or ω_0), another strategy to detect adaptation is to include changes in the fitness landscapes inherently within the mutation selection framework, either with small changes along the phylogeny (Tamuri & dos Reis, 2021) or either by allowing fitness to change on subsets of branches (Kazmi and Rodrigue, 2019; Ritchie et al., 2021). Such mechanistic models could be more general than site-specific fitness landscapes, including epistasis and changing fitness landscapes (Goldstein, & Pollock, 2017; Stolyarova et al., 2023)."
