# Supplementary material for: Estimating the proportion of beneficial mutations that are not adaptive in mammals
Source: PLoS Genet. 2024 Dec 26;20(12):e1011536. doi: 10.1371/journal.pgen.1011536 (PMC11709321; doi:10.1371/journal.pgen.1011536)
Supplement: Attachment — Submitted filename: PGENETICS-D-24-00471R2-Response-to-reviewers.pdf [file pgen.1011536.s006.pdf]

Response to editor and point-by-point response to reviewers' comments on Latrille *et al.*

Revision #2

Editor:

Thank you for submitting the revised manuscript. The reviewers and I all agree that it substantially improved. Before we move forward with your manuscript, please address the comments from Reviewer 3 about the Supplementary Information.

Dear Editor,

We thank you and the reviewers for the evaluation of our revised manuscript and the positive feedback on our revisions. We completely acknowledge that the Supplementary Information required improvements to ensure it meets a publication-ready standard. As such, each figure and table now includes a detailed caption that clearly explains the content and provides relevant context. We have reviewed the entire SI for consistency and completeness, removing bullet points and ensuring all figures, tables, and sections are fully explained. We believe these changes significantly enhance the clarity and usability of the SI and hope that these revisions address Reviewer 3's concerns.

Reviewer #1: The authors have carefully considered and responded to all of my previous comments, and I believe the revisions have enhanced the manuscript.

Reviewer #2: The authors have completed a thorough and substantial review in response to my and the other reviewers' comments.

I appreciate the additional discussion of predictions at the population vs phylogenetic level and the fitness seascape, as well as the manner in which they addressed the implications of N_e under multiple circumstances. The manuscript is now also easier to follow.

Reviewer #3: The authors have conducted a thorough and thoughtful revision, both with respect to my comments and the comments of the other reviewers, and have conducted numerous additional analyses that bolster the conclusions of the manuscript.

I do suggest some additional minor changes to the SI for formatting and completeness. Specifically, the SI is written as more of an outline than a publication-ready document. For example, it mostly has lists of bullet points below the figures rather than figure captions. Also, I think that many of the supplemental figures could use more explanation and be more self-contained. For instance, I assume that Figure S10 is using the same convention as main text Figure 4 that the circles denote populations and the squares denote species means, but there isn't a caption and the bulleted text doesn't explain this either. Likewise, section 7.1 is

just a table (plus bullet points acting as a caption) with no explanation as are several of the other SI suggestions. Overall I think the SI should be brought up to a publication-ready standard.

We completely acknowledge that the Supplementary Information required improvements to ensure it meets a publication-ready standard. As such, each figure and table now includes a detailed caption that clearly explains the content and provides relevant context. We have reviewed the entire SI for consistency and completeness, removing bullet points and ensuring all figures, tables, and sections are fully explained.

Minor comments:

SI Typo “Additionally to including divergence, with also tested our prediction with polyDFE model D instead of model C” with->we

Corrected.
